# Iron-Doped ZnO Nanoparticles as Multifunctional Nanoplatforms for Theranostics

**DOI:** 10.3390/nano11102628

**Published:** 2021-10-06

**Authors:** Marco Carofiglio, Marco Laurenti, Veronica Vighetto, Luisa Racca, Sugata Barui, Nadia Garino, Roberto Gerbaldo, Francesco Laviano, Valentina Cauda

**Affiliations:** Department of Applied Science and Technology, Politecnico di Torino, C.so Duca degli Abruzzi 24, 10129 Turin, Italy; marco.carofiglio@polito.it (M.C.); marco.laurenti@polito.it (M.L.); veronica.vighetto@polito.it (V.V.); luisa.racca@polito.it (L.R.); sugata.barui@polito.it (S.B.); nadia.garino@polito.it (N.G.); roberto.gerbaldo@polito.it (R.G.); francesco.laviano@polito.it (F.L.)

**Keywords:** ZnO, iron doping, piezoelectricity, magnetic NPs, theranostics, cancer treatment

## Abstract

Zinc oxide nanoparticles (ZnO NPs) are currently among the most promising nanomaterials for theranostics. However, they suffer from some drawbacks that could prevent their application in nanomedicine as theranostic agents. The doping of ZnO NPs can be effectively exploited to enhance the already-existing ZnO properties and introduce completely new functionalities in the doped material. Herein, we propose a novel synthetic approach for iron-doped ZnO (Fe:ZnO) NPs as a multifunctional theranostic nanoplatform aimed at cancer cell treatment. Pure ZnO and Fe:ZnO NPs, with two different levels of iron doping, were synthesized by a rapid wet-chemical method and analyzed in terms of morphology, crystal structure and chemical composition. Interestingly, Fe:ZnO NPs featured bioimaging potentialities thanks to superior optical properties and novel magnetic responsiveness. Moreover, iron doping provides a way to enhance the electromechanical behavior of the NPs, which are then expected to show enhanced therapeutic functionalities. Finally, the intrinsic therapeutic potentialities of the NPs were tested in terms of cytotoxicity and cellular uptake with both healthy B lymphocytes and cancerous Burkitt’s lymphoma cells. Furthermore, their biocompatibility was tested with a pancreatic ductal adenocarcinoma cell line (BxPC-3), where the novel properties of the proposed iron-doped ZnO NPs can be potentially exploited for theranostics.

## 1. Introduction

Novel therapeutic approaches based on the use of smart nanomaterials are considered the frontiers in the development of next-generation, multifunctional nanosystems aiming for nanomedicine applications. Examples include theranostic nanoparticles (NPs), i.e., nanosystems capable of combining therapeutic and diagnostic functionalities to deliver and activate a therapeutic agent in a specific position inside the body and to report the status of the disease and/or the localization of the therapeutic agent at the same time [1].

One of the main and most appealing applications of theranostics is surely in the field of antitumoral treatments [2]. Indeed, the possibility to exploit both imaging capabilities and a therapeutic action of the theranostic agent in the human body, as well as to guide it toward the specific site of interest, gathers relevant advantages in terms of personalized medicine, allowing the application of a customized therapy and real-time diagnosis solely for the targeted organ or tissue.

In this regard, the study of theranostic nanoparticles gained relevant attention. Thanks to the reduced size, NPs represent one of the most suitable systems compatible with the cellular dimensions. This aspect is also efficiently combined with their superior physical and chemical properties thanks to quantum-size effects typical of nanometer-sized materials, which can be further customized depending on the nanomaterial’s shape and typology (metal, semiconductor, etc.). NPs also show passive tumor targeting, or even active targeting possibilities thanks to biomolecule grafting [3], as well as promising imaging potentialities [4]. Therefore, these aspects corroborate the use of NPs as optimal and versatile platforms to be employed for various therapeutic purposes, including drug delivery [5] and stimuli-responsive applications, i.e., hyperthermia [6], photodynamic [7,8] or sonodynamic therapies [9,10,11]. In the specific class of metal oxide nanomaterials, zinc oxide nanoparticles (ZnO NPs) are surely promising candidates and have been already proposed for nanomedicine [12,13,14]. First of all, ZnO NPs are extremely versatile in terms of existing techniques for NPs’ preparation and of the resulting nanoparticle morphology, making easier the tailoring of the NP system for specific theranostic applications [12]. Indeed, precipitation, spray pyrolysis, hydrothermal, solvothermal and microwave synthesis, as well as electrochemical and sol–gel methods, can be used to obtain ZnO NPs [15,16,17]. Moreover, ZnO NPs present very interesting optical properties, which have been proven to impart a photocatalytic activity and, in turn, promising antimicrobial behaviors [18].

ZnO is also a well-known piezoelectric material. Piezoelectric NPs have been already explored in nanomedicine either as powerful systems for tissue engineering [19,20] or as promising anticancer therapeutics [21]. The efficacy of both these antithetic approaches relies on the interference that electrical stimuli, generated by the mechanically activated piezoelectric NPs, may induce on cell ion homeostasis. If opportunely tuned, this interference may lead to cell differentiation or to cell anticancer drug sensitization. Based on the above-mentioned mechanism, ZnO piezoelectricity has been successfully exploited to develop several smart ZnO-based materials for hard and soft tissue regeneration [12,22].

From the therapeutic standpoint, it has also been demonstrated that ZnO NPs can act as sensitizing agents in photo- and sonodynamic therapy. Thanks to the enhanced reactive oxygen species generation when exposed to light [8] or mechanical stimuli [23], ZnO NPs are effective in killing cancer cells when coupled with ultraviolet (UV) light radiation or shock waves [9].

However, ZnO NPs in their pure form present some criticalities that should be considered in view of their translation to clinically relevant applications. The most important one is their toxicity. As a matter of fact, despite being considered Generally Recognized as Safe (GRAS) in its bulk form by the Food and Drug Administration, nanosized ZnO demonstrates a dose-dependent toxicity [24] that can be related to three main processes: (i) the release of zinc cations due to NP dissolution in biological media, which may lead to the disruption of the cell homeostasis [25]; (ii) the generation of cytotoxic reactive oxygen species induced by photocatalysis and sonoirradiation [26]; and finally, (iii) the mechanical damages that the NP could induce on the cell membrane during internalization [18]. Moreover, ZnO on its own is a wide-bandgap semiconductor (approximately 3.4 eV [27]), and it can only absorb light in the UV region of the spectrum, thereby limiting its use as photosensitizing agent for photodynamic therapy. Indeed, ZnO NPs are able to generate ROS under photoirradiation and kill tumoral cells only when exposed to UV light, which is, however, harmful also for healthy cells.

Therefore, both the therapeutic efficacy and the imaging abilities of ZnO NPs need to be properly optimized to account for the use of small and safe doses of these theranostic nanoparticles.

Among the various options, doping can be a good strategy to address this difficult goal [26]. For example, doping is a powerful method to modify the electronic and optical properties of semiconducting materials such as ZnO. In fact, doping is a well-known technique exploited in several fields, including optical and bandgap engineering. The insertion of doping ions results in the formation of additional defect states in the energy band structure and of new electronic transitions among energy levels. Consequently, a reduction in the bandgap can thus be obtained also for ZnO, extending the corresponding absorption spectral region from the UV to visible light in the best-case scenario. The appearance of new luminescence peaks useful for imaging purposes [28] can be obtained as well. Additionally, doping is expected to properly modify the crystal structure of ZnO nanomaterials. This aspect, coupled with a proper modulation of the chemical oxidation state of the introduced dopant, has been proved to enhance the piezoelectric response with respect to the pure ZnO [29,30,31].

Finally, doping is a powerful method to make ZnO a magnetic material [26]. Indeed, in its pure form, ZnO presents a diamagnetic behavior at room temperature [32,33]. However, it is possible to induce a diamagnetic to paramagnetic or ferromagnetic transition by introducing selected atoms, such as manganese [34,35], gadolinium [36] or iron (Fe) [37], that are able to carry magnetic dipoles to the system [38]. In these cases, the doped ZnO NPs revealed interesting magnetic behaviors and, hence, bio-imaging properties particularly useful for magnetic resonance imaging (MRI) applications.

Among the various doped ZnO nanomaterials, the iron-doped ones play a relevant role in the biomedical field. In particular, it has been proven that Fe doping reduces the dissolution rate of ZnO nanoparticles in several biological media, hence representing a valuable approach to partially prevent undesirable cytotoxic effects against healthy cells [39]. Despite Fe:ZnO nanomaterials being already reported in the literature, to the best of our knowledge, a comprehensive study focusing on the corresponding optical, piezoelectric, magnetic and biocompatible properties aimed at theranostics has not been reported yet.

Therefore, the aim of the work presented herein was to develop multifunctional iron-doped ZnO nanoparticles (Fe:ZnO NPs) as theranostic nanoplatforms that can be introduced safely inside the cell and that present intrinsic imaging and therapeutic potentialities to fight different types of cancer cells.

For this purpose, undoped ZnO and Fe:ZnO NPs incorporating two different dopant amounts (6 and 12 at.%) were synthesized with a wet chemical synthesis technique using oleic acid as a capping agent to stabilize the nanoparticle system in aqueous media. A further functionalization with amino-propyl groups was performed with a postsynthetic grafting approach for further dye labeling and to improve the NP stability in water media. All the NPs were investigated in terms of morphology and crystallographic structure to assess the distortion that doping induces on the ZnO crystal lattice. The chemistry of the NPs was investigated by spectroscopic techniques to define the amount of doping level and Fe oxidation state, as well as to verify their correct functionalization.

The nanoparticles were analyzed in terms of optical, electromechanical and magnetic properties to establish the improved performances of the doped particles with respect to the undoped ones. Indeed, Fe doping provides magnetic responsiveness to the doped particles, which could be used in magnetic resonance imaging. Moreover, by changing the amount of the inserted dopant, substitutional Fe ions with different radii and oxidation states relative to Zn^2+^ were noticed, finally influencing the electromechanical response of the doped NPs.

The NPs’ toxicity and cellular uptake were analyzed in vitro by evaluating their interaction with suspension cell lines, namely healthy and cancerous hematic cells (B lymphocytes and Burkitt’s lymphoma Daudi cell lines, respectively), showing an improved selectivity of the synthesized NPs toward cancerous cells and intrinsic therapeutic potentialities. In order to evaluate the effects of our NPs on a different cellular system, an adherent BxPC-3 pancreatic adenocarcinoma cell line was also tested. We also defined which atomic percentage of Fe doping in nanoparticles could be the best choice from the intracellular imaging and therapy standpoints. We proved that with the BxPC-3 cell line, Fe doping did not negatively affect the cytotoxicity of the particles in a significant way. In this perspective, our results allow proposing a nanosystem in which the novel properties gained by doping, i.e., luminescence, magnetism and piezoelectricity, can be fruitfully exploited for theranostic applications.

## 2. Materials and Methods

### 2.1. ZnO and Fe:ZnO Nanoparticle Synthesis Procedure

Undoped and iron-doped zinc oxide nanoparticles (ZnO and Fe:ZnO NPs) were synthesized by a wet chemical process, exploiting oleic acid as stabilizing agent [35].

In more detail, for ZnO NPs, 526 mg of zinc acetate dihydrate (Zn(CH_3_COO)_2_⋅2H_2_O, ACS Reagent, ≥99.0%, Sigma-Aldrich, Darmstadt, Germany) was dissolved in 40 mL of ethanol (99%, Sigma-Aldrich). The ethanolic solution was placed in a 100 mL round-bottom flask. Then, 1 mL of bidistilled water (obtained from a Direct Q3 system, Millipore, Burlington, MA, USA) and 140 μL of oleic acid (≥99%, Sigma-Aldrich) were added to the solution.

The flask was placed in a silicon oil bath to be heated up to 70 °C in refluxing conditions.

In the meanwhile, 1.044 g of tetramethylammonium hydroxide pentahydrate (TMAH, 98.5%, Sigma-Aldrich) was dissolved in 10 mL of ethanol and 1.052 mL of bidistilled water. The TMAH solution was rapidly poured into the main solution after 10 min of moderate stirring at 70 °C.

After a further 10 min, during which the clear solution turned into an opaque particle dispersion, 40 mL of ice-cooled ethanol was included in the solution to stop the reaction. The flask was then placed in an ice bath for 3 min.

The resulting NPs were washed twice by centrifuging them at 8000× *g* for 10 min and then resuspended in ethanol.

Similarly, Fe:ZnO NPs were obtained by including 58 or 116 mg of ferric nitrate nonahydrate (Fe(NO_3_)_3_⋅9H_2_O, HiMedia) for 6 at.% (Fe6:ZnO) and 12 at.% (Fe12:ZnO) doped nanoparticles, respectively, in the zinc acetate ethanolic solution.

### 2.2. ZnO and Fe:ZnO Nanoparticle Functionalization Procedure

Amino-propyl functionalization of the undoped and iron-doped ZnO nanoparticles was carried out prior to in vitro biological tests, following the procedure described by some of us [40]. In particular, 40 mg of NPs were dispersed in ethanol to obtain a 2.5 mg/mL dispersion. Then, the dispersion was placed in a 25 mL round-bottom flask and heated up to 70 °C in refluxing conditions, under moderate stirring and continuous gaseous nitrogen flux. After 10 min, 10 mol% of 3-aminopropyltrimethoxysilane (APTMS) was added to the nanoparticle dispersion. The system was kept in a nitrogen atmosphere and continuously stirred for 6 h and then washed two times with ethanol by a centrifugation and redispersion process (14,000× *g* for 10 min).

### 2.3. Physicochemical Characterization

The morphology and chemical composition of the prepared materials were characterized by field emission scanning electron microscopy (FESEM, SUPRA 40 from Zeiss, Oberkochen, Germany) coupled with a detector for energy-dispersive X-ray spectroscopy (EDS, x-act 10 mm^2^ Silicon Drift Detector from Oxford Instruments, Oxford, UK). The sample was prepared by spotting 10 µL of NP colloidal suspension (100 µg/mL) in water on a flat silicon substrate that could then be attached to the FESEM/EDS aluminum sample holder with conductive carbon biadhesive tape. The analysis was then performed on the dried sample.

Fourier transform infrared (FT-IR) spectroscopy was performed in transmission mode on 500 μg of ZnO and Fe:ZnO NPs deposited onto a silicon wafer, before and after functionalization, in the region 4000–400 cm^−1^ range with a Nicolet 5700 FT-IR spectrometer (Thermo Fisher, Waltham, MA, USA).

The crystallinity of the synthesized nanoparticles was investigated by X-ray diffraction (XRD) analyses with a Panalytical X’Pert diffractometer in θ–2θ Bragg–Brentano mode (Cu-Kα radiation source, λ = 1.54 Å, 40 kV and 30 mA) on samples prepared similarly to the ones exploited during FT-IR measurements. The evaluation of the peak shift was performed by fitting the peaks with a Gaussian function (Origin, OriginLab) and comparing the evaluated peak positions for the three main reflections. The same fit was exploited to determine the crystallite dimension (D) according to the Debye–Scherrer formula [41]:

(1) $$D=\frac{180\cdot \kappa \cdot \lambda }{\pi \cdot \Delta 2\theta cos\left(\theta \right)}$$

where *κ* = 0.89, *λ* is the X-ray radiation wavelength, Δ2*θ* is the full width at half maximum (FWHM) expressed in radians and *θ* is the diffraction angle.

X-ray photoelectron spectroscopy (XPS) was carried out with a PHI 5000 VersaProbe (Physical Electronics) system. The X-ray source was monochromatic Al-Kα radiation (1486.6 eV energy). The relative atomic concentration (at.%) of each chemical element was calculated from the high-resolution (HR) spectra. XPS spectra were analyzed using CasaXPS software (version 2.3.18). All the XPS spectra were processed after Shirley background subtraction. HR core-level spectra deconvolution into individual mixed Gaussian–Lorentzian peaks was obtained after binding energy (BE) calibration according to C1s position for adventitious carbon (284.8 eV).

Dynamic light scattering (DLS) and Z-potential measurements were performed to evaluate the size and surface charge of nanoparticles in colloidal solution with a Zetasizer Nano ZS90 (Malvern Panalytical, Malvern, UK). For this purpose, nanoparticle suspensions at the concentration of 100 μg/mL were prepared after 10 min of sonication with an ultrasound bath (40 kHz, Branson 3800 CPXH, Branson Ultrasonics Corporation) in both ethanol and bidistilled water (from a MilliQ system, Millipore, Burlington, MA, USA). DLS measurements were performed to determine the hydrodynamic radius of the particles both in their storage solvent (ethanol) and in the aqueous medium (water). Furthermore, Z-potential measurements were performed in bidistilled water to investigate the surface charge of the nanoparticles.

The optical absorption properties of ZnO and Fe:ZnO nanoparticles in the ultraviolet and visible region of the light spectrum were evaluated in transmission mode through a double-beam Varian Cary 5000 UV-vis-NIR spectrophotometer. In more detail, ZnO and Fe:ZnO NPs were suspended in ethanol at a concentration of 2 mg/mL and placed in quartz cuvettes (350 μL volume, 1 mm optical path length). The analysis was performed using a pure ethanol sample as baseline curve. All the spectra were thus background subtracted. From the UV-recorded spectra, the optical bandgap of the nanoparticles was estimated according to Tauc’s plot.

Fluorescence excitation and emission spectra were measured with a Perkin Elmer LS55 fluorescence spectrometer. ZnO nanoparticles were suspended in ethanol at a concentration of 1 mg/mL and placed in quartz cuvettes.

The magnetic characterization of ZnO and Fe:ZnO NPs was performed at room temperature and in quasistatic conditions with a DC magnetometer (Lake Shore 7225, Lake Shore Cryotronics, Inc., Westerville, OH, USA) equipped with a cryogen-free magnet system. To avoid any movement of the NPs during the application of a large magnetic field, 1 mg of NPs was encapsulated into 100 μL of Durcupan ACM and thermally treated at 60 °C for 3 days to harden the resin. Measurements were then performed on these samples.

The electromechanical response of ZnO and Fe:ZnO nanoparticles was evaluated with a Piezo Evaluation System (PES, TFAnalyzer 2000HS, Aixacct, Aachen, Germany). The nanoparticle suspension (3 mg dispersed in 200 μL of bidistilled water) was deposited on a conductive Au-SiO_2_ silicon substrate. After solvent evaporation, a continuous nanoparticle film could be formed. The measurements were performed under the application of a triangular excitation signal (voltage amplitude ±20 V, frequency 100 Hz).

### 2.4. Biological Characterization

#### 2.4.1. Suspension Cell Lines

Burkitt’s lymphoma cell line, Daudi (ATCC, CCL-213), was cultivated with RPMI 1640 (ATCC) supplemented with 10% heat-inactivated fetal bovine serum (FBS, ATCC). B lymphocytes, IST-EBV-TW6B (IRCCS AOU San Martino), were cultured with Advanced RPMI 1640 (Gibco), supplemented with 20% heat-inactivated fetal bovine serum (FBS, Gibco) and 1% L-glutamine (Lonza). All the cell line media were completed with 100 μg/mL streptomycin and 100 units/mL penicillin (Sigma).

#### 2.4.2. Adherent Cell Line

Pancreatic adenocarcinoma BxPC-3 (ATCC CRL-1687) cell line was cultivated with RPMI 1640 medium (ATCC) supplemented with 10% heat-inactivated fetal bovine serum (FBS, ATCC), 100 μg/mL streptomycin and 100 units/mL penicillin (Sigma).

All cell lines were grown at 37 °C with 5% CO_2_ atmosphere.

#### 2.4.3. Cytotoxicity

Daudi and B lymphocyte cells were seeded with a concentration of 2.0 × 10^5^ cell/mL in a 96-well plate for suspension culture (Greiner Bio-One) in 100 μL of volume per well, with cell culture media containing different doses (0, 10, 20, 30 and 40 μg/mL) of ZnO, Fe6:ZnO and Fe12:ZnO NPs and incubated at 37 °C in 5% CO_2_ atmosphere for 24 h. Backgrounds containing NPs with no cells were also prepared at the same time. To prepare the dispersions, the NP ethanolic stock solutions were sonicated in an ultrasound bath (Branson 3800 CPXH) for 10 min. Afterward, 200 μg of NPs were withdrawn and centrifuged for 10 min at 14,000× *g*. The supernatant was discarded and the NP pellet was dispersed in 200 μL of fresh medium with the help of a sonication bath, obtaining a solution of 1 mg/mL used to prepare the aliquots for cell treatment.

To evaluate the cytotoxicity of ZnO and Fe:ZnO NPs on the adherent cell line, BxPC-3, cells were seeded in each well of a 96-well culture plate (TC-Treated, Corning, Corning, NY, USA) with 100 μL of cell culture medium having a concentration of 2.5 × 10^4^ cells/mL. After their incubation at 37 °C in 5% CO_2_ atmosphere for 24 h, the cell culture medium was removed to be substituted with a fresh medium having different doses (0, 10, 20, 30 and 40 μg/mL) of ZnO, Fe6:ZnO and Fe12:ZnO NPs. Backgrounds containing NPs with no cells were also prepared at the same time.

Twenty-four hours after NP administration, the proliferation of Daudi, B lymphocytes and BxPC-3 cells was evaluated by the WST-1 proliferation assay. In particular, 10 μL of WST-1 (Roche) was added to the wells after 20 h for Daudi and IST or 22 h for BxPC-3. The absorbance was measured with a Multiskan GO microplate spectrophotometer (Thermo Fisher Scientific) at 450 nm using 620 nm as reference wavelength. All the backgrounds were subtracted from the absorbance value obtained for the corresponding sample. Measurements obtained for cells cultivated with 0 μg/mL NP dose culture medium were considered as control and set to 100% viability.

All the measurements were taken at least in triplicates. The analysis of variance (ANOVA) tests was performed with Origin (OriginLab), and the results are reported in Supplementary Materials.

#### 2.4.4. Uptake

Daudi and B lymphocyte cells were seeded in 24-well culture plates (Greiner Bio-One, Kremsmunster, Austria) according to the cytotoxicity protocol reported before and scaling the volume per well to 500 μL. Adherent BxPC-3 cells were cultured at a concentration of 6 × 10^5^ cells/mL (500 μL of volume per well) and seeded in each well of a 24-well plate (TC treated, Thermo Fisher). For uptake experiments, the NPs were labeled with ATTO647-NHS ester (as described elsewhere [13]) and dispersed at 10 and 20 μg/mL in cell culture medium.

After 5 and 24 h, suspension cells were collected and washed twice by centrifugation process at 140× *g* for 5 min with phosphate saline buffer (PBS) in order to remove the noninternalized NPs. On the other hand, adherent cells were rinsed twice with PBS, detached by trypsinization, collected and centrifuged at 130× *g* for 5 min.

After the last centrifugation process of each cell line, the supernatant was removed and 300 μL of PBS was employed to resuspend cell pellets prior to their analysis with a Guava Easycyte 6-2L flow cytometer (Merck Millipore, Burlington, MA, USA).

The number of events, corresponding to the analyzed cells, was acquired and analyzed as described before [9]. The analyses were performed with Incyte Software (Merck Millipore), while graphs were obtained through FCS Express Software (DeNovo Software) and Origin (OriginLab). Tests were performed in triplicates and ANOVA tests were performed with Origin (OriginLab).

## 3. Results and Discussion

### 3.1. Structural and Morphological Characterization

**Figure 1:**
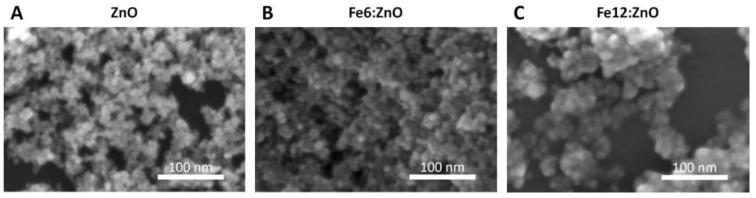
Field emission scanning electron microscopy images of ZnO (**A**), Fe6:ZnO (**B**) and Fe12:ZnO (**C**) nanoparticles having 0, 6 and 12 at.% of nominal iron doping, respectively.

The morphology of the ZnO and Fe:ZnO NPs was investigated by FESEM analysis. As shown in Figure 1, the resulting particles are almost spherical and present a dimension between 6 and 1 nm. No particular differences are found in the morphology and dimension between undoped and Fe-doped NPs, differently from what is typically reported in the literature [39], in which a reduction in the dimension because of doping is acknowledged. The maintenance of the size and morphology here reported may be attributed to a preponderance of the steric effects of the reagents exploited in our synthesis procedure, rather than a possible effect that doping may have on the rate of NP nucleation and crystal growth.

Another important point that arises from this analysis is the narrow size distribution of the nanoparticles. Independently of the level of doping, very uniform particle shapes and dimensions are observed. This aspect is further corroborated by DLS results discussed below.

EDS analyses were performed on Fe:ZnO nanoparticles to assess the actual level of iron incorporated in the nanoparticles (Figure S1). As it can be clearly observed from Table 1, by increasing the amount of the iron precursor during the synthesis, the atomic percentage of iron included in the NPs increases accordingly. In particular, the ratio between iron at.% and zinc at.% (Fe_at%_/Zn_at%_) is equal to 5.1% and 8.4% for the Fe6:ZnO and Fe12:ZnO NPs, respectively. These results demonstrate that iron has been successfully included in the NP systems and that the Fe doping level can be controlled by the synthetic procedure.

Among others, the high level of oxygen is instead attributed to the presence of oxygen-rich organic species in the system, i.e., the oleic acid used to stabilize the nanoparticles.

Furthermore, a source of error in the oxygen estimation in EDS is the sample preparation procedure, which in our case consists in spotting and letting dry the colloidal solution of low concentrated NPs on the sample holder instead of depositing a pellet and pressing it on the sample holder surface (as according to ISO 22309).

Fourier transform infrared spectroscopy was carried out to verify the presence of the oleic acid functional groups on the nanoparticles and to check the correct amino functionalization, required to label the nanoparticles for biological tests and to increase the stability of the nanoparticles.

**Figure 2:**
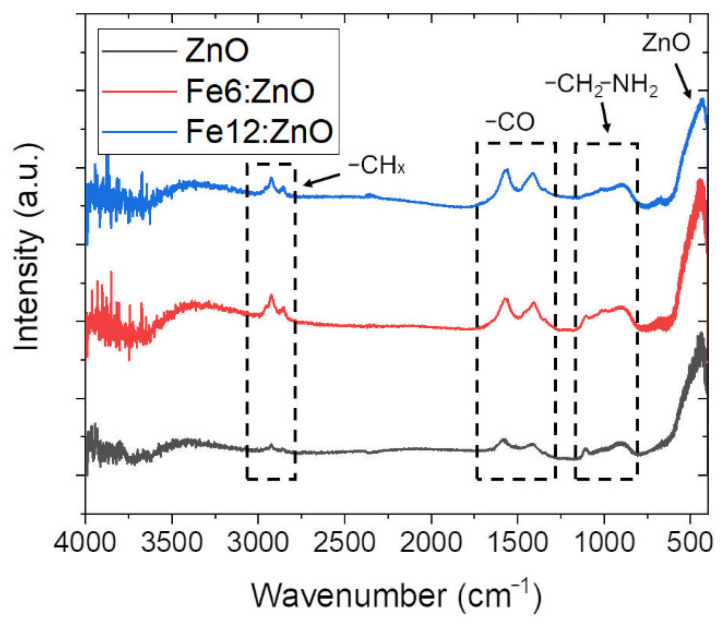
Fourier transform infrared spectroscopy spectra of ZnO, Fe6:ZnO and Fe12:ZnO NPs after amino-propyl functionalization.

In Figure 2, the spectra retrieved for the functionalized ZnO and Fe:ZnO NPs are reported. Despite the different doping levels, the Fe:ZnO NPs show several common features. First of all, there is an intense peak centered at ∼440 cm^−1^, which can be related to the vibration of Zn-O bonds. Moreover, two peaks at ∼1420 and ∼1570 cm^−1^ can be attributed to the C-O and C=O vibrations, respectively, while the peaks at ∼2860 and ∼2925 cm^−1^ are assigned to the -CH_3_ and -CH_2_ symmetric and asymmetric stretching vibrations. The presence of these peaks is expected because of the inclusion of oleic acid in the system, the amino-propyl functionalization and the use of zinc acetate in the synthesis procedure, which may result in some acetate residual. Finally, the broad band found between 3200 and 3600 cm^−1^ is ascribed to the OH stretching vibration. The abundance of hydroxyl groups, useful for promoting the anchoring of functional moieties on the NP’s outermost surface, is evident for both the amino-propyl-functionalized and unfunctionalized NPs (Figure S2), revealing how these NPs are prone to be functionalized without requiring any further surface activation steps.

The effectiveness of amine functionalization is corroborated by the presence of a small but sharp peak in the region between 1100 and 750 cm^−1^, which is attributed to primary aliphatic amines (-CH_2_-NH_2_) [42]. This peak is no longer visible in the IR spectra of the nonfunctionalized NPs (Figure S2).

**Figure 3:**
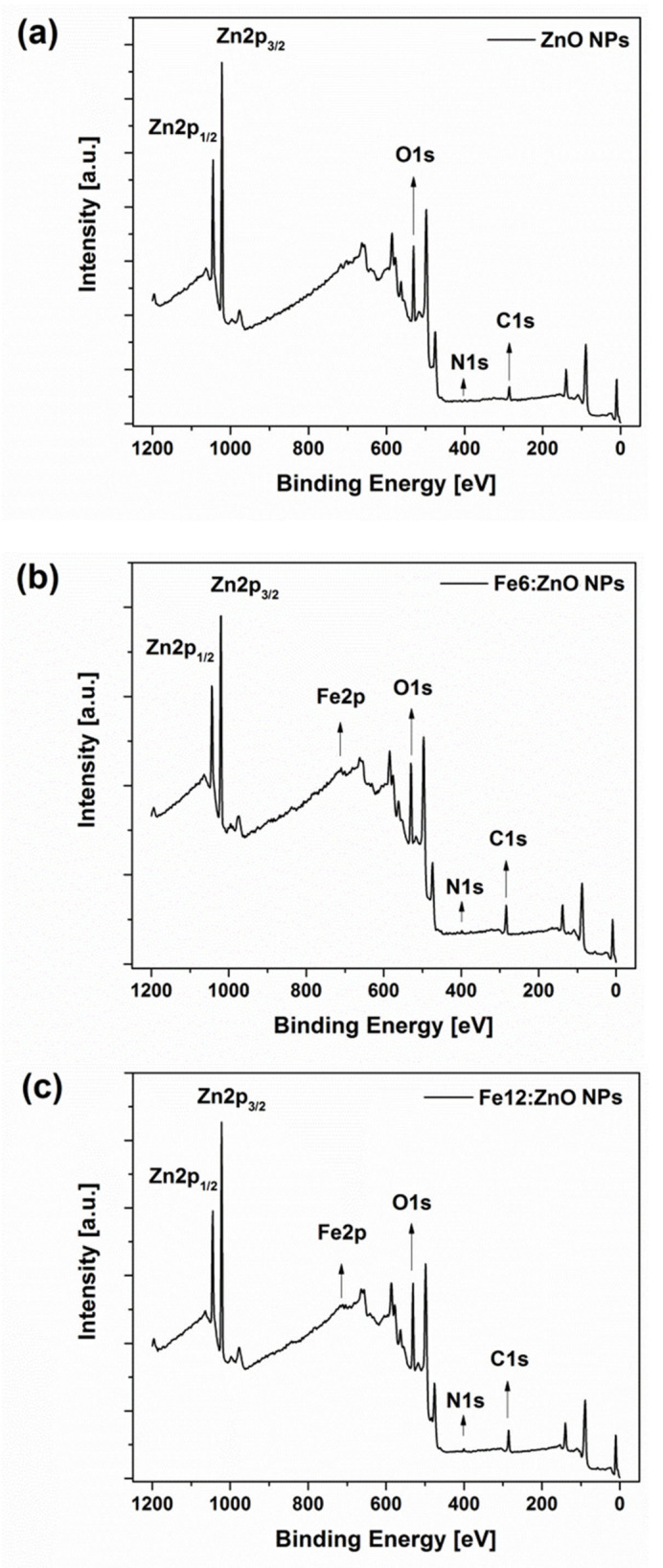
Wide energy range XPS spectra for undoped ZnO (**a**), Fe6:ZnO (**b**) and Fe12:ZnO (**c**) NPs.

Further insights about the composition of the prepared NPs were obtained by X-ray photoelectron spectroscopy (XPS). The wide energy range analysis for the undoped ZnO NPs revealed the presence of Zn, C, O and N (Figure 3a). Additionally, Fe element was detected only for the doped NPs (Figure 3b,c). The detection of N1s signal for all the NPs, i.e., the undoped and doped ones, confirms the successful amine functionalization. On the other side, the detection of Fe only for Fe:ZnO NPs was expected and highlights the success of our synthetic method for incorporating Fe dopant within ZnO NPs.

The relative atomic concentration (at.%) of each chemical element (Table 2) was estimated from the corresponding high-resolution (HR) spectra shown in Figures S3–S5 of the Supplementary Materials. The high presence of carbon is associated with the use of oleic acid in the synthesis process and with some contamination due to air exposure of the samples. Despite the amount of iron incorporated in the ZnO NPs being lower than the EDS data of Table 1, the Fe_at%_/Zn_at%_ ratio estimated from XPS results is similar and equal to 6.0% and 9.0% for Fe6:ZnO and Fe12:ZnO NPs, respectively.

**Figure 4:**
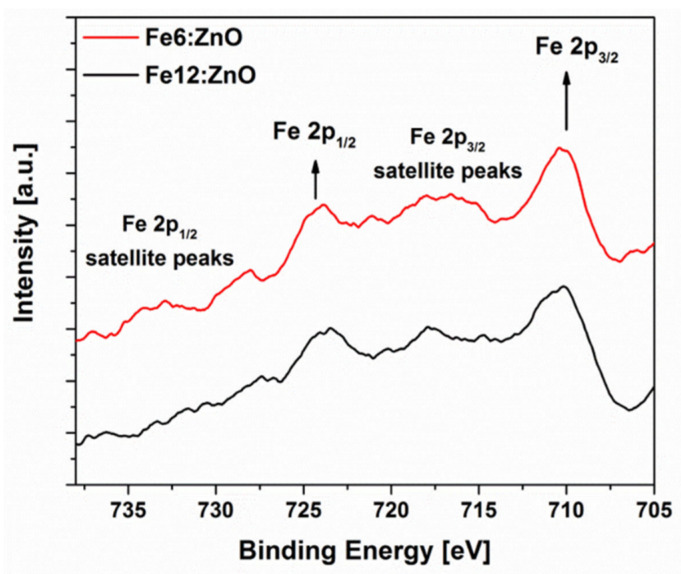
High-resolution XPS spectra of Fe2p for Fe:ZnO NPs doped at different Fe concentrations.

The local chemical environment of Fe dopant was further analyzed by collecting the high-resolution (HR) XPS spectrum of Fe2p, which is shown in Figure 4. The presence of multiple peak splits of Fe2p into separate components Fe2p_3/2_ and Fe2p_1/2_ is observed, which are centered at about 711 and 723 eV, respectively. Additional satellites in the 715–719 eV and 730–735 eV regions are also present and suggest the existence of multiple Fe oxidation states.

**Figure 5:**
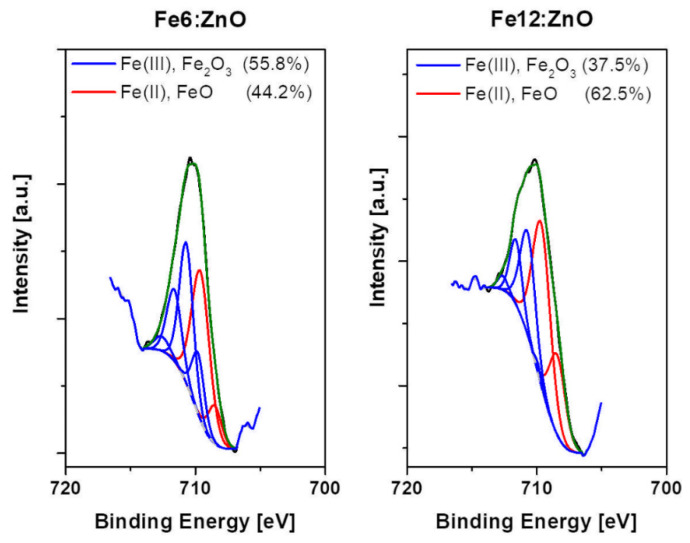
Curve-fitted Fe2p_3/2_ spectrum for Fe:ZnO nanoparticles.

HR Fe2p_3/2_ spectra deconvolution is shown in Figure 5. Each spectrum could be fitted with separate components associated with Fe-O bonds involved in FeO and Fe_2_O_3_, respectively [43]. This aspect evidences the coexistence of Fe^3+^ and Fe^2+^ valence states for Fe dopant in both the doped samples. If the area percentage under each component is considered (Table S1 of the Supplementary Materials), a preponderance of Fe^3+^ ions relative to Fe^2+^ can be highlighted for the sample Fe6:ZnO. On the contrary, Fe^2+^ ions are predominant for sample Fe12:ZnO. By increasing the amount of Fe dopant, an increase in Fe^2+^ fraction at the expense of Fe^3+^ one is then observed, similarly to what was reported in other works [44,45,46].

**Figure 6:**
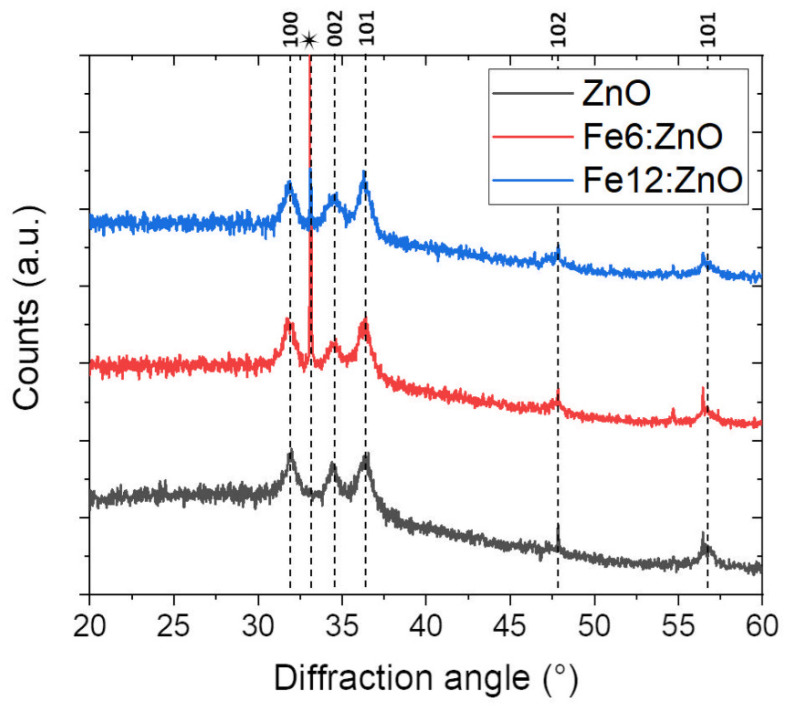
X-ray diffraction patterns of ZnO, Fe6:ZnO and Fe12:ZnO NPs. * refers to the silicon wafer used as substrate.

The crystallinity of the ZnO and Fe:ZnO nanoparticles was evaluated through X-ray diffraction. The resulting patterns are reported in Figure 6. Despite the broadening due to the small dimensions of the particles, the peaks belonging to the wurtzitic structure of ZnO can be identified in all three cases according to JCPDS-ICDD (card No. 89-1397) and show that all the prepared NPs are crystalline. In particular, the most intense ones can be assigned to (100), (002) and (101) ZnO crystallographic directions.

No additional diffraction peaks belonging to metallic Fe or Fe oxide phases are observed within the detection limit of the XRD instrument, suggesting that additional Fe-related crystalline phases neither nucleate and grow during the synthesis nor are detrimental for the crystalline wurtzitic structure of the nanoparticle.

As further information about the level of crystallinity, the dimension of the crystallites forming the nanoparticles has been evaluated according to the Debye–Scherrer formula. The results are summarized in Table 3 for the functionalized NPs. The crystallite dimension agrees with FESEM results, which showed that the dimension of the particles ranged between 6 and 10 nm. This allows us to state that our nanoparticles are single ZnO nanocrystals, for both the undoped and doped cases. Moreover, the crystallite dimension is even increased with doping, indicating that the introduction of Fe ions may induce a slight increase in the crystalline structure of the nanoparticle.

The introduction of doping atoms in the host ZnO crystal is indeed expected to induce a distortion in the lattice structure, which can also be evaluated by considering the shift of the 2θ peak positions measured by the XRD pattern.

In Table 4, the shift measured by comparing the 2θ angles corresponding to the three main reflections of ZnO and Fe:ZnO NPs is reported. The main variations are along the (001) and the (002) planes and suggest a slight modification of the lattice parameters due to the difference in ionic radii between Zn^2+^ (0.74 Å) and Fe^2+^/Fe^3+^ dopant (0.78/0.64 Å, respectively).

In conclusion, both XRD and XPS results state that Fe was successfully included in the host ZnO lattice structure. This aspect is of particular interest especially in view of the magnetic and electromechanical behavior of the doped NPs, which are discussed in the next sections.

The functionalized NPs were analyzed through dynamic light scattering (DLS) in two different solvents: ethanol and water.

Table 5 summarizes the results for the amino-functionalized particles (whose graphs are reported in Figure S6). The polydispersity index (PDI) in both water and ethanol never exceeds the 0.160 value and confirms the narrow size distribution already highlighted with FESEM analysis. Therefore, it can be stated that the population of the considered NPs is substantially monodisperse [24].

It must be observed that the hydrodynamic diameter found from DLS measurements is well above the dimensions found with FESEM analysis. In fact, the smallest value is found for undoped ZnO (close to 100 nm, Table 5) and is 10 times higher than the NP size estimated by electron microscopy (Figure 1). This phenomenon can be attributed both to the presence of chemical groups on the particle surface which increase the hydrodynamic diameter of the particle and to the adsorption of ions at the NP surface or even to the formation of NP aggregates.

The hydrodynamic size of the nanoparticles is increased when the doping level is increased as well, suggesting a higher level of aggregation of the doped nanoparticles.

However, the level of monodispersion is still high also in water, where the hydrodynamic diameter is the largest one. This is due also to the surface charge of the nanoparticles in colloidal solution which is highly positive, well above +20 mV (Table 6), and in agreement with what was found for ZnO NPs after amino functionalization because of the presence of the amino groups [40]. However, the overall result is to have highly positively charged nanoparticles that repel each other, assuring an optimal dispersion.

### 3.2. Optical, Magnetic and Piezoelectric Characterization

**Figure 7:**
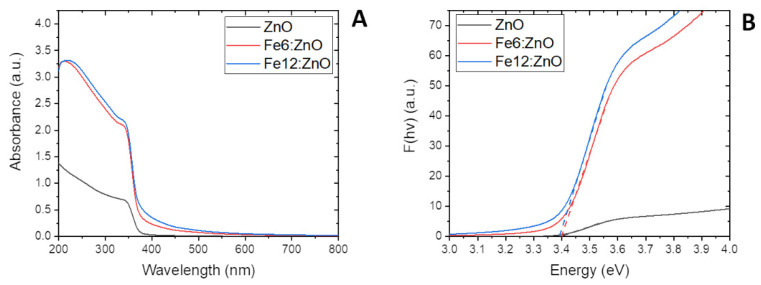
UV–visible spectra (**A**) and Tauc’s plot (**B**) of ZnO and Fe:ZnO NPs.

The absorption spectra recorded for ZnO and Fe:ZnO NPs are shown in Figure 7A. From the optical standpoint, a strong absorption in the ultraviolet (UV) region is observed for all three samples, as represented by the absorption peak at λ = 348 nm. The UV absorption intensity is more pronounced for Fe:ZnO NPs and in particular for the most doped ones (Fe12:ZnO NPs). All these aspects make the prepared NPs good UV-absorber materials and promising candidates for many applications in which a good UV absorption is required, such as photodynamic therapy [47,48] or antimicrobial purposes [49,50].

Moreover, it is worth mentioning that Fe:ZnO nanoparticles present a slight absorption in the visible region, which is further intensified as the level of doping is increased. This aspect may play a significant role in biomedical applications, since extending the optical activation of the NPs, e.g., for photodynamic therapies, can be beneficial when using lower-energy and thus safer light sources.

From the UV-vis spectra, the optical bandgap of the NPs could be estimated according to Tauc’s plot [51], as shown in Figure 7B. The calculated bandgap value is around 3.39 eV for all the samples. This value is in agreement with what was previously found by some of us for Gd- and Mn-doped ZnO NPs prepared with a similar synthetic technique, where very small bandgap variations among undoped and doped ZnO NPs were found [35].

Doping ZnO typically involves a modification of the electronic structure of the material by the generation of new electronic states. If these electronic states fall in the bandgap region, new electronic transitions upon light excitation are allowed, resulting in fluorescence emission [52].

For this reason, the fluorescence spectra of the synthesized Fe:ZnO NPs were acquired.

**Figure 8:**
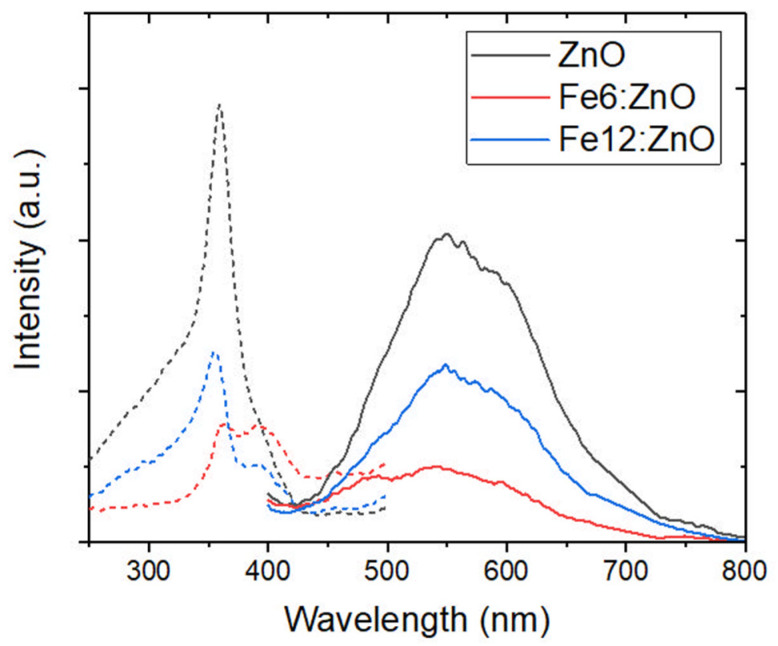
Fluorescence spectra of ZnO and Fe:ZnO NPs. Dashed lines represent the signal retrieved when exciting the sample at wavelength below λ = 500 nm and collecting the emission at λ = 550 nm; solid lines refer to the emission retrieved by exciting the sample at λ = 348 nm.

The results are reported in Figure 8 for all three kinds of nanoparticles. When excited with UV radiation (here in particular with λ = 348 nm), all the NPs show a broad green light emission (centered at λ = 550 nm, see all the solid lines), in agreement with what was found for other ZnO nanostructures [53,54]. However, this is typically attributed to oxygen vacancies and related effects rather than to doping [55], for which there is no rising of any additional peak. Moreover, the fluorescent emission of the doped nanoparticles is lower than that of the undoped ones. However, when considering the excitation spectra (dotted lines in Figure 8), both the iron-doped NPs present a secondary peak of excitation at 400 nm. The presence of this peak is in agreement with what was found in the UV-vis spectra where a higher absorption was observed up to λ = 400 nm. This feature confirms that the prepared iron-doped NPs are good UV-absorber materials, with potential use for photodynamic therapy or antimicrobial applications, as highlighted above.

**Figure 9:**
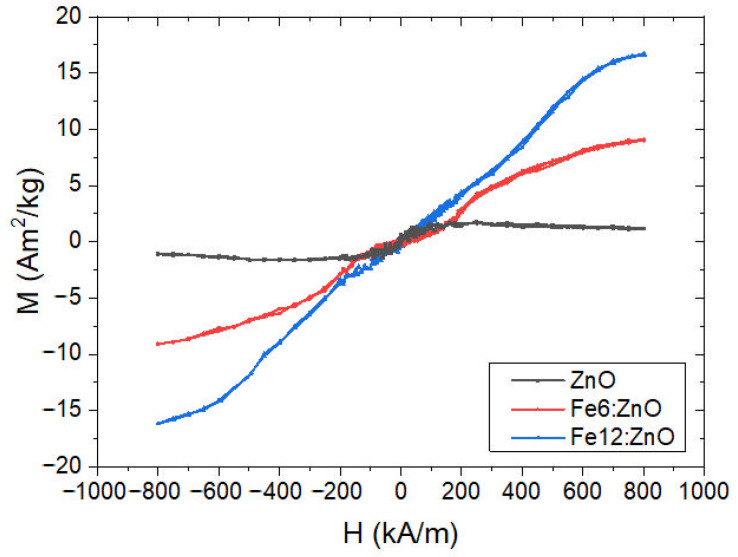
Magnetization–saturation (M-H) curves of ZnO and Fe:ZnO NPs with different doping levels, measured at room temperature.

Introducing magnetic ions inside a semiconductor may allow the formation of localized magnetic dipoles that can affect the electronic carriers’ behavior [38]. This is the case of iron ions, which present an incomplete d electronic shell. When included in the ZnO crystal structure, the appearance of a ferromagnetic behavior is typically observed [56,57]. Moreover, the inclusion of iron ions can also induce the increase in the maximum magnetization that the particles can reach at room temperature under the application of a magnetic field. Figure 9 reports the measurements regarding the DC magnetization of ZnO and Fe:ZnO NPs. As it can be noticed, all the nanoparticles show a paramagnetic behavior, which is, however, modified in magnitude by increasing the Fe doping. Interestingly, a similar paramagnetic response is found also for the undoped ZnO nanoparticles, for which a diamagnetic behavior was expected. This behavior may be explained by the presence of the oleic acid shielding, which could partially influence the surface electron behavior and change the magnetic properties of the nanoparticle, as it happened in other ZnO-based systems with different functionalizations [58]. For this purpose, this paramagnetic response for undoped nanoparticles has been found by some of our group [35] as well, with an almost identical magnetization magnitude.

As already mentioned, increasing the amount of doping enhances the maximum magnetization measured when applying a magnetic field of 800 kA/m on the sample. In fact, for the Fe12:ZnO NPs, a magnetization of 16.7 Am^2^/kg is found, against the 9.1 Am^2^/kg retrieved in the case of Fe6:ZnO NPs. These values are almost one order of magnitude higher with respect to the maximum magnetization obtained for the undoped nanoparticles (1.5 Am^2^/kg).

The origin of magnetic properties in Fe-doped ZnO materials is still under debate. Several studies pointed out that they may originate from the formation/precipitation of secondary magnetic phases within the material [59]. Instead, other works showed that the magnetic behavior of Fe:ZnO NPs is due to the existence of mixed Fe valence states allowing the exchange interaction between conductive electrons and local spin-polarized electrons [60,61,62,63].

The noticeable enhancement in the maximum magnetization observed for our Fe:ZnO NPs has to be attributed to the effective incorporation of Fe magnetic atoms in the ZnO crystal structure. XPS and XRD results allow the exclusion of the formation/precipitation of secondary magnetic phases and point out the coexistence of both Fe^2+^ and Fe^3+^ ions substituting at Zn^2+^ sites. It is also noticed that the dominant Fe valence state (Fe^2+^ or Fe^3+^) changes according to the overall dopant amount. Therefore, the magnetic behavior observed in our Fe:ZnO NPs is more likely due to the electron double-exchange mechanism [63,64,65], while the differences in magnetic behavior among the Fe-doped NPs can be due to the difference in the amount of incorporated Fe dopant and to the change of the dominant Fe valence state [60,61,62,63]. This strong paramagnetic behavior of the nanoparticles can be then successfully exploited for biomedical imaging applications, such as magnetic resonance imaging.

**Figure 10:**
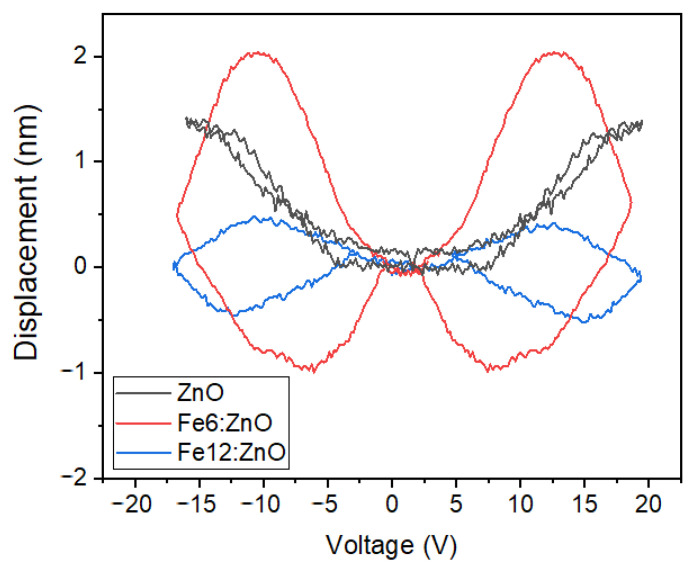
Electromechanical displacement vs. applied bias voltage, measured for ZnO and Fe:ZnO NPs with different doping levels.

The electromechanical behavior of Fe:ZnO NPs was qualitatively investigated by considering the mechanical displacement experienced by the nanoparticle film under the application of an external bias voltage (Figure 10). It is found that Fe doping influences the electromechanical response, with Fe:ZnO NPs showing closed-loop curves resembling those reported in the literature for common piezoelectric ceramics (PZT, barium titanate) [66,67] and other doped ZnO materials [29,68]. In particular, Fe6:ZnO NPs show a better electromechanical response than Fe12:ZnO, with an improved peak-to-peak mechanical displacement that changes from around 1 nm for sample Fe12:ZnO to around 3 nm for sample Fe6:ZnO. Despite showing an electromechanical displacement comparable to Fe6:ZnO NPs, the behavior of undoped ZnO NPs is slightly different; the D-V curve shows a negligible hysteretic behavior, suggesting the absence of a ferroelectric domain structure, in accordance with the literature [69]. It is worth mentioning that the remarkable mechanical displacements measured in this work could also be biased by the bending of the substrate, which cannot be neglected with the used measurement apparatus [70]. Therefore, a quantitative estimation of the piezoelectric constant for the tested NPs cannot be obtained, as it would be overestimated due to the above-mentioned effect superimposed on the piezoelectric one.

The improved electromechanical response due to Fe doping can be related to the different valence states of the Fe ions incorporated in the ZnO crystal. Both XPS and XRD analyses pointed out that Fe dopant was correctly incorporated in the host ZnO crystal structure, with Fe^3+^/Fe^2+^ ions substituting at Zn^2+^ sites. The fraction of Fe^3+^/Fe^2+^ ions is changed according to the dopant amount. In particular, the smallest Fe^3+^ is the dominant valence in sample Fe6:ZnO. By increasing the nominal doping level up to 12 at.%, the biggest Fe^2+^ ions become the predominant ones. Among others, the electromechanical response of wurtzitic ZnO is governed by the ease rotation/bending of bonds in the crystalline cell. The substitution of Zn^2+^ sites with smaller Fe^3+^, having also a higher positive charge, promotes an easy bending/rotation of bonds under the application of an external electric field, making easier the alignment of electric dipoles along the *c*-axis direction, i.e., the polarization direction of ZnO, and finally improving the overall piezoelectric mechanical displacement of the material [46]. On the other hand, the substitution of Zn^2+^ with bigger Fe^2+^ ions makes this rotation/bending more difficult. Fe^2+^ is the predominant valence for Fe dopant in the sample Fe12:ZnO. Therefore, in this case, it is found that Fe doping could somehow limit the electromechanical response. Finally, it can also be concluded that a proper modulation of the chemical state for Fe dopant must be foreseen if an enhancement in the electromechanical response of Fe:ZnO NPs is to be pursued. Furthermore, the enhanced electromechanical response provided by iron doping could represent an interesting tool for therapeutic purposes, because the possibility to induce a tunable electric potential on the treated cells may also disrupt cell ion homeostasis and induce anticancer drug sensitization [21].

### 3.3. Biological Characterization

**Figure 11:**
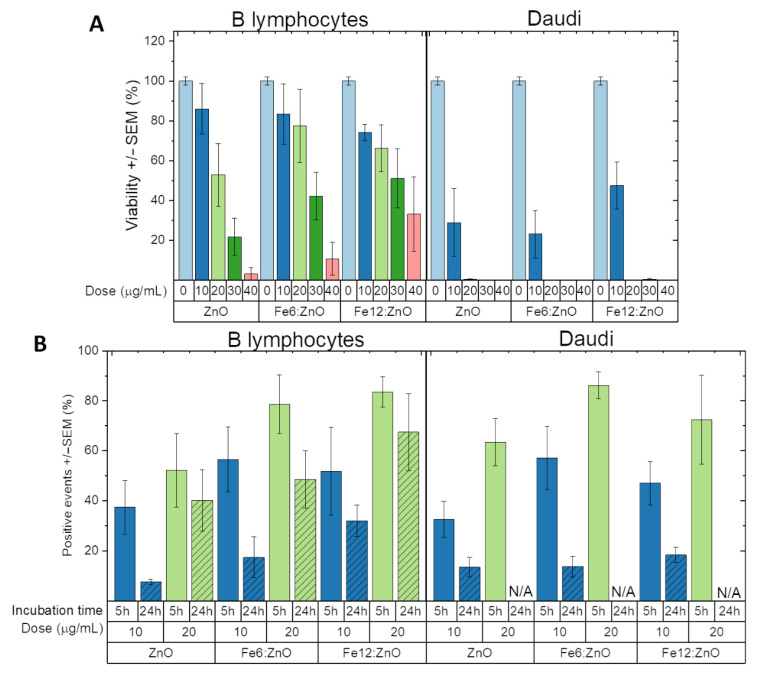
(**A**) B lymphocyte and Daudi cell viability evaluated through the WST-1 assay at 24 h after ZnO and Fe:ZnO NPs administration. (**B**) B lymphocyte and Daudi cell Fe:ZnO NP uptake evaluated through cytofluorimetry at 5 and 24 h after NP administration to cells. N/A denotes experiments for which the high NP toxicity resulted in the number of events presenting insufficient values for the evaluation of the NP uptake. Error bars express standard error mean.

ZnO and Fe:ZnO nanoparticles were firstly tested on representative examples of both normal and cancerous hematic cells, i.e., B lymphocytes and Burkitt’s lymphoma Daudi cell lines. Figure 11A reports the results obtained in terms of cell viability expressed as viability percentage with respect to an untreated sample (i.e., 0 μg/mL dose of nanoparticles). The complete statistical analysis is reported in the Supplementary Materials.

All the analyzed NPs are not toxic for B lymphocytes up to 10 μg/mL, while dose-dependent toxicity is observed with the increase in the concentration. Moreover, iron doping seems to slightly reduce the toxicity of ZnO NPs at higher concentrations. The reason for the reduced toxicity is attributed to the inhibition of nanoparticle dissolution because of the inclusion of iron ions inside the ZnO crystal lattice and the consequent lower amount of cytotoxic zinc cations released inside the cell [39].

Interestingly, all the analyzed NPs were shown to be more toxic for cancer cells than for healthy cells. Indeed, as shown in Figure 11A, Daudi cells start to suffer from the presence of ZnO and iron-doped ZnO NPs already at a concentration of 10 μg/mL, and no metabolic activity was observed through WST-1 assay at higher concentrations. The selective toxicity of pure ZnO NPs toward cancer cells has been already found in other works [14,71,72]. The toxicity mechanism seems to be mediated by different pathways having as main triggering factors the dissolution of the nanoparticles in toxic zinc cations and the generation of reactive oxygen species. Anyway, the iron-doped NPs considered in this work confirm this selectivity. Furthermore, the addition of imaging potentialities thanks to enhanced magnetic response suggests their use as a powerful theranostic platform.

The NP uptake in hematic cells was also studied through cytofluorimetry. In particular, 10 and 20 μg/mL NP doses were administered to both B lymphocytes and Daudi cells, and the cells were analyzed after 5 and 24 h of incubation. The results (Figure 11B) show that for healthy B lymphocyte cells, the higher the dose, the higher the percentage of positive events, indicating that more cells are able to internalize NPs. This phenomenon can be very likely attributed to the higher availability of the NPs for the cells. Moreover, the uptake at 5 h of incubation is generally higher than that after 24 h, suggesting that a high internalization of the NPs in the first hours after administration is followed either by a NP release phase or by the dissolution of the particles themselves. Similar results are found for the Daudi cell line (Figure 11B), where it is worth noting a high internalization rate of all the NP types at 20 µg/mL after the first 5 h of incubation. As in Figure 11A, the cytotoxicity at 24 h is reported; this NP uptake result supports the idea that NP toxicity towards Daudi cells is not related to the large number of cells able to internalize the nanosized particles. Instead, we can suppose that this toxicity behavior is related to an improved sensitivity of the tumoral cell line, with respect to the healthy one, toward the intrinsic toxicity mechanism of the ZnO nanoparticles.

As a final remark, iron doping seems to slightly increase the ability of NPs to be internalized by both the cell lines, with the highest uptake values obtained for 20 μg/mL of NPs after 5 h of incubation (78.63% and 86.19% positive events for B lymphocytes and Daudi cells, respectively).

It is clear that for hematic cell lines the proposed Fe-doped ZnO nanoparticles can be easily adopted as an inorganic nanodrug against cancer cells. Actually, a clear therapeutic window is found, showing that concentrations up to 20 µg/mL of both Fe6- and Fe12-doped ZnO are nontoxic for healthy cells while inducing death in tumoral ones. However, as a possible drawback, the low concentration at which the theranostic platform is administered to tumor cells does not probably allow on-demand triggering of further therapeutic phenomena mediated by the increased piezoelectric response. Moreover, the low internalization at longer incubation time may represent a limitation for long-term monitoring of the cells or even animal conditions (for future in vivo tests) through the novel imaging potentialities given by the enhanced magnetic behavior.

For these reasons, ZnO, Fe6:ZnO and Fe12:ZnO NPs were also tested on another cell line, i.e., BxPC-3, in order to show how a different system, i.e., adherent cells from a dramatically challenging solid tumor, pancreatic ductal adenocarcinoma, may react to the nanoparticles.

**Figure 12:**
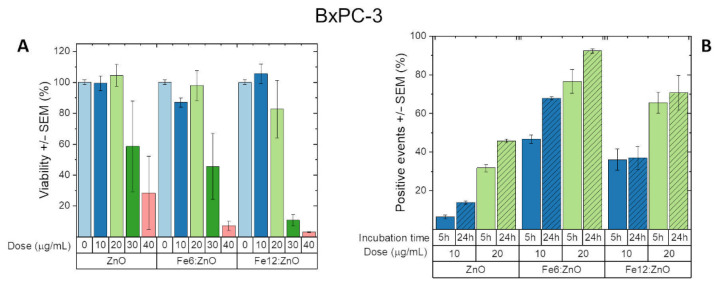
(**A**) BxPC-3 cell viability evaluated through the WST-1 assay at 24 h from ZnO and Fe:ZnO NPs administration. (**B**) BxPC-3 cell Fe:ZnO NP uptake evaluated through cytofluorimetry at 5 and 24 h from NP administration to cells. Error bars express standard error mean.

The results of cytotoxicity and uptake experiments are reported in Figure 12A,B, respectively. These pancreatic tumoral cells are able to bear the presence of all the analyzed NPs up to 20 μg/mL, regardless of the iron doping level. At higher doses, the NPs exhibit toxicity that is more and more accentuated with the increase in NP concentration. Moreover, a very slight increase in toxicity is also found with the increase in doping at high NP doses. This trend is opposite of what has been found for hematic cells, although the increase in toxicity induced with doping is very low as the doping level is low as well. Further studies will be surely required to elucidate this different behavior. The use of the proposed iron-doped NPs in place of the undoped ones is anyway justified by the newly acquired magnetic and piezoelectric properties. Moreover, as these cells are more resistant to the ZnO and iron-doped ZnO NPs, there is a wider range of concentrations that can be administered to pancreatic cancer cells. This suggests the use of these NPs as a platform that can be externally or remotely stimulated to induce toxicity on demand.

This possibility is further corroborated by the extremely high levels of uptake that are obtained in BxPC-3 with iron-doped ZnO nanoparticles. Indeed, as shown in Figure 12B, the percentage of positive events can reach values exceeding 90% for Fe6:ZnO 24 h after particles administration. The results reported in Figure 12B clearly demonstrate that iron-doped NPs are more easily internalized by the BxPC-3 cell lines, with a maximum of 92.33% positive events reached with 20 μg/mL dose of the intermediate level of doping NPs (Fe6:ZnO). Furthermore, while the ZnO and Fe6:ZnO NPs both show an increase in the positive events with the incubation time, the Fe12:ZnO ones seem to have already reached their plateau at 5 h of incubation, with no significant increase after 24 h of incubation. This trend suggests that the differences found between the three nanoparticles in terms of physical and chemical properties not only slightly influence their toxicity but also influence their behavior toward BxPC-3 cells, even if further studies focused on the internalization mechanism are surely required. The very high level of internalization in BxPC-3, together with its enhanced magnetic properties and superior piezoelectric response, places Fe6:ZnO as the best nanoparticle among those investigated in this work, with enormous potentialities in terms of smart therapies for pancreatic cancer treatment.

## 4. Conclusions

In this work, iron-doped ZnO NPs were developed to obtain a versatile theranostic material to be used in cancer therapies.

NPs with a diameter between 6 and 10 nm were synthesized with different levels of iron doping, and pure ZnO nanoparticles were used as control. The spherical morphology of the nanoparticles was not affected by doping. EDS and XPS analyses revealed that iron doping incorporation within the NPs was consistent with the synthesis protocol. XRD and XPS analyses showed that NPs were single-phase wurtzitic crystals and confirmed the correct inclusion of iron ions in the host ZnO crystal lattice. With the increase in doping, Fe^2+^ was found to be the predominant oxidation state, despite both Fe^2+^ and Fe^3+^ ones being present in all the doped particles.

The optical behavior of the NPs changed with doping. Indeed, despite all the ZnO NPs still being strong UV absorbers, the inclusion of iron in the NPs enlarged the light absorption spectral range of the system toward visible light, paving the way to biomedical applications, i.e., therapeutic activation by light as in photodynamic therapy.

Both ZnO and Fe:ZnO NPs showed a paramagnetic behavior. However, the inclusion of iron magnetic moments inside the crystal elicits a noticeable enhancement of the maximum magnetization, with consequent potential uses of Fe:ZnO NPs as contrast agents in magnetic resonance imaging.

An increased electromechanical response was found for the doped NPs. The presence of a hysteresis in the corresponding D-V curve is attributed to the distortion that the iron ions may induce in the ZnO crystal, while the difference in the electromechanical response between the doped NPs is attributed to the different ratio of Fe^2+^ and Fe^3+^ species, which may lead to a different distortion of the lattice because of ionic radii. The enhanced electromechanical response suggests the use of the iron-doped nanoparticles also as a therapeutic agent, where the electric potential generated remotely by an external mechanical stimulation may alter specific cell behaviors to the point of promoting healthy cell proliferation or even inducing tumoral cell death.

Finally, ZnO and Fe:ZnO NPs were evaluated in terms of cytotoxicity and cellular uptake in vitro with healthy and cancerous blood cells and with a pancreatic ductal adenocarcinoma cell line. In blood cells, a high selectivity of all the analyzed nanoparticles toward tumoral cells has been found, with a clear concentration window in which cancerous cells are killed while healthy cells remain unharmed. Instead, pancreatic tumoral cells, independently of the considered NP, showed their safety up to 20 μg/mL. At higher doses, Fe12:ZnO NPs were proved to be more toxic than ZnO and Fe6:ZnO NPs, which present comparable levels of biocompatibility. Interestingly, Fe:6ZnO NPs showed a higher affinity in being internalized by BxPC-3 cells during uptake experiments when compared to the other NPs, with more than 90% of cells having internalized NPs after 24 h.

In conclusion, Fe:ZnO NPs showed very promising optical, magnetic and electromechanical properties that could be effectively used in nanomedicine. In particular, Fe6:ZnO NPs showed the best trade-off between enhanced functionalities and cellular compatibility, suggesting their use as a powerful theranostic agent.

## Figures and Tables

**Table 1:** Energy-dispersive spectroscopy (EDS) results of Fe:ZnO nanoparticles. Here, only the results related to the Zn, Fe and O elements are considered.

Sample	Zn (at.%)	Fe (at.%)	O (at.%)
ZnO	18.65	-	81.35
Fe6:ZnO	23.68	1.20	74.82
Fe12:ZnO	22.70	1.90	75.4

**Table 2:** Relative atomic concentration for ZnO and Fe:ZnO nanoparticles estimated from HR-XPS analysis.

Sample	C (at.%)	Zn (at.%)	O (at.%)	N (at.%)	Fe (at.%)
ZnO	14.8 ± 0.5	39.3 ± 0.4	43.7 ± 0.4	2.2 ± 0.4	-
Fe6:ZnO	24.3 ± 0.5	29.8 ± 0.3	41.8 ± 0.4	2.3 ± 0.3	1.8 ± 0.3
Fe12:ZnO	28.1 ± 0.6	27.8 ± 0.3	38.8 ± 0.4	2.8 ± 0.4	2.5 ± 0.4

**Table 3:** Crystallite dimension evaluated through the Debye–Scherrer equation for the (100) plane for the ZnO and Fe:ZnO NPs.

Sample	(100)
ZnO	6.0 nm
Fe6:ZnO	8.3 nm
Fe12:ZnO	12 nm

**Table 4:** Diffraction angle shifts on the peaks corresponding to the (100), (002) and (101) planes with respect to the ZnO NPs. Δθ = θ_Fe:ZnO_ − θ_ZnO_.

Sample	ΔΘ (100)	ΔΘ (002)	ΔΘ (101)
Fe6:ZnO	−0.121°	+0.061°	+0.020°
Fe12:ZnO	−0.088°	+0.114°	−0.032°

**Table 5:** Dynamic light scattering results for Fe:ZnO NPs. The hydrodynamic diameter (D_H_) and the polydispersity index (PDI) are expressed as mean values ± standard deviation of three measurements.

Sample	D_H_ in Ethanol	PDI in Ethanol	D_H_ in Water	PDI In Water
ZnO	98.5 ± 0.2 nm	0.154 ± 0.019	116.2 ± 0.4 nm	0.148 ± 0.011
Fe6:ZnO	120.4 ± 0.2 nm	0.133 ± 0.010	139.0 ± 1.8 nm	0.138 ± 0.025
Fe12:ZnO	124.4 ± 0.4 nm	0.144 ± 0.021	167.6 ± 1.7 nm	0.121 ± 0.003

**Table 6:** Z-potential of Fe:ZnO NPs with amino functionalization. The Z-potentials are expressed as mean values ± standard deviation of three measurements.

Sample	Z-Potential after Functionalization
0 at.%	22.7 ± 0.9 mV
6 at.%	26.4 ± 0.4 mV
12 at.%	25.5 ± 0.5 mV

## Data Availability

Not applicable.

## Supplementary Materials

The following are available online at https://www.mdpi.com/article/10.3390/nano11102628/s1, Figure S1: EDS spectroscopy spectra of undoped and iron-doped nanoparticles, Figure S2: FTIR spectroscopy spectra of undoped iron-doped nanoparticles before amino-propyl functionalization, Figures S3–S5: High-resolution XPS spectra of undoped and iron-doped nanoparticles, Table S1: Peak components of undoped and iron-doped nanoparticles Figure S6: Dynamic light scattering results of undoped and iron-doped nanoparticles in ethanol and water, statistical analysis of biological experimental results.
