# Supplementary material for: Iron-Doped ZnO Nanoparticles as Multifunctional Nanoplatforms for Theranostics
Source: Nanomaterials (Basel). 2021 Oct 6;11(10):2628. doi: 10.3390/nano11102628 (PMC8540240; doi:10.3390/nano11102628)
Supplement: Supplementary file 1 [file nanomaterials-11-02628-s001.zip › nanomaterials-1394649-supplementary.pdf]

Supporting Information

Iron-Doped ZnO Nanoparticles as Multifunctional Nanoplatforms for Theranostics

Marco Carofiglio, Marco Laurenti, Veronica Vighetto, Luisa Racca, Sugata Barui, Nadia Garino, Roberto Gerbaldo, Francesco Laviano and Valentina Cauda *

Department of Applied Science and Technology, Politecnico di Torino, C.so Duca degli Abruzzi 24,
10129 Turin, Italy; marco.carofiglio@polito.it (M.C.); marco.laurenti@polito.it (M.L.); veronica.vighetto@polito.it (V.V.); luisa.racca@polito.it (L.R.); sugata.barui@polito.it (S.B.); nadia.garino@polito.it (N.G.); roberto.gerbaldo@polito.it (R.G.); francesco.laviano@polito.it (F.L.)

* Correspondence: valentina.cauda@polito.it; Tel.: +39-011-090-7389

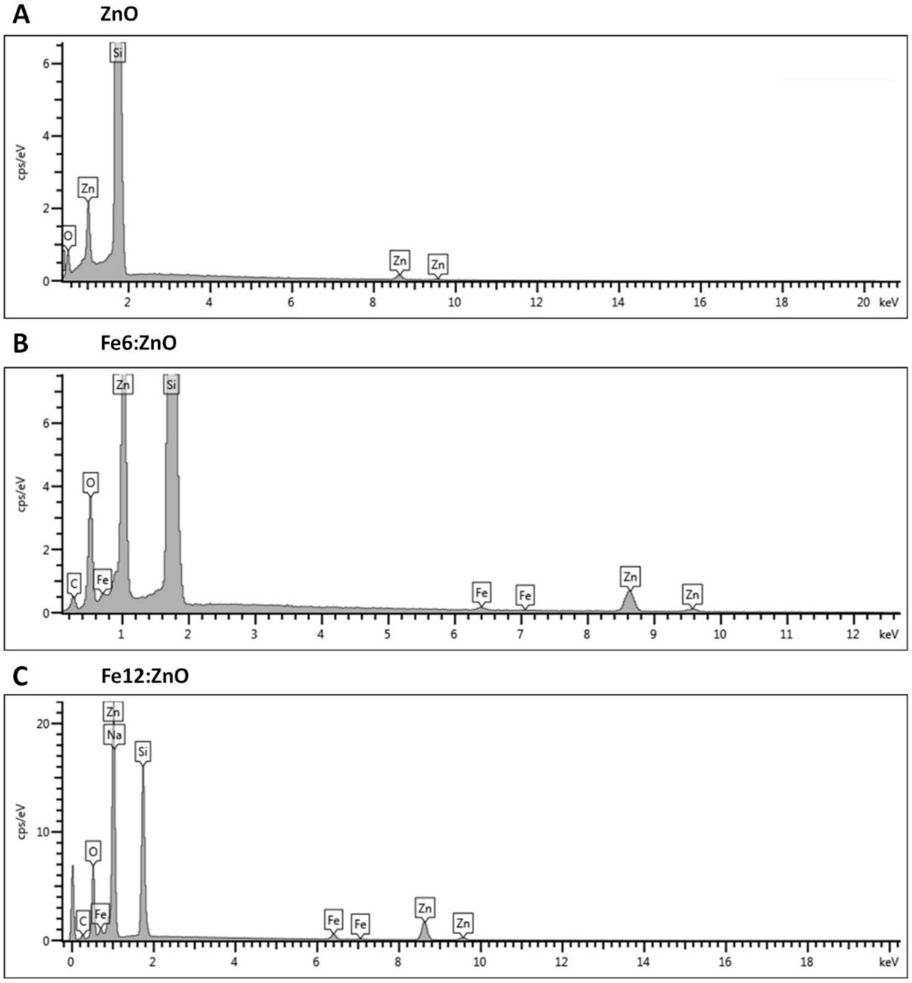

Figure S1. Electron dispersive energy spectroscopy spectra of 0 at.% (A), 6 at.% (B) and 12 at.% (C) iron-doped nanoparticles (Fe:ZnO).

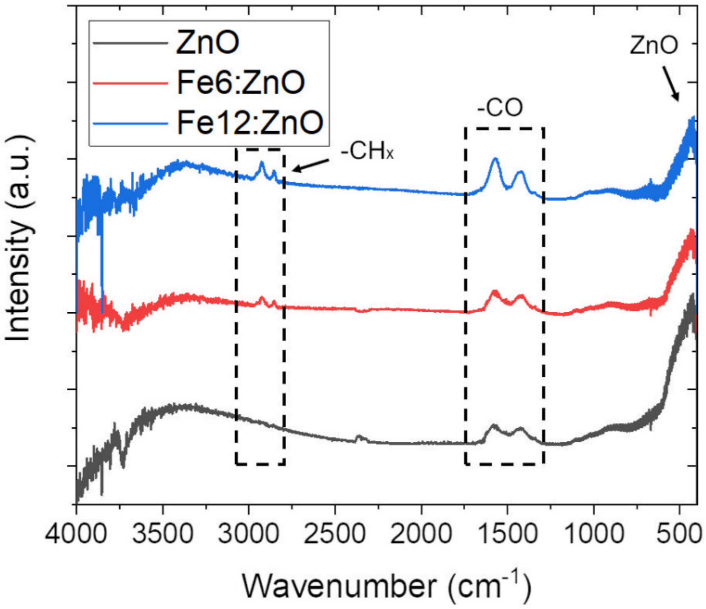

Figure S2. Fourier transform infrared spectroscopy spectra of ZnO and Fe:ZnO nanoparticles prior to amino-propyl functionalization.

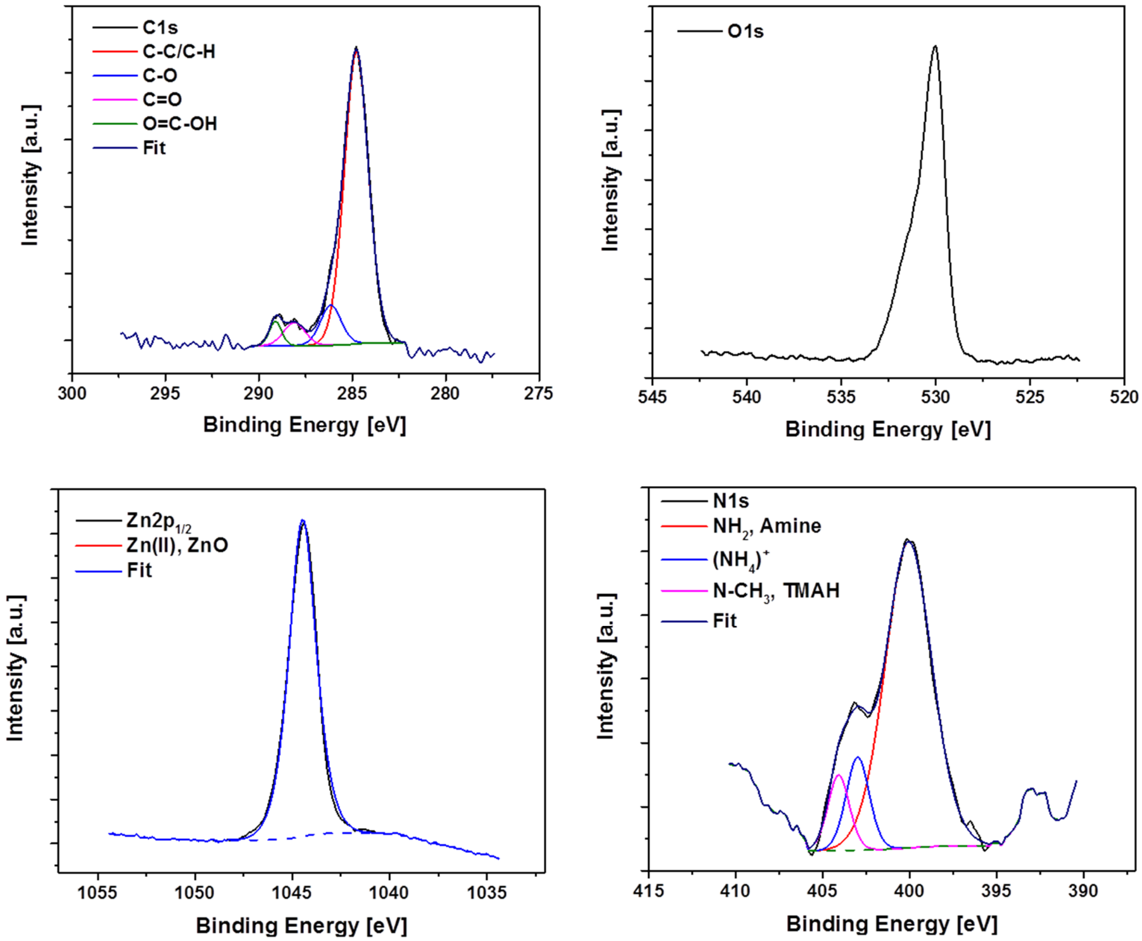

Figure S3. High-Resolution XPS spectra for undoped ZnO NPs.

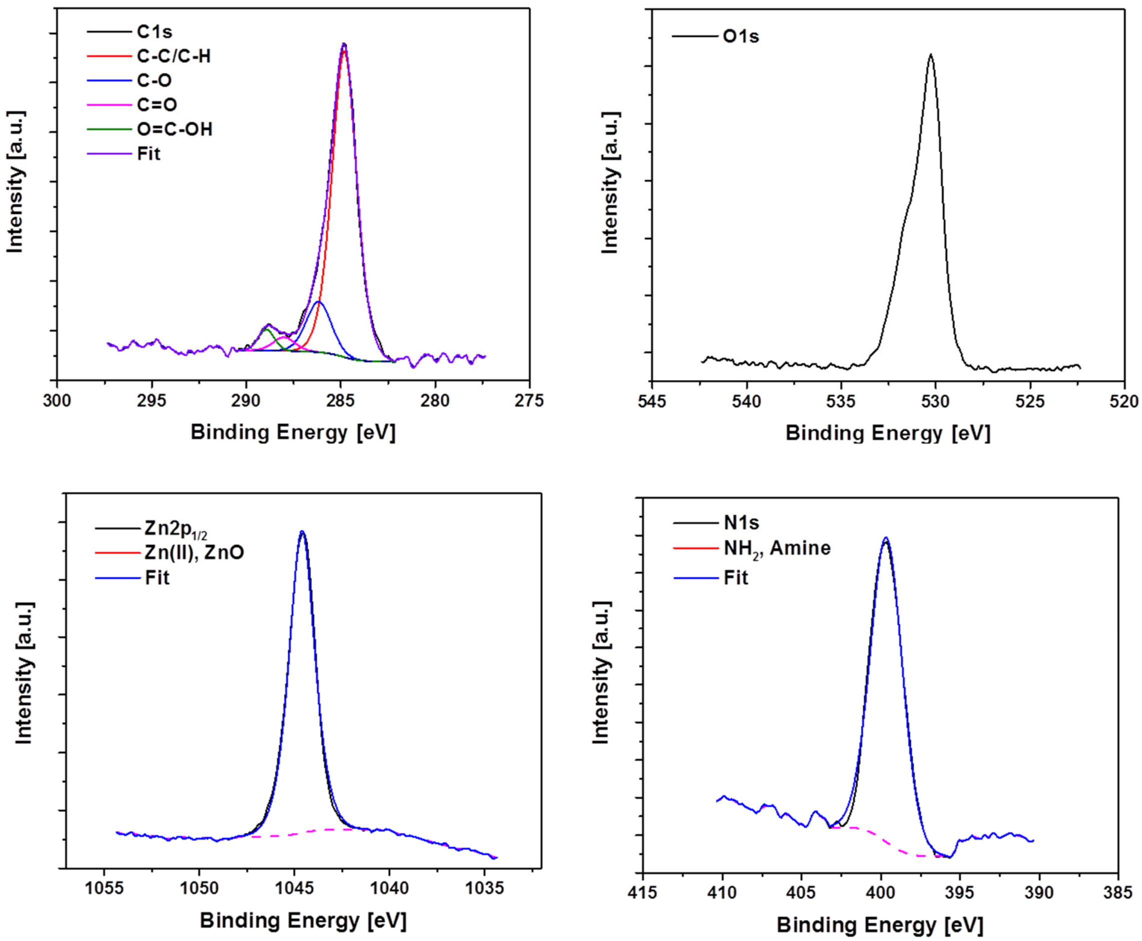

Figure S4. High-Resolution XPS spectra for Fe6:ZnO NPs.

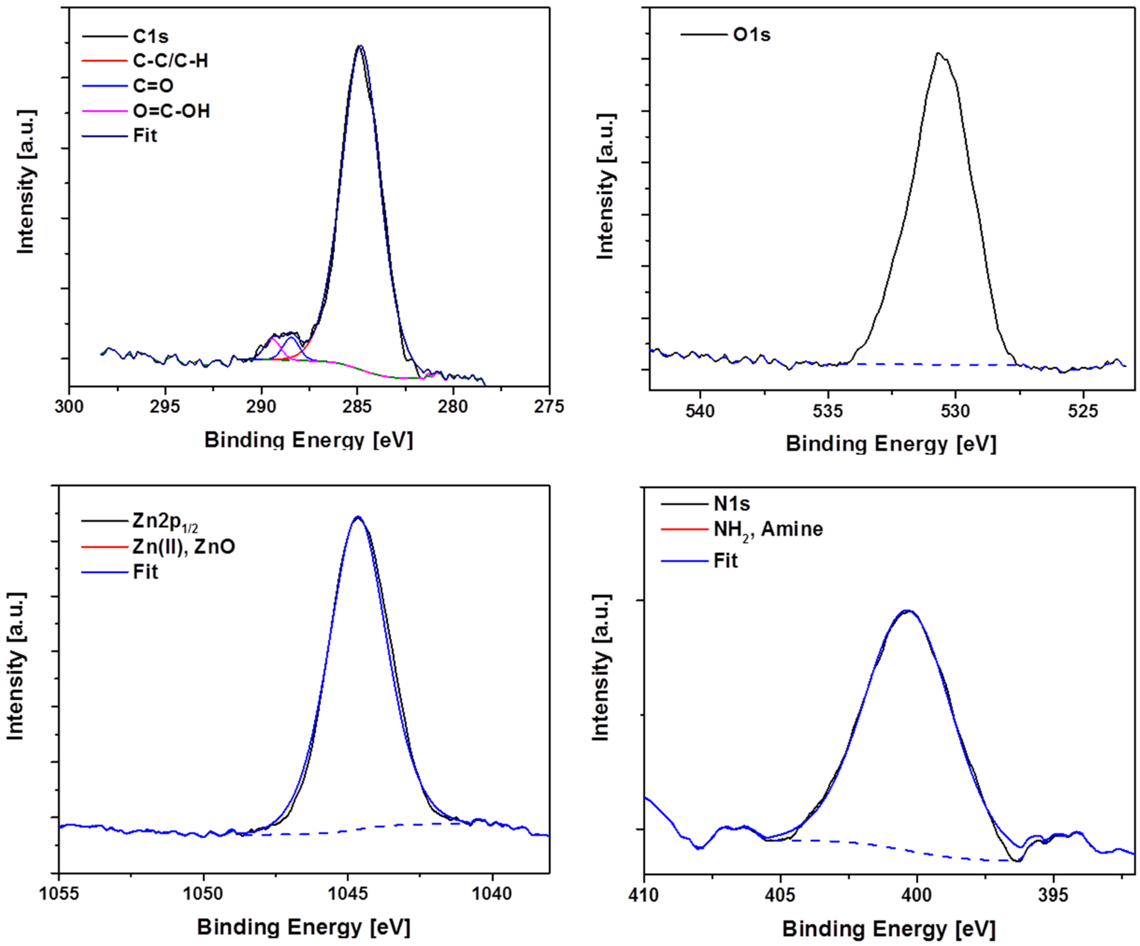

Figure S5. High-Resolution XPS spectra for Fe12:ZnO NPs.

Table S1. FeO and Fe_2O_3 peak components (binding energy and % area) obtained by deconvolution of HR $Fe2p_{3/2}$ XPS spectrum.

Sample	Fe (II), FeO		Fe (III), Fe_2O_3	
	Binding Energy [eV]	Area [%]	Binding Energy, [eV]	Area [%]
Fe6:ZnO	708.5	7.1	709.8	10.6
	709.6	37.1	710.7	27.4
			711.6	14.7
			712.6	3.1
		**44.2**		**55.8**
Fe12:ZnO	708.4	19.2	709.9	0.9
	709.6	43.3	710.7	22.9
			711.6	11.6
			712.7	2.1
		**62.5**		**37.5**

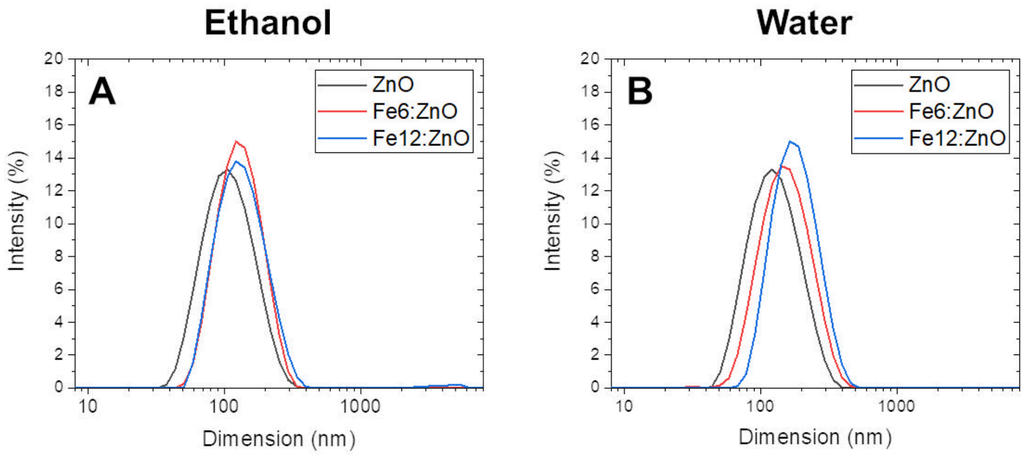

Figure S6. Dynamic light scattering results for the NPs. The graphs shows the intensity of the signal expressed in percentage of ZnO and Fe:ZnO NPs in ethanol (A) and water (B).

Statistical analysis for ZnO and Fe-ZnO cytotoxicity on B lymphocytes (IST-EBV) and Daudi cell lines. Bonferroni t-test (Three Ways ANOVA)

Comparison between factor: Nanoparticle						
		Mean Diff.	SEM	t Value	Prob	Sig
ZnO	Fe6:ZnO	-4,38333	3,82172	-1,14695	0,76529	0
ZnO	Fe12:ZnO	-7,95006	3,87444	-2,05193	0,13115	0
Fe6:ZnO	Fe12:ZnO	-3,56673	3,87444	-0,92058	1	0

Comparison between parameter: Dose (μg/mL)						
		Mean Diff.	SEM	t Value	Prob	Sig
0	10	42,77517	4,62736	9,24397	5,77E-13	1
0	20	67,13061	4,62736	14,50733	2,47E-22	1
0	30	80,69233	4,62736	17,4381	6,35E-27	1
0	40	92,11156	5,30794	17,35353	8,48E-27	1
10	20	24,35544	4,74757	5,13009	2,25E-05	1
10	30	37,91716	4,74757	7,98665	1,37E-10	1
10	40	49,33639	5,41306	9,11432	1,01E-12	1
20	30	13,56172	4,74757	2,85656	5,56E-02	0
20	40	24,98095	5,41306	4,61494	1,62E-04	1
30	40	11,41922	5,41306	2,10957	0,38278	0

Comparison between parameter: Cell line						
		Mean Diff.	SEM	t Value	Prob	Sig
IST-EBV	Daudi	33,46824	3,14918	10,6276	1,51E-16	1

All pairwise multiple comparisons										
NP	Dose (μg/mL)	Cell line	NP	Dose (μg/mL)	Cell line	Mean Diff.	SEM	t Value	Prob	Sig
ZnO	0	--	ZnO	10	--	42,47643	7,80105	5,44496	6,78E-05	1
ZnO	0	--	ZnO	20	--	73,31089	7,80105	9,39756	3,11E-12	1
ZnO	0	--	ZnO	30	--	89,09258	7,80105	11,42059	5,66E-16	1
ZnO	0	--	ZnO	40	--	98,38542	9,5543	10,2975	6,47E-14	1
ZnO	0	--	Fe6:ZnO	0	--	-2,84E-14	7,80105	-3,64E-15	1	0
ZnO	0	--	Fe6:ZnO	10	--	46,70689	7,80105	5,98725	7,44E-06	1
ZnO	0	--	Fe6:ZnO	20	--	61,18208	7,80105	7,8428	2,70E-09	1
ZnO	0	--	Fe6:ZnO	30	--	78,83601	7,80105	10,10582	1,47E-13	1
ZnO	0	--	Fe6:ZnO	40	--	94,6237	9,5543	9,90378	3,50E-13	1
ZnO	0	--	Fe12:ZnO	0	--	-1,56E-13	7,80105	-2,00E-14	1	0
ZnO	0	--	Fe12:ZnO	10	--	39,1422	8,4261	4,64535	0,00152	1
ZnO	0	--	Fe12:ZnO	20	--	66,89887	8,4261	7,93948	1,77E-09	1
ZnO	0	--	Fe12:ZnO	30	--	74,14841	8,4261	8,79985	4,17E-11	1
ZnO	0	--	Fe12:ZnO	40	--	83,32555	8,4261	9,88898	3,73E-13	1
ZnO	10	--	ZnO	20	--	30,83446	7,80105	3,9526	0,01839	1
ZnO	10	--	ZnO	30	--	46,61616	7,80105	5,97562	7,81E-06	1

ZnO	10	--	ZnO	40	--	55,909	9,5543	5,85171	1,30E-05	1
ZnO	10	--	Fe6:ZnO	0	--	-42,47643	7,80105	-5,44496	6,78E-05	1
ZnO	10	--	Fe6:ZnO	10	--	4,23046	7,80105	0,54229	1	0
ZnO	10	--	Fe6:ZnO	20	--	18,70565	7,80105	2,39784	1	0
ZnO	10	--	Fe6:ZnO	30	--	36,35959	7,80105	4,66086	0,00143	1
ZnO	10	--	Fe6:ZnO	40	--	52,14728	9,5543	5,45799	6,44E-05	1
ZnO	10	--	Fe12:ZnO	0	--	-42,47643	7,80105	-5,44496	6,78E-05	1
ZnO	10	--	Fe12:ZnO	10	--	-3,33423	8,4261	-0,3957	1	0
ZnO	10	--	Fe12:ZnO	20	--	24,42244	8,4261	2,89843	0,51786	0
ZnO	10	--	Fe12:ZnO	30	--	31,67198	8,4261	3,7588	0,03554	1
ZnO	10	--	Fe12:ZnO	40	--	40,84912	8,4261	4,84793	7,04E-04	1
ZnO	20	--	ZnO	30	--	15,78169	7,80105	2,02302	1	0
ZnO	20	--	ZnO	40	--	25,07453	9,5543	2,62442	1	0
ZnO	20	--	Fe6:ZnO	0	--	-73,31089	7,80105	-9,39756	3,11E-12	1
ZnO	20	--	Fe6:ZnO	10	--	-26,604	7,80105	-3,41031	0,11067	0
ZnO	20	--	Fe6:ZnO	20	--	-12,12881	7,80105	-1,55477	1	0
ZnO	20	--	Fe6:ZnO	30	--	5,52512	7,80105	0,70825	1	0
ZnO	20	--	Fe6:ZnO	40	--	21,31281	9,5543	2,2307	1	0
ZnO	20	--	Fe12:ZnO	0	--	-73,31089	7,80105	-9,39756	3,11E-12	1
ZnO	20	--	Fe12:ZnO	10	--	-34,16869	8,4261	-4,0551	0,01288	1
ZnO	20	--	Fe12:ZnO	20	--	-6,41202	8,4261	-0,76097	1	0
ZnO	20	--	Fe12:ZnO	30	--	0,83752	8,4261	0,0994	1	0
ZnO	20	--	Fe12:ZnO	40	--	10,01466	8,4261	1,18853	1	0
ZnO	30	--	ZnO	40	--	9,29284	9,5543	0,97263	1	0
ZnO	30	--	Fe6:ZnO	0	--	-89,09258	7,80105	-11,4206	5,66E-16	1
ZnO	30	--	Fe6:ZnO	10	--	-42,3857	7,80105	-5,43333	7,11E-05	1
ZnO	30	--	Fe6:ZnO	20	--	-27,9105	7,80105	-3,57779	0,06464	0
ZnO	30	--	Fe6:ZnO	30	--	-10,25657	7,80105	-1,31477	1	0
ZnO	30	--	Fe6:ZnO	40	--	5,53112	9,5543	0,57891	1	0
ZnO	30	--	Fe12:ZnO	0	--	-89,09258	7,80105	-11,4206	5,66E-16	1
ZnO	30	--	Fe12:ZnO	10	--	-49,95038	8,4261	-5,92806	9,50E-06	1
ZnO	30	--	Fe12:ZnO	20	--	-22,19371	8,4261	-2,63393	1	0
ZnO	30	--	Fe12:ZnO	30	--	-14,94418	8,4261	-1,77356	1	0
ZnO	30	--	Fe12:ZnO	40	--	-5,76703	8,4261	-0,68442	1	0
ZnO	40	--	Fe6:ZnO	0	--	-98,38542	9,5543	-10,2975	6,47E-14	1
ZnO	40	--	Fe6:ZnO	10	--	-51,67854	9,5543	-5,40893	7,84E-05	1
ZnO	40	--	Fe6:ZnO	20	--	-37,20334	9,5543	-3,89389	0,0225	1
ZnO	40	--	Fe6:ZnO	30	--	-19,54941	9,5543	-2,04614	1	0
ZnO	40	--	Fe6:ZnO	40	--	-3,76172	11,03235	-0,34097	1	0
ZnO	40	--	Fe12:ZnO	0	--	-98,38542	9,5543	-10,2975	6,47E-14	1
ZnO	40	--	Fe12:ZnO	10	--	-59,24322	10,07111	-5,88249	1,15E-05	1
ZnO	40	--	Fe12:ZnO	20	--	-31,48655	10,07111	-3,12642	0,26554	0
ZnO	40	--	Fe12:ZnO	30	--	-24,23701	10,07111	-2,40659	1	0
ZnO	40	--	Fe12:ZnO	40	--	-15,05987	10,07111	-1,49535	1	0
Fe6:ZnO	0	--	Fe6:ZnO	10	--	46,70689	7,80105	5,98725	7,44E-06	1
Fe6:ZnO	0	--	Fe6:ZnO	20	--	61,18208	7,80105	7,8428	2,70E-09	1

Fe6:ZnO	0	--	Fe6:ZnO	30	--	78,83601	7,80105	10,10582	1,47E-13	1
Fe6:ZnO	0	--	Fe6:ZnO	40	--	94,6237	9,5543	9,90378	3,50E-13	1
Fe6:ZnO	0	--	Fe12:ZnO	0	--	-1,28E-13	7,80105	-1,64E-14	1	0
Fe6:ZnO	0	--	Fe12:ZnO	10	--	39,1422	8,4261	4,64535	0,00152	1
Fe6:ZnO	0	--	Fe12:ZnO	20	--	66,89887	8,4261	7,93948	1,77E-09	1
Fe6:ZnO	0	--	Fe12:ZnO	30	--	74,14841	8,4261	8,79985	4,17E-11	1
Fe6:ZnO	0	--	Fe12:ZnO	40	--	83,32555	8,4261	9,88898	3,73E-13	1
Fe6:ZnO	10	--	Fe6:ZnO	20	--	14,47519	7,80105	1,85554	1	0
Fe6:ZnO	10	--	Fe6:ZnO	30	--	32,12913	7,80105	4,11856	0,01031	1
Fe6:ZnO	10	--	Fe6:ZnO	40	--	47,91682	9,5543	5,01521	3,70E-04	1
Fe6:ZnO	10	--	Fe12:ZnO	0	--	-46,70689	7,80105	-5,98725	7,44E-06	1
Fe6:ZnO	10	--	Fe12:ZnO	10	--	-7,56469	8,4261	-0,89777	1	0
Fe6:ZnO	10	--	Fe12:ZnO	20	--	20,19198	8,4261	2,39636	1	0
Fe6:ZnO	10	--	Fe12:ZnO	30	--	27,44152	8,4261	3,25673	0,17873	0
Fe6:ZnO	10	--	Fe12:ZnO	40	--	36,61867	8,4261	4,34586	0,00457	1
Fe6:ZnO	20	--	Fe6:ZnO	30	--	17,65394	7,80105	2,26302	1	0
Fe6:ZnO	20	--	Fe6:ZnO	40	--	33,44163	9,5543	3,50017	0,0831	0
Fe6:ZnO	20	--	Fe12:ZnO	0	--	-61,18208	7,80105	-7,8428	2,70E-09	1
Fe6:ZnO	20	--	Fe12:ZnO	10	--	-22,03988	8,4261	-2,61567	1	0
Fe6:ZnO	20	--	Fe12:ZnO	20	--	5,71679	8,4261	0,67846	1	0
Fe6:ZnO	20	--	Fe12:ZnO	30	--	12,96633	8,4261	1,53883	1	0
Fe6:ZnO	20	--	Fe12:ZnO	40	--	22,14347	8,4261	2,62796	1	0
Fe6:ZnO	30	--	Fe6:ZnO	40	--	15,78769	9,5543	1,65242	1	0
Fe6:ZnO	30	--	Fe12:ZnO	0	--	-78,83601	7,80105	-10,1058	1,47E-13	1
Fe6:ZnO	30	--	Fe12:ZnO	10	--	-39,69381	8,4261	-4,71082	0,00118	1
Fe6:ZnO	30	--	Fe12:ZnO	20	--	-11,93715	8,4261	-1,41669	1	0
Fe6:ZnO	30	--	Fe12:ZnO	30	--	-4,68761	8,4261	-0,55632	1	0
Fe6:ZnO	30	--	Fe12:ZnO	40	--	4,48954	8,4261	0,53281	1	0
Fe6:ZnO	40	--	Fe12:ZnO	0	--	-94,6237	9,5543	-9,90378	3,50E-13	1
Fe6:ZnO	40	--	Fe12:ZnO	10	--	-55,4815	10,07111	-5,50897	5,25E-05	1
Fe6:ZnO	40	--	Fe12:ZnO	20	--	-27,72483	10,07111	-2,75291	0,77976	0
Fe6:ZnO	40	--	Fe12:ZnO	30	--	-20,4753	10,07111	-2,03307	1	0
Fe6:ZnO	40	--	Fe12:ZnO	40	--	-11,29815	10,07111	-1,12184	1	0
Fe12:ZnO	0	--	Fe12:ZnO	10	--	39,1422	8,4261	4,64535	0,00152	1
Fe12:ZnO	0	--	Fe12:ZnO	20	--	66,89887	8,4261	7,93948	1,77E-09	1
Fe12:ZnO	0	--	Fe12:ZnO	30	--	74,14841	8,4261	8,79985	4,17E-11	1
Fe12:ZnO	0	--	Fe12:ZnO	40	--	83,32555	8,4261	9,88898	3,73E-13	1
Fe12:ZnO	10	--	Fe12:ZnO	20	--	27,75667	9,00788	3,08138	0,30377	0
Fe12:ZnO	10	--	Fe12:ZnO	30	--	35,00621	9,00788	3,88618	0,0231	1
Fe12:ZnO	10	--	Fe12:ZnO	40	--	44,18335	9,00788	4,90497	5,66E-04	1
Fe12:ZnO	20	--	Fe12:ZnO	30	--	7,24954	9,00788	0,8048	1	0
Fe12:ZnO	20	--	Fe12:ZnO	40	--	16,42668	9,00788	1,82359	1	0
Fe12:ZnO	30	--	Fe12:ZnO	40	--	9,17714	9,00788	1,01879	1	0
ZnO	--	IST-EBV	ZnO	--	Daudi	26,91992	5,40473	4,98081	6,03E-05	1
ZnO	--	IST-EBV	Fe6:ZnO	--	IST-EBV	-10,01321	5,40473	-1,85268	1	0
ZnO	--	IST-EBV	Fe6:ZnO	--	Daudi	28,16647	5,40473	5,21145	2,45E-05	1

ZnO	--	IST-EBV	Fe12:ZnO	--	IST-EBV	-12,14266	5,47928	-2,21611	0,44638	0
ZnO	--	IST-EBV	Fe12:ZnO	--	Daudi	23,16246	5,47928	4,22728	0,001	1
ZnO	--	Daudi	Fe6:ZnO	--	IST-EBV	-36,93312	5,40473	-6,83349	3,01E-08	1
ZnO	--	Daudi	Fe6:ZnO	--	Daudi	1,24655	5,40473	0,23064	1	0
ZnO	--	Daudi	Fe12:ZnO	--	IST-EBV	-39,06258	5,47928	-7,12915	8,46E-09	1
ZnO	--	Daudi	Fe12:ZnO	--	Daudi	-3,75746	5,47928	-0,68576	1	0
Fe6:ZnO	--	IST-EBV	Fe6:ZnO	--	Daudi	38,17968	5,40473	7,06413	1,12E-08	1
Fe6:ZnO	--	IST-EBV	Fe12:ZnO	--	IST-EBV	-2,12945	5,47928	-0,38864	1	0
Fe6:ZnO	--	IST-EBV	Fe12:ZnO	--	Daudi	33,17567	5,47928	6,05475	8,03E-07	1
Fe6:ZnO	--	Daudi	Fe12:ZnO	--	IST-EBV	-40,30913	5,47928	-7,35665	3,17E-09	1
Fe6:ZnO	--	Daudi	Fe12:ZnO	--	Daudi	-5,00401	5,47928	-0,91326	1	0
Fe12:ZnO	--	IST-EBV	Fe12:ZnO	--	Daudi	35,30512	5,55283	6,35804	2,26E-07	1
--	0	IST-EBV	--	0	Daudi	5,68E-14	6,36953	8,92E-15	1	0
--	0	IST-EBV	--	10	IST-EBV	18,78562	6,54407	2,87063	0,24024	0
--	0	IST-EBV	--	10	Daudi	66,76472	6,54407	10,20232	4,17E-14	1
--	0	IST-EBV	--	20	IST-EBV	34,41693	6,54407	5,25925	6,09E-05	1
--	0	IST-EBV	--	20	Daudi	99,84429	6,54407	15,25721	6,70E-23	1
--	0	IST-EBV	--	30	IST-EBV	61,57196	6,54407	9,40882	1,27E-12	1
--	0	IST-EBV	--	30	Daudi	99,8127	6,54407	15,25238	6,82E-23	1
--	0	IST-EBV	--	40	IST-EBV	84,26456	7,50657	11,22545	5,48E-16	1
--	0	IST-EBV	--	40	Daudi	99,95856	7,50657	13,31615	1,11E-19	1
--	0	Daudi	--	10	IST-EBV	18,78562	6,54407	2,87063	0,24024	0
--	0	Daudi	--	10	Daudi	66,76472	6,54407	10,20232	4,17E-14	1
--	0	Daudi	--	20	IST-EBV	34,41693	6,54407	5,25925	6,09E-05	1
--	0	Daudi	--	20	Daudi	99,84429	6,54407	15,25721	6,70E-23	1
--	0	Daudi	--	30	IST-EBV	61,57196	6,54407	9,40882	1,27E-12	1
--	0	Daudi	--	30	Daudi	99,8127	6,54407	15,25238	6,82E-23	1
--	0	Daudi	--	40	IST-EBV	84,26456	7,50657	11,22545	5,48E-16	1
--	0	Daudi	--	40	Daudi	99,95856	7,50657	13,31615	1,11E-19	1
--	10	IST-EBV	--	10	Daudi	47,97909	6,71408	7,14605	2,36E-08	1
--	10	IST-EBV	--	20	IST-EBV	15,63131	6,71408	2,32814	1	0
--	10	IST-EBV	--	20	Daudi	81,05867	6,71408	12,07294	1,64E-17	1
--	10	IST-EBV	--	30	IST-EBV	42,78634	6,71408	6,37263	6,38E-07	1
--	10	IST-EBV	--	30	Daudi	81,02708	6,71408	12,06824	1,67E-17	1
--	10	IST-EBV	--	40	IST-EBV	65,47894	7,65522	8,5535	5,23E-11	1
--	10	IST-EBV	--	40	Daudi	81,17293	7,65522	10,6036	7,54E-15	1
--	10	Daudi	--	20	IST-EBV	-32,34779	6,71408	-4,81791	3,38E-04	1
--	10	Daudi	--	20	Daudi	33,07958	6,71408	4,9269	2,23E-04	1
--	10	Daudi	--	30	IST-EBV	-5,19275	6,71408	-0,77341	1	0
--	10	Daudi	--	30	Daudi	33,04799	6,71408	4,92219	2,27E-04	1
--	10	Daudi	--	40	IST-EBV	17,49984	7,65522	2,286	1	0
--	10	Daudi	--	40	Daudi	33,19384	7,65522	4,3361	0,00203	1
--	20	IST-EBV	--	20	Daudi	65,42736	6,71408	9,74481	2,97E-13	1
--	20	IST-EBV	--	30	IST-EBV	27,15504	6,71408	4,04449	0,00573	1
--	20	IST-EBV	--	30	Daudi	65,39577	6,71408	9,7401	3,03E-13	1
--	20	IST-EBV	--	40	IST-EBV	49,84763	7,65522	6,51158	3,55E-07	1

--	20	IST-EBV	--	40	Daudi	65,54163	7,65522	8,56169	5,04E-11	1
--	20	Daudi	--	30	IST-EBV	-38,27233	6,71408	-5,70031	1,04E-05	1
--	20	Daudi	--	30	Daudi	-0,03159	6,71408	-0,00471	1	0
--	20	Daudi	--	40	IST-EBV	-15,57973	7,65522	-2,03518	1	0
--	20	Daudi	--	40	Daudi	0,11426	7,65522	0,01493	1	0
--	30	IST-EBV	--	30	Daudi	38,24074	6,71408	5,69561	1,06E-05	1
--	30	IST-EBV	--	40	IST-EBV	22,6926	7,65522	2,96433	0,18358	0
--	30	IST-EBV	--	40	Daudi	38,38659	7,65522	5,01443	1,59E-04	1
--	30	Daudi	--	40	IST-EBV	-15,54814	7,65522	-2,03105	1	0
--	30	Daudi	--	40	Daudi	0,14585	7,65522	0,01905	1	0
--	40	IST-EBV	--	40	Daudi	15,694	8,49271	1,84794	1	0
ZnO	0	IST-EBV	ZnO	0	Daudi	8,53E-14	11,03235	7,73E-15	1	0
ZnO	0	IST-EBV	ZnO	10	IST-EBV	13,92063	11,03235	1,2618	1	0
ZnO	0	IST-EBV	ZnO	10	Daudi	71,03223	11,03235	6,43854	4,67E-06	1
ZnO	0	IST-EBV	ZnO	20	IST-EBV	47,0889	11,03235	4,26826	0,02507	1
ZnO	0	IST-EBV	ZnO	20	Daudi	99,53288	11,03235	9,02191	6,58E-11	1
ZnO	0	IST-EBV	ZnO	30	IST-EBV	78,18516	11,03235	7,0869	2,94E-07	1
ZnO	0	IST-EBV	ZnO	30	Daudi	100	11,03235	9,06425	5,47E-11	1
ZnO	0	IST-EBV	ZnO	40	IST-EBV	96,77084	13,51182	7,16194	2,13E-07	1
ZnO	0	IST-EBV	ZnO	40	Daudi	100	13,51182	7,40093	7,59E-08	1
ZnO	0	IST-EBV	Fe6:ZnO	0	IST-EBV	-4,26E-14	11,03235	-3,86E-15	1	0
ZnO	0	IST-EBV	Fe6:ZnO	0	Daudi	2,84E-14	11,03235	2,58E-15	1	0
ZnO	0	IST-EBV	Fe6:ZnO	10	IST-EBV	16,6159	11,03235	1,50611	1	0
ZnO	0	IST-EBV	Fe6:ZnO	10	Daudi	76,79787	11,03235	6,96115	5,05E-07	1
ZnO	0	IST-EBV	Fe6:ZnO	20	IST-EBV	22,36416	11,03235	2,02714	1	0
ZnO	0	IST-EBV	Fe6:ZnO	20	Daudi	100	11,03235	9,06425	5,47E-11	1
ZnO	0	IST-EBV	Fe6:ZnO	30	IST-EBV	57,67203	11,03235	5,22754	6,67E-04	1
ZnO	0	IST-EBV	Fe6:ZnO	30	Daudi	100	11,03235	9,06425	5,47E-11	1
ZnO	0	IST-EBV	Fe6:ZnO	40	IST-EBV	89,24741	13,51182	6,60514	2,31E-06	1
ZnO	0	IST-EBV	Fe6:ZnO	40	Daudi	100	13,51182	7,40093	7,59E-08	1
ZnO	0	IST-EBV	Fe12:ZnO	0	IST-EBV	-9,95E-14	11,03235	-9,02E-15	1	0
ZnO	0	IST-EBV	Fe12:ZnO	0	Daudi	-1,28E-13	11,03235	-1,16E-14	1	0
ZnO	0	IST-EBV	Fe12:ZnO	10	IST-EBV	25,82035	11,9163	2,16681	1	0
ZnO	0	IST-EBV	Fe12:ZnO	10	Daudi	52,46405	11,9163	4,40271	0,0154	1
ZnO	0	IST-EBV	Fe12:ZnO	20	IST-EBV	33,79774	11,9163	2,83626	1	0
ZnO	0	IST-EBV	Fe12:ZnO	20	Daudi	100	11,9163	8,39186	1,02E-09	1
ZnO	0	IST-EBV	Fe12:ZnO	30	IST-EBV	48,8587	11,9163	4,10016	0,04557	1
ZnO	0	IST-EBV	Fe12:ZnO	30	Daudi	99,43811	11,9163	8,34471	1,26E-09	1
ZnO	0	IST-EBV	Fe12:ZnO	40	IST-EBV	66,77543	11,9163	5,6037	1,48E-04	1
ZnO	0	IST-EBV	Fe12:ZnO	40	Daudi	99,87567	11,9163	8,38143	1,07E-09	1
ZnO	0	Daudi	ZnO	10	IST-EBV	13,92063	11,03235	1,2618	1	0
ZnO	0	Daudi	ZnO	10	Daudi	71,03223	11,03235	6,43854	4,67E-06	1
ZnO	0	Daudi	ZnO	20	IST-EBV	47,0889	11,03235	4,26826	0,02507	1
ZnO	0	Daudi	ZnO	20	Daudi	99,53288	11,03235	9,02191	6,58E-11	1
ZnO	0	Daudi	ZnO	30	IST-EBV	78,18516	11,03235	7,0869	2,94E-07	1
ZnO	0	Daudi	ZnO	30	Daudi	100	11,03235	9,06425	5,47E-11	1

ZnO	0	Daudi	ZnO	40	IST-EBV	96,77084	13,51182	7,16194	2,13E-07	1
ZnO	0	Daudi	ZnO	40	Daudi	100	13,51182	7,40093	7,59E-08	1
ZnO	0	Daudi	Fe6:ZnO	0	IST-EBV	-1,28E-13	11,03235	-1,16E-14	1	0
ZnO	0	Daudi	Fe6:ZnO	0	Daudi	-5,68E-14	11,03235	-5,15E-15	1	0
ZnO	0	Daudi	Fe6:ZnO	10	IST-EBV	16,6159	11,03235	1,50611	1	0
ZnO	0	Daudi	Fe6:ZnO	10	Daudi	76,79787	11,03235	6,96115	5,05E-07	1
ZnO	0	Daudi	Fe6:ZnO	20	IST-EBV	22,36416	11,03235	2,02714	1	0
ZnO	0	Daudi	Fe6:ZnO	20	Daudi	100	11,03235	9,06425	5,47E-11	1
ZnO	0	Daudi	Fe6:ZnO	30	IST-EBV	57,67203	11,03235	5,22754	6,67E-04	1
ZnO	0	Daudi	Fe6:ZnO	30	Daudi	100	11,03235	9,06425	5,47E-11	1
ZnO	0	Daudi	Fe6:ZnO	40	IST-EBV	89,24741	13,51182	6,60514	2,31E-06	1
ZnO	0	Daudi	Fe6:ZnO	40	Daudi	100	13,51182	7,40093	7,59E-08	1
ZnO	0	Daudi	Fe12:ZnO	0	IST-EBV	-1,85E-13	11,03235	-1,67E-14	1	0
ZnO	0	Daudi	Fe12:ZnO	0	Daudi	-2,13E-13	11,03235	-1,93E-14	1	0
ZnO	0	Daudi	Fe12:ZnO	10	IST-EBV	25,82035	11,9163	2,16681	1	0
ZnO	0	Daudi	Fe12:ZnO	10	Daudi	52,46405	11,9163	4,40271	0,0154	1
ZnO	0	Daudi	Fe12:ZnO	20	IST-EBV	33,79774	11,9163	2,83626	1	0
ZnO	0	Daudi	Fe12:ZnO	20	Daudi	100	11,9163	8,39186	1,02E-09	1
ZnO	0	Daudi	Fe12:ZnO	30	IST-EBV	48,8587	11,9163	4,10016	0,04557	1
ZnO	0	Daudi	Fe12:ZnO	30	Daudi	99,43811	11,9163	8,34471	1,26E-09	1
ZnO	0	Daudi	Fe12:ZnO	40	IST-EBV	66,77543	11,9163	5,6037	1,48E-04	1
ZnO	0	Daudi	Fe12:ZnO	40	Daudi	99,87567	11,9163	8,38143	1,07E-09	1
ZnO	10	IST-EBV	ZnO	10	Daudi	57,1116	11,03235	5,17674	8,15E-04	1
ZnO	10	IST-EBV	ZnO	20	IST-EBV	33,16827	11,03235	3,00645	1	0
ZnO	10	IST-EBV	ZnO	20	Daudi	85,61226	11,03235	7,76011	1,60E-08	1
ZnO	10	IST-EBV	ZnO	30	IST-EBV	64,26454	11,03235	5,8251	6,01E-05	1
ZnO	10	IST-EBV	ZnO	30	Daudi	86,07937	11,03235	7,80245	1,33E-08	1
ZnO	10	IST-EBV	ZnO	40	IST-EBV	82,85022	13,51182	6,13169	1,69E-05	1
ZnO	10	IST-EBV	ZnO	40	Daudi	86,07937	13,51182	6,37067	6,22E-06	1
ZnO	10	IST-EBV	Fe6:ZnO	0	IST-EBV	-13,92063	11,03235	-1,2618	1	0
ZnO	10	IST-EBV	Fe6:ZnO	0	Daudi	-13,92063	11,03235	-1,2618	1	0
ZnO	10	IST-EBV	Fe6:ZnO	10	IST-EBV	2,69528	11,03235	0,24431	1	0
ZnO	10	IST-EBV	Fe6:ZnO	10	Daudi	62,87724	11,03235	5,69935	1,01E-04	1
ZnO	10	IST-EBV	Fe6:ZnO	20	IST-EBV	8,44353	11,03235	0,76534	1	0
ZnO	10	IST-EBV	Fe6:ZnO	20	Daudi	86,07937	11,03235	7,80245	1,33E-08	1
ZnO	10	IST-EBV	Fe6:ZnO	30	IST-EBV	43,7514	11,03235	3,96574	0,07281	0
ZnO	10	IST-EBV	Fe6:ZnO	30	Daudi	86,07937	11,03235	7,80245	1,33E-08	1
ZnO	10	IST-EBV	Fe6:ZnO	40	IST-EBV	75,32678	13,51182	5,57488	1,67E-04	1
ZnO	10	IST-EBV	Fe6:ZnO	40	Daudi	86,07937	13,51182	6,37067	6,22E-06	1
ZnO	10	IST-EBV	Fe12:ZnO	0	IST-EBV	-13,92063	11,03235	-1,2618	1	0
ZnO	10	IST-EBV	Fe12:ZnO	0	Daudi	-13,92063	11,03235	-1,2618	1	0
ZnO	10	IST-EBV	Fe12:ZnO	10	IST-EBV	11,89972	11,9163	0,99861	1	0
ZnO	10	IST-EBV	Fe12:ZnO	10	Daudi	38,54343	11,9163	3,23451	0,79271	0
ZnO	10	IST-EBV	Fe12:ZnO	20	IST-EBV	19,87711	11,9163	1,66806	1	0
ZnO	10	IST-EBV	Fe12:ZnO	20	Daudi	86,07937	11,9163	7,22366	1,63E-07	1
ZnO	10	IST-EBV	Fe12:ZnO	30	IST-EBV	34,93808	11,9163	2,93196	1	0

ZnO	10	IST-EBV	Fe12:ZnO	30	Daudi	85,51748	11,9163	7,17651	2,00E-07	1
ZnO	10	IST-EBV	Fe12:ZnO	40	IST-EBV	52,85481	11,9163	4,4355	0,01366	1
ZnO	10	IST-EBV	Fe12:ZnO	40	Daudi	85,95504	11,9163	7,21323	1,71E-07	1
ZnO	10	Daudi	ZnO	20	IST-EBV	-23,94333	11,03235	-2,17028	1	0
ZnO	10	Daudi	ZnO	20	Daudi	28,50066	11,03235	2,58337	1	0
ZnO	10	Daudi	ZnO	30	IST-EBV	7,15294	11,03235	0,64836	1	0
ZnO	10	Daudi	ZnO	30	Daudi	28,96777	11,03235	2,62571	1	0
ZnO	10	Daudi	ZnO	40	IST-EBV	25,73862	13,51182	1,9049	1	0
ZnO	10	Daudi	ZnO	40	Daudi	28,96777	13,51182	2,14388	1	0
ZnO	10	Daudi	Fe6:ZnO	0	IST-EBV	-71,03223	11,03235	-6,43854	4,67E-06	1
ZnO	10	Daudi	Fe6:ZnO	0	Daudi	-71,03223	11,03235	-6,43854	4,67E-06	1
ZnO	10	Daudi	Fe6:ZnO	10	IST-EBV	-54,41632	11,03235	-4,93243	0,00211	1
ZnO	10	Daudi	Fe6:ZnO	10	Daudi	5,76564	11,03235	0,52261	1	0
ZnO	10	Daudi	Fe6:ZnO	20	IST-EBV	-48,66807	11,03235	-4,4114	0,01492	1
ZnO	10	Daudi	Fe6:ZnO	20	Daudi	28,96777	11,03235	2,62571	1	0
ZnO	10	Daudi	Fe6:ZnO	30	IST-EBV	-13,3602	11,03235	-1,211	1	0
ZnO	10	Daudi	Fe6:ZnO	30	Daudi	28,96777	11,03235	2,62571	1	0
ZnO	10	Daudi	Fe6:ZnO	40	IST-EBV	18,21518	13,51182	1,34809	1	0
ZnO	10	Daudi	Fe6:ZnO	40	Daudi	28,96777	13,51182	2,14388	1	0
ZnO	10	Daudi	Fe12:ZnO	0	IST-EBV	-71,03223	11,03235	-6,43854	4,67E-06	1
ZnO	10	Daudi	Fe12:ZnO	0	Daudi	-71,03223	11,03235	-6,43854	4,67E-06	1
ZnO	10	Daudi	Fe12:ZnO	10	IST-EBV	-45,21188	11,9163	-3,79412	0,13077	0
ZnO	10	Daudi	Fe12:ZnO	10	Daudi	-18,56817	11,9163	-1,55822	1	0
ZnO	10	Daudi	Fe12:ZnO	20	IST-EBV	-37,23449	11,9163	-3,12467	1	0
ZnO	10	Daudi	Fe12:ZnO	20	Daudi	28,96777	11,9163	2,43094	1	0
ZnO	10	Daudi	Fe12:ZnO	30	IST-EBV	-22,17352	11,9163	-1,86077	1	0
ZnO	10	Daudi	Fe12:ZnO	30	Daudi	28,40588	11,9163	2,38378	1	0
ZnO	10	Daudi	Fe12:ZnO	40	IST-EBV	-4,25679	11,9163	-0,35722	1	0
ZnO	10	Daudi	Fe12:ZnO	40	Daudi	28,84344	11,9163	2,4205	1	0
ZnO	20	IST-EBV	ZnO	20	Daudi	52,44399	11,03235	4,75365	0,00417	1
ZnO	20	IST-EBV	ZnO	30	IST-EBV	31,09627	11,03235	2,81864	1	0
ZnO	20	IST-EBV	ZnO	30	Daudi	52,9111	11,03235	4,79599	0,00355	1
ZnO	20	IST-EBV	ZnO	40	IST-EBV	49,68194	13,51182	3,67693	0,1934	0
ZnO	20	IST-EBV	ZnO	40	Daudi	52,9111	13,51182	3,91591	0,08643	0
ZnO	20	IST-EBV	Fe6:ZnO	0	IST-EBV	-47,0889	11,03235	-4,26826	0,02507	1
ZnO	20	IST-EBV	Fe6:ZnO	0	Daudi	-47,0889	11,03235	-4,26826	0,02507	1
ZnO	20	IST-EBV	Fe6:ZnO	10	IST-EBV	-30,47299	11,03235	-2,76215	1	0
ZnO	20	IST-EBV	Fe6:ZnO	10	Daudi	29,70897	11,03235	2,6929	1	0
ZnO	20	IST-EBV	Fe6:ZnO	20	IST-EBV	-24,72474	11,03235	-2,24111	1	0
ZnO	20	IST-EBV	Fe6:ZnO	20	Daudi	52,9111	11,03235	4,79599	0,00355	1
ZnO	20	IST-EBV	Fe6:ZnO	30	IST-EBV	10,58313	11,03235	0,95928	1	0
ZnO	20	IST-EBV	Fe6:ZnO	30	Daudi	52,9111	11,03235	4,79599	0,00355	1
ZnO	20	IST-EBV	Fe6:ZnO	40	IST-EBV	42,15851	13,51182	3,12012	1	0
ZnO	20	IST-EBV	Fe6:ZnO	40	Daudi	52,9111	13,51182	3,91591	0,08643	0
ZnO	20	IST-EBV	Fe12:ZnO	0	IST-EBV	-47,0889	11,03235	-4,26826	0,02507	1
ZnO	20	IST-EBV	Fe12:ZnO	0	Daudi	-47,0889	11,03235	-4,26826	0,02507	1

ZnO	20	IST-EBV	Fe12:ZnO	10	IST-EBV	-21,26855	11,9163	-1,78483	1	0
ZnO	20	IST-EBV	Fe12:ZnO	10	Daudi	5,37516	11,9163	0,45108	1	0
ZnO	20	IST-EBV	Fe12:ZnO	20	IST-EBV	-13,29116	11,9163	-1,11538	1	0
ZnO	20	IST-EBV	Fe12:ZnO	20	Daudi	52,9111	11,9163	4,44023	0,01343	1
ZnO	20	IST-EBV	Fe12:ZnO	30	IST-EBV	1,76981	11,9163	0,14852	1	0
ZnO	20	IST-EBV	Fe12:ZnO	30	Daudi	52,34921	11,9163	4,39307	0,01596	1
ZnO	20	IST-EBV	Fe12:ZnO	40	IST-EBV	19,68654	11,9163	1,65207	1	0
ZnO	20	IST-EBV	Fe12:ZnO	40	Daudi	52,78677	11,9163	4,42979	0,01395	1
ZnO	20	Daudi	ZnO	30	IST-EBV	-21,34772	11,03235	-1,93501	1	0
ZnO	20	Daudi	ZnO	30	Daudi	0,46712	11,03235	0,04234	1	0
ZnO	20	Daudi	ZnO	40	IST-EBV	-2,76204	13,51182	-0,20442	1	0
ZnO	20	Daudi	ZnO	40	Daudi	0,46712	13,51182	0,03457	1	0
ZnO	20	Daudi	Fe6:ZnO	0	IST-EBV	-99,53288	11,03235	-9,02191	6,58E-11	1
ZnO	20	Daudi	Fe6:ZnO	0	Daudi	-99,53288	11,03235	-9,02191	6,58E-11	1
ZnO	20	Daudi	Fe6:ZnO	10	IST-EBV	-82,91698	11,03235	-7,5158	4,62E-08	1
ZnO	20	Daudi	Fe6:ZnO	10	Daudi	-22,73501	11,03235	-2,06076	1	0
ZnO	20	Daudi	Fe6:ZnO	20	IST-EBV	-77,16873	11,03235	-6,99477	4,37E-07	1
ZnO	20	Daudi	Fe6:ZnO	20	Daudi	0,46712	11,03235	0,04234	1	0
ZnO	20	Daudi	Fe6:ZnO	30	IST-EBV	-41,86085	11,03235	-3,79437	0,13066	0
ZnO	20	Daudi	Fe6:ZnO	30	Daudi	0,46712	11,03235	0,04234	1	0
ZnO	20	Daudi	Fe6:ZnO	40	IST-EBV	-10,28548	13,51182	-0,76122	1	0
ZnO	20	Daudi	Fe6:ZnO	40	Daudi	0,46712	13,51182	0,03457	1	0
ZnO	20	Daudi	Fe12:ZnO	0	IST-EBV	-99,53288	11,03235	-9,02191	6,58E-11	1
ZnO	20	Daudi	Fe12:ZnO	0	Daudi	-99,53288	11,03235	-9,02191	6,58E-11	1
ZnO	20	Daudi	Fe12:ZnO	10	IST-EBV	-73,71254	11,9163	-6,18586	1,35E-05	1
ZnO	20	Daudi	Fe12:ZnO	10	Daudi	-47,06883	11,9163	-3,94995	0,07689	0
ZnO	20	Daudi	Fe12:ZnO	20	IST-EBV	-65,73515	11,9163	-5,5164	2,11E-04	1
ZnO	20	Daudi	Fe12:ZnO	20	Daudi	0,46712	11,9163	0,0392	1	0
ZnO	20	Daudi	Fe12:ZnO	30	IST-EBV	-50,67418	11,9163	-4,25251	0,02653	1
ZnO	20	Daudi	Fe12:ZnO	30	Daudi	-0,09477	11,9163	-0,00795	1	0
ZnO	20	Daudi	Fe12:ZnO	40	IST-EBV	-32,75745	11,9163	-2,74896	1	0
ZnO	20	Daudi	Fe12:ZnO	40	Daudi	0,34279	11,9163	0,02877	1	0
ZnO	30	IST-EBV	ZnO	30	Daudi	21,81484	11,03235	1,97735	1	0
ZnO	30	IST-EBV	ZnO	40	IST-EBV	18,58568	13,51182	1,37551	1	0
ZnO	30	IST-EBV	ZnO	40	Daudi	21,81484	13,51182	1,6145	1	0
ZnO	30	IST-EBV	Fe6:ZnO	0	IST-EBV	-78,18516	11,03235	-7,0869	2,94E-07	1
ZnO	30	IST-EBV	Fe6:ZnO	0	Daudi	-78,18516	11,03235	-7,0869	2,94E-07	1
ZnO	30	IST-EBV	Fe6:ZnO	10	IST-EBV	-61,56926	11,03235	-5,58079	1,63E-04	1
ZnO	30	IST-EBV	Fe6:ZnO	10	Daudi	-1,3873	11,03235	-0,12575	1	0
ZnO	30	IST-EBV	Fe6:ZnO	20	IST-EBV	-55,82101	11,03235	-5,05976	0,00129	1
ZnO	30	IST-EBV	Fe6:ZnO	20	Daudi	21,81484	11,03235	1,97735	1	0
ZnO	30	IST-EBV	Fe6:ZnO	30	IST-EBV	-20,51314	11,03235	-1,85936	1	0
ZnO	30	IST-EBV	Fe6:ZnO	30	Daudi	21,81484	11,03235	1,97735	1	0
ZnO	30	IST-EBV	Fe6:ZnO	40	IST-EBV	11,06224	13,51182	0,81871	1	0
ZnO	30	IST-EBV	Fe6:ZnO	40	Daudi	21,81484	13,51182	1,6145	1	0
ZnO	30	IST-EBV	Fe12:ZnO	0	IST-EBV	-78,18516	11,03235	-7,0869	2,94E-07	1

ZnO	30	IST-EBV	Fe12:ZnO	0	Daudi	-78,18516	11,03235	-7,0869	2,94E-07	1
ZnO	30	IST-EBV	Fe12:ZnO	10	IST-EBV	-52,36482	11,9163	-4,39438	0,01588	1
ZnO	30	IST-EBV	Fe12:ZnO	10	Daudi	-25,72111	11,9163	-2,15848	1	0
ZnO	30	IST-EBV	Fe12:ZnO	20	IST-EBV	-44,38743	11,9163	-3,72493	0,1649	0
ZnO	30	IST-EBV	Fe12:ZnO	20	Daudi	21,81484	11,9163	1,83067	1	0
ZnO	30	IST-EBV	Fe12:ZnO	30	IST-EBV	-29,32646	11,9163	-2,46104	1	0
ZnO	30	IST-EBV	Fe12:ZnO	30	Daudi	21,25295	11,9163	1,78352	1	0
ZnO	30	IST-EBV	Fe12:ZnO	40	IST-EBV	-11,40973	11,9163	-0,95749	1	0
ZnO	30	IST-EBV	Fe12:ZnO	40	Daudi	21,6905	11,9163	1,82024	1	0
ZnO	30	Daudi	ZnO	40	IST-EBV	-3,22916	13,51182	-0,23899	1	0
ZnO	30	Daudi	ZnO	40	Daudi	-3,84E-13	13,51182	-2,84E-14	1	0
ZnO	30	Daudi	Fe6:ZnO	0	IST-EBV	-100	11,03235	-9,06425	5,47E-11	1
ZnO	30	Daudi	Fe6:ZnO	0	Daudi	-100	11,03235	-9,06425	5,47E-11	1
ZnO	30	Daudi	Fe6:ZnO	10	IST-EBV	-83,3841	11,03235	-7,55814	3,84E-08	1
ZnO	30	Daudi	Fe6:ZnO	10	Daudi	-23,20213	11,03235	-2,1031	1	0
ZnO	30	Daudi	Fe6:ZnO	20	IST-EBV	-77,63584	11,03235	-7,03711	3,65E-07	1
ZnO	30	Daudi	Fe6:ZnO	20	Daudi	-1,33E-13	11,03235	-1,20E-14	1	0
ZnO	30	Daudi	Fe6:ZnO	30	IST-EBV	-42,32797	11,03235	-3,83671	0,11323	0
ZnO	30	Daudi	Fe6:ZnO	30	Daudi	-5,37E-14	11,03235	-4,87E-15	1	0
ZnO	30	Daudi	Fe6:ZnO	40	IST-EBV	-10,75259	13,51182	-0,79579	1	0
ZnO	30	Daudi	Fe6:ZnO	40	Daudi	-2,75E-14	13,51182	-2,04E-15	1	0
ZnO	30	Daudi	Fe12:ZnO	0	IST-EBV	-100	11,03235	-9,06425	5,47E-11	1
ZnO	30	Daudi	Fe12:ZnO	0	Daudi	-100	11,03235	-9,06425	5,47E-11	1
ZnO	30	Daudi	Fe12:ZnO	10	IST-EBV	-74,17965	11,9163	-6,22506	1,15E-05	1
ZnO	30	Daudi	Fe12:ZnO	10	Daudi	-47,53595	11,9163	-3,98915	0,06714	0
ZnO	30	Daudi	Fe12:ZnO	20	IST-EBV	-66,20226	11,9163	-5,5556	1,80E-04	1
ZnO	30	Daudi	Fe12:ZnO	20	Daudi	-5,82E-14	11,9163	-4,88E-15	1	0
ZnO	30	Daudi	Fe12:ZnO	30	IST-EBV	-51,1413	11,9163	-4,29171	0,02304	1
ZnO	30	Daudi	Fe12:ZnO	30	Daudi	-0,56189	11,9163	-0,04715	1	0
ZnO	30	Daudi	Fe12:ZnO	40	IST-EBV	-33,22457	11,9163	-2,78816	1	0
ZnO	30	Daudi	Fe12:ZnO	40	Daudi	-0,12433	11,9163	-0,01043	1	0
ZnO	40	IST-EBV	ZnO	40	Daudi	3,22916	15,6021	0,20697	1	0
ZnO	40	IST-EBV	Fe6:ZnO	0	IST-EBV	-96,77084	13,51182	-7,16194	2,13E-07	1
ZnO	40	IST-EBV	Fe6:ZnO	0	Daudi	-96,77084	13,51182	-7,16194	2,13E-07	1
ZnO	40	IST-EBV	Fe6:ZnO	10	IST-EBV	-80,15494	13,51182	-5,93221	3,87E-05	1
ZnO	40	IST-EBV	Fe6:ZnO	10	Daudi	-19,97297	13,51182	-1,47819	1	0
ZnO	40	IST-EBV	Fe6:ZnO	20	IST-EBV	-74,40669	13,51182	-5,50679	2,19E-04	1
ZnO	40	IST-EBV	Fe6:ZnO	20	Daudi	3,22916	13,51182	0,23899	1	0
ZnO	40	IST-EBV	Fe6:ZnO	30	IST-EBV	-39,09881	13,51182	-2,89368	1	0
ZnO	40	IST-EBV	Fe6:ZnO	30	Daudi	3,22916	13,51182	0,23899	1	0
ZnO	40	IST-EBV	Fe6:ZnO	40	IST-EBV	-7,52344	15,6021	-0,48221	1	0
ZnO	40	IST-EBV	Fe6:ZnO	40	Daudi	3,22916	15,6021	0,20697	1	0
ZnO	40	IST-EBV	Fe12:ZnO	0	IST-EBV	-96,77084	13,51182	-7,16194	2,13E-07	1
ZnO	40	IST-EBV	Fe12:ZnO	0	Daudi	-96,77084	13,51182	-7,16194	2,13E-07	1
ZnO	40	IST-EBV	Fe12:ZnO	10	IST-EBV	-70,9505	14,24271	-4,98153	0,00174	1
ZnO	40	IST-EBV	Fe12:ZnO	10	Daudi	-44,30679	14,24271	-3,11084	1	0

ZnO	40	IST-EBV	Fe12:ZnO	20	IST-EBV	-62,9731	14,24271	-4,42143	0,01439	1
ZnO	40	IST-EBV	Fe12:ZnO	20	Daudi	3,22916	14,24271	0,22672	1	0
ZnO	40	IST-EBV	Fe12:ZnO	30	IST-EBV	-47,91214	14,24271	-3,36398	0,53059	0
ZnO	40	IST-EBV	Fe12:ZnO	30	Daudi	2,66727	14,24271	0,18727	1	0
ZnO	40	IST-EBV	Fe12:ZnO	40	IST-EBV	-29,99541	14,24271	-2,10602	1	0
ZnO	40	IST-EBV	Fe12:ZnO	40	Daudi	3,10483	14,24271	0,21799	1	0
ZnO	40	Daudi	Fe6:ZnO	0	IST-EBV	-100	13,51182	-7,40093	7,59E-08	1
ZnO	40	Daudi	Fe6:ZnO	0	Daudi	-100	13,51182	-7,40093	7,59E-08	1
ZnO	40	Daudi	Fe6:ZnO	10	IST-EBV	-83,3841	13,51182	-6,1712	1,43E-05	1
ZnO	40	Daudi	Fe6:ZnO	10	Daudi	-23,20213	13,51182	-1,71717	1	0
ZnO	40	Daudi	Fe6:ZnO	20	IST-EBV	-77,63584	13,51182	-5,74577	8,32E-05	1
ZnO	40	Daudi	Fe6:ZnO	20	Daudi	2,51E-13	13,51182	1,86E-14	1	0
ZnO	40	Daudi	Fe6:ZnO	30	IST-EBV	-42,32797	13,51182	-3,13266	1	0
ZnO	40	Daudi	Fe6:ZnO	30	Daudi	3,30E-13	13,51182	2,44E-14	1	0
ZnO	40	Daudi	Fe6:ZnO	40	IST-EBV	-10,75259	15,6021	-0,68918	1	0
ZnO	40	Daudi	Fe6:ZnO	40	Daudi	3,56E-13	15,6021	2,28E-14	1	0
ZnO	40	Daudi	Fe12:ZnO	0	IST-EBV	-100	13,51182	-7,40093	7,59E-08	1
ZnO	40	Daudi	Fe12:ZnO	0	Daudi	-100	13,51182	-7,40093	7,59E-08	1
ZnO	40	Daudi	Fe12:ZnO	10	IST-EBV	-74,17965	14,24271	-5,20826	7,20E-04	1
ZnO	40	Daudi	Fe12:ZnO	10	Daudi	-47,53595	14,24271	-3,33756	0,57633	0
ZnO	40	Daudi	Fe12:ZnO	20	IST-EBV	-66,20226	14,24271	-4,64815	0,00621	1
ZnO	40	Daudi	Fe12:ZnO	20	Daudi	3,26E-13	14,24271	2,29E-14	1	0
ZnO	40	Daudi	Fe12:ZnO	30	IST-EBV	-51,1413	14,24271	-3,5907	0,25676	0
ZnO	40	Daudi	Fe12:ZnO	30	Daudi	-0,56189	14,24271	-0,03945	1	0
ZnO	40	Daudi	Fe12:ZnO	40	IST-EBV	-33,22457	14,24271	-2,33274	1	0
ZnO	40	Daudi	Fe12:ZnO	40	Daudi	-0,12433	14,24271	-0,00873	1	0
Fe6:ZnO	0	IST-EBV	Fe6:ZnO	0	Daudi	7,11E-14	11,03235	6,44E-15	1	0
Fe6:ZnO	0	IST-EBV	Fe6:ZnO	10	IST-EBV	16,6159	11,03235	1,50611	1	0
Fe6:ZnO	0	IST-EBV	Fe6:ZnO	10	Daudi	76,79787	11,03235	6,96115	5,05E-07	1
Fe6:ZnO	0	IST-EBV	Fe6:ZnO	20	IST-EBV	22,36416	11,03235	2,02714	1	0
Fe6:ZnO	0	IST-EBV	Fe6:ZnO	20	Daudi	100	11,03235	9,06425	5,47E-11	1
Fe6:ZnO	0	IST-EBV	Fe6:ZnO	30	IST-EBV	57,67203	11,03235	5,22754	6,67E-04	1
Fe6:ZnO	0	IST-EBV	Fe6:ZnO	30	Daudi	100	11,03235	9,06425	5,47E-11	1
Fe6:ZnO	0	IST-EBV	Fe6:ZnO	40	IST-EBV	89,24741	13,51182	6,60514	2,31E-06	1
Fe6:ZnO	0	IST-EBV	Fe6:ZnO	40	Daudi	100	13,51182	7,40093	7,59E-08	1
Fe6:ZnO	0	IST-EBV	Fe12:ZnO	0	IST-EBV	-5,68E-14	11,03235	-5,15E-15	1	0
Fe6:ZnO	0	IST-EBV	Fe12:ZnO	0	Daudi	-8,53E-14	11,03235	-7,73E-15	1	0
Fe6:ZnO	0	IST-EBV	Fe12:ZnO	10	IST-EBV	25,82035	11,9163	2,16681	1	0
Fe6:ZnO	0	IST-EBV	Fe12:ZnO	10	Daudi	52,46405	11,9163	4,40271	0,0154	1
Fe6:ZnO	0	IST-EBV	Fe12:ZnO	20	IST-EBV	33,79774	11,9163	2,83626	1	0
Fe6:ZnO	0	IST-EBV	Fe12:ZnO	20	Daudi	100	11,9163	8,39186	1,02E-09	1
Fe6:ZnO	0	IST-EBV	Fe12:ZnO	30	IST-EBV	48,8587	11,9163	4,10016	0,04557	1
Fe6:ZnO	0	IST-EBV	Fe12:ZnO	30	Daudi	99,43811	11,9163	8,34471	1,26E-09	1
Fe6:ZnO	0	IST-EBV	Fe12:ZnO	40	IST-EBV	66,77543	11,9163	5,6037	1,48E-04	1
Fe6:ZnO	0	IST-EBV	Fe12:ZnO	40	Daudi	99,87567	11,9163	8,38143	1,07E-09	1
Fe6:ZnO	0	Daudi	Fe6:ZnO	10	IST-EBV	16,6159	11,03235	1,50611	1	0

Fe6:ZnO	0	Daudi	Fe6:ZnO	10	Daudi	76,79787	11,03235	6,96115	5,05E-07	1
Fe6:ZnO	0	Daudi	Fe6:ZnO	20	IST-EBV	22,36416	11,03235	2,02714	1	0
Fe6:ZnO	0	Daudi	Fe6:ZnO	20	Daudi	100	11,03235	9,06425	5,47E-11	1
Fe6:ZnO	0	Daudi	Fe6:ZnO	30	IST-EBV	57,67203	11,03235	5,22754	6,67E-04	1
Fe6:ZnO	0	Daudi	Fe6:ZnO	30	Daudi	100	11,03235	9,06425	5,47E-11	1
Fe6:ZnO	0	Daudi	Fe6:ZnO	40	IST-EBV	89,24741	13,51182	6,60514	2,31E-06	1
Fe6:ZnO	0	Daudi	Fe6:ZnO	40	Daudi	100	13,51182	7,40093	7,59E-08	1
Fe6:ZnO	0	Daudi	Fe12:ZnO	0	IST-EBV	-1,28E-13	11,03235	-1,16E-14	1	0
Fe6:ZnO	0	Daudi	Fe12:ZnO	0	Daudi	-1,56E-13	11,03235	-1,42E-14	1	0
Fe6:ZnO	0	Daudi	Fe12:ZnO	10	IST-EBV	25,82035	11,9163	2,16681	1	0
Fe6:ZnO	0	Daudi	Fe12:ZnO	10	Daudi	52,46405	11,9163	4,40271	0,0154	1
Fe6:ZnO	0	Daudi	Fe12:ZnO	20	IST-EBV	33,79774	11,9163	2,83626	1	0
Fe6:ZnO	0	Daudi	Fe12:ZnO	20	Daudi	100	11,9163	8,39186	1,02E-09	1
Fe6:ZnO	0	Daudi	Fe12:ZnO	30	IST-EBV	48,8587	11,9163	4,10016	0,04557	1
Fe6:ZnO	0	Daudi	Fe12:ZnO	30	Daudi	99,43811	11,9163	8,34471	1,26E-09	1
Fe6:ZnO	0	Daudi	Fe12:ZnO	40	IST-EBV	66,77543	11,9163	5,6037	1,48E-04	1
Fe6:ZnO	0	Daudi	Fe12:ZnO	40	Daudi	99,87567	11,9163	8,38143	1,07E-09	1
Fe6:ZnO	10	IST-EBV	Fe6:ZnO	10	Daudi	60,18197	11,03235	5,45504	2,70E-04	1
Fe6:ZnO	10	IST-EBV	Fe6:ZnO	20	IST-EBV	5,74825	11,03235	0,52104	1	0
Fe6:ZnO	10	IST-EBV	Fe6:ZnO	20	Daudi	83,3841	11,03235	7,55814	3,84E-08	1
Fe6:ZnO	10	IST-EBV	Fe6:ZnO	30	IST-EBV	41,05613	11,03235	3,72143	0,16683	0
Fe6:ZnO	10	IST-EBV	Fe6:ZnO	30	Daudi	83,3841	11,03235	7,55814	3,84E-08	1
Fe6:ZnO	10	IST-EBV	Fe6:ZnO	40	IST-EBV	72,6315	13,51182	5,37541	3,71E-04	1
Fe6:ZnO	10	IST-EBV	Fe6:ZnO	40	Daudi	83,3841	13,51182	6,1712	1,43E-05	1
Fe6:ZnO	10	IST-EBV	Fe12:ZnO	0	IST-EBV	-16,6159	11,03235	-1,50611	1	0
Fe6:ZnO	10	IST-EBV	Fe12:ZnO	0	Daudi	-16,6159	11,03235	-1,50611	1	0
Fe6:ZnO	10	IST-EBV	Fe12:ZnO	10	IST-EBV	9,20444	11,9163	0,77242	1	0
Fe6:ZnO	10	IST-EBV	Fe12:ZnO	10	Daudi	35,84815	11,9163	3,00833	1	0
Fe6:ZnO	10	IST-EBV	Fe12:ZnO	20	IST-EBV	17,18183	11,9163	1,44188	1	0
Fe6:ZnO	10	IST-EBV	Fe12:ZnO	20	Daudi	83,3841	11,9163	6,99748	4,32E-07	1
Fe6:ZnO	10	IST-EBV	Fe12:ZnO	30	IST-EBV	32,2428	11,9163	2,70577	1	0
Fe6:ZnO	10	IST-EBV	Fe12:ZnO	30	Daudi	82,82221	11,9163	6,95033	5,29E-07	1
Fe6:ZnO	10	IST-EBV	Fe12:ZnO	40	IST-EBV	50,15953	11,9163	4,20932	0,03096	1
Fe6:ZnO	10	IST-EBV	Fe12:ZnO	40	Daudi	83,25977	11,9163	6,98705	4,52E-07	1
Fe6:ZnO	10	Daudi	Fe6:ZnO	20	IST-EBV	-54,43371	11,03235	-4,93401	0,0021	1
Fe6:ZnO	10	Daudi	Fe6:ZnO	20	Daudi	23,20213	11,03235	2,1031	1	0
Fe6:ZnO	10	Daudi	Fe6:ZnO	30	IST-EBV	-19,12584	11,03235	-1,73361	1	0
Fe6:ZnO	10	Daudi	Fe6:ZnO	30	Daudi	23,20213	11,03235	2,1031	1	0
Fe6:ZnO	10	Daudi	Fe6:ZnO	40	IST-EBV	12,44954	13,51182	0,92138	1	0
Fe6:ZnO	10	Daudi	Fe6:ZnO	40	Daudi	23,20213	13,51182	1,71717	1	0
Fe6:ZnO	10	Daudi	Fe12:ZnO	0	IST-EBV	-76,79787	11,03235	-6,96115	5,05E-07	1
Fe6:ZnO	10	Daudi	Fe12:ZnO	0	Daudi	-76,79787	11,03235	-6,96115	5,05E-07	1
Fe6:ZnO	10	Daudi	Fe12:ZnO	10	IST-EBV	-50,97752	11,9163	-4,27796	0,02421	1
Fe6:ZnO	10	Daudi	Fe12:ZnO	10	Daudi	-24,33381	11,9163	-2,04206	1	0
Fe6:ZnO	10	Daudi	Fe12:ZnO	20	IST-EBV	-43,00013	11,9163	-3,60851	0,24224	0
Fe6:ZnO	10	Daudi	Fe12:ZnO	20	Daudi	23,20213	11,9163	1,94709	1	0

Fe6:ZnO	10	Daudi	Fe12:ZnO	30	IST-EBV	-27,93916	11,9163	-2,34462	1	0
Fe6:ZnO	10	Daudi	Fe12:ZnO	30	Daudi	22,64024	11,9163	1,89994	1	0
Fe6:ZnO	10	Daudi	Fe12:ZnO	40	IST-EBV	-10,02243	11,9163	-0,84107	1	0
Fe6:ZnO	10	Daudi	Fe12:ZnO	40	Daudi	23,0778	11,9163	1,93666	1	0
Fe6:ZnO	20	IST-EBV	Fe6:ZnO	20	Daudi	77,63584	11,03235	7,03711	3,65E-07	1
Fe6:ZnO	20	IST-EBV	Fe6:ZnO	30	IST-EBV	35,30787	11,03235	3,20039	0,87976	0
Fe6:ZnO	20	IST-EBV	Fe6:ZnO	30	Daudi	77,63584	11,03235	7,03711	3,65E-07	1
Fe6:ZnO	20	IST-EBV	Fe6:ZnO	40	IST-EBV	66,88325	13,51182	4,94998	0,00197	1
Fe6:ZnO	20	IST-EBV	Fe6:ZnO	40	Daudi	77,63584	13,51182	5,74577	8,32E-05	1
Fe6:ZnO	20	IST-EBV	Fe12:ZnO	0	IST-EBV	-22,36416	11,03235	-2,02714	1	0
Fe6:ZnO	20	IST-EBV	Fe12:ZnO	0	Daudi	-22,36416	11,03235	-2,02714	1	0
Fe6:ZnO	20	IST-EBV	Fe12:ZnO	10	IST-EBV	3,45619	11,9163	0,29004	1	0
Fe6:ZnO	20	IST-EBV	Fe12:ZnO	10	Daudi	30,0999	11,9163	2,52594	1	0
Fe6:ZnO	20	IST-EBV	Fe12:ZnO	20	IST-EBV	11,43358	11,9163	0,95949	1	0
Fe6:ZnO	20	IST-EBV	Fe12:ZnO	20	Daudi	77,63584	11,9163	6,51509	3,38E-06	1
Fe6:ZnO	20	IST-EBV	Fe12:ZnO	30	IST-EBV	26,49455	11,9163	2,22339	1	0
Fe6:ZnO	20	IST-EBV	Fe12:ZnO	30	Daudi	77,07395	11,9163	6,46794	4,13E-06	1
Fe6:ZnO	20	IST-EBV	Fe12:ZnO	40	IST-EBV	44,41128	11,9163	3,72693	0,1638	0
Fe6:ZnO	20	IST-EBV	Fe12:ZnO	40	Daudi	77,51151	11,9163	6,50466	3,53E-06	1
Fe6:ZnO	20	Daudi	Fe6:ZnO	30	IST-EBV	-42,32797	11,03235	-3,83671	0,11323	0
Fe6:ZnO	20	Daudi	Fe6:ZnO	30	Daudi	7,90E-14	11,03235	7,17E-15	1	0
Fe6:ZnO	20	Daudi	Fe6:ZnO	40	IST-EBV	-10,75259	13,51182	-0,79579	1	0
Fe6:ZnO	20	Daudi	Fe6:ZnO	40	Daudi	1,05E-13	13,51182	7,79E-15	1	0
Fe6:ZnO	20	Daudi	Fe12:ZnO	0	IST-EBV	-100	11,03235	-9,06425	5,47E-11	1
Fe6:ZnO	20	Daudi	Fe12:ZnO	0	Daudi	-100	11,03235	-9,06425	5,47E-11	1
Fe6:ZnO	20	Daudi	Fe12:ZnO	10	IST-EBV	-74,17965	11,9163	-6,22506	1,15E-05	1
Fe6:ZnO	20	Daudi	Fe12:ZnO	10	Daudi	-47,53595	11,9163	-3,98915	0,06714	0
Fe6:ZnO	20	Daudi	Fe12:ZnO	20	IST-EBV	-66,20226	11,9163	-5,5556	1,80E-04	1
Fe6:ZnO	20	Daudi	Fe12:ZnO	20	Daudi	7,46E-14	11,9163	6,26E-15	1	0
Fe6:ZnO	20	Daudi	Fe12:ZnO	30	IST-EBV	-51,1413	11,9163	-4,29171	0,02304	1
Fe6:ZnO	20	Daudi	Fe12:ZnO	30	Daudi	-0,56189	11,9163	-0,04715	1	0
Fe6:ZnO	20	Daudi	Fe12:ZnO	40	IST-EBV	-33,22457	11,9163	-2,78816	1	0
Fe6:ZnO	20	Daudi	Fe12:ZnO	40	Daudi	-0,12433	11,9163	-0,01043	1	0
Fe6:ZnO	30	IST-EBV	Fe6:ZnO	30	Daudi	42,32797	11,03235	3,83671	0,11323	0
Fe6:ZnO	30	IST-EBV	Fe6:ZnO	40	IST-EBV	31,57538	13,51182	2,33687	1	0
Fe6:ZnO	30	IST-EBV	Fe6:ZnO	40	Daudi	42,32797	13,51182	3,13266	1	0
Fe6:ZnO	30	IST-EBV	Fe12:ZnO	0	IST-EBV	-57,67203	11,03235	-5,22754	6,67E-04	1
Fe6:ZnO	30	IST-EBV	Fe12:ZnO	0	Daudi	-57,67203	11,03235	-5,22754	6,67E-04	1
Fe6:ZnO	30	IST-EBV	Fe12:ZnO	10	IST-EBV	-31,85168	11,9163	-2,67295	1	0
Fe6:ZnO	30	IST-EBV	Fe12:ZnO	10	Daudi	-5,20797	11,9163	-0,43705	1	0
Fe6:ZnO	30	IST-EBV	Fe12:ZnO	20	IST-EBV	-23,87429	11,9163	-2,0035	1	0
Fe6:ZnO	30	IST-EBV	Fe12:ZnO	20	Daudi	42,32797	11,9163	3,55211	0,29111	0
Fe6:ZnO	30	IST-EBV	Fe12:ZnO	30	IST-EBV	-8,81332	11,9163	-0,7396	1	0
Fe6:ZnO	30	IST-EBV	Fe12:ZnO	30	Daudi	41,76608	11,9163	3,50495	0,339	0
Fe6:ZnO	30	IST-EBV	Fe12:ZnO	40	IST-EBV	9,10341	11,9163	0,76395	1	0
Fe6:ZnO	30	IST-EBV	Fe12:ZnO	40	Daudi	42,20364	11,9163	3,54167	0,30112	0

Fe6:ZnO	30	Daudi	Fe6:ZnO	40	IST-EBV	-10,75259	13,51182	-0,79579	1	0
Fe6:ZnO	30	Daudi	Fe6:ZnO	40	Daudi	2,62E-14	13,51182	1,94E-15	1	0
Fe6:ZnO	30	Daudi	Fe12:ZnO	0	IST-EBV	-100	11,03235	-9,06425	5,47E-11	1
Fe6:ZnO	30	Daudi	Fe12:ZnO	0	Daudi	-100	11,03235	-9,06425	5,47E-11	1
Fe6:ZnO	30	Daudi	Fe12:ZnO	10	IST-EBV	-74,17965	11,9163	-6,22506	1,15E-05	1
Fe6:ZnO	30	Daudi	Fe12:ZnO	10	Daudi	-47,53595	11,9163	-3,98915	0,06714	0
Fe6:ZnO	30	Daudi	Fe12:ZnO	20	IST-EBV	-66,20226	11,9163	-5,5556	1,80E-04	1
Fe6:ZnO	30	Daudi	Fe12:ZnO	20	Daudi	-4,44E-15	11,9163	-3,73E-16	1	0
Fe6:ZnO	30	Daudi	Fe12:ZnO	30	IST-EBV	-51,1413	11,9163	-4,29171	0,02304	1
Fe6:ZnO	30	Daudi	Fe12:ZnO	30	Daudi	-0,56189	11,9163	-0,04715	1	0
Fe6:ZnO	30	Daudi	Fe12:ZnO	40	IST-EBV	-33,22457	11,9163	-2,78816	1	0
Fe6:ZnO	30	Daudi	Fe12:ZnO	40	Daudi	-0,12433	11,9163	-0,01043	1	0
Fe6:ZnO	40	IST-EBV	Fe6:ZnO	40	Daudi	10,75259	15,6021	0,68918	1	0
Fe6:ZnO	40	IST-EBV	Fe12:ZnO	0	IST-EBV	-89,24741	13,51182	-6,60514	2,31E-06	1
Fe6:ZnO	40	IST-EBV	Fe12:ZnO	0	Daudi	-89,24741	13,51182	-6,60514	2,31E-06	1
Fe6:ZnO	40	IST-EBV	Fe12:ZnO	10	IST-EBV	-63,42706	14,24271	-4,4533	0,0128	1
Fe6:ZnO	40	IST-EBV	Fe12:ZnO	10	Daudi	-36,78335	14,24271	-2,58261	1	0
Fe6:ZnO	40	IST-EBV	Fe12:ZnO	20	IST-EBV	-55,44967	14,24271	-3,8932	0,09342	0
Fe6:ZnO	40	IST-EBV	Fe12:ZnO	20	Daudi	10,75259	14,24271	0,75495	1	0
Fe6:ZnO	40	IST-EBV	Fe12:ZnO	30	IST-EBV	-40,3887	14,24271	-2,83575	1	0
Fe6:ZnO	40	IST-EBV	Fe12:ZnO	30	Daudi	10,1907	14,24271	0,7155	1	0
Fe6:ZnO	40	IST-EBV	Fe12:ZnO	40	IST-EBV	-22,47197	14,24271	-1,57779	1	0
Fe6:ZnO	40	IST-EBV	Fe12:ZnO	40	Daudi	10,62826	14,24271	0,74622	1	0
Fe6:ZnO	40	Daudi	Fe12:ZnO	0	IST-EBV	-100	13,51182	-7,40093	7,59E-08	1
Fe6:ZnO	40	Daudi	Fe12:ZnO	0	Daudi	-100	13,51182	-7,40093	7,59E-08	1
Fe6:ZnO	40	Daudi	Fe12:ZnO	10	IST-EBV	-74,17965	14,24271	-5,20826	7,20E-04	1
Fe6:ZnO	40	Daudi	Fe12:ZnO	10	Daudi	-47,53595	14,24271	-3,33756	0,57633	0
Fe6:ZnO	40	Daudi	Fe12:ZnO	20	IST-EBV	-66,20226	14,24271	-4,64815	0,00621	1
Fe6:ZnO	40	Daudi	Fe12:ZnO	20	Daudi	-3,06E-14	14,24271	-2,15E-15	1	0
Fe6:ZnO	40	Daudi	Fe12:ZnO	30	IST-EBV	-51,1413	14,24271	-3,5907	0,25676	0
Fe6:ZnO	40	Daudi	Fe12:ZnO	30	Daudi	-0,56189	14,24271	-0,03945	1	0
Fe6:ZnO	40	Daudi	Fe12:ZnO	40	IST-EBV	-33,22457	14,24271	-2,33274	1	0
Fe6:ZnO	40	Daudi	Fe12:ZnO	40	Daudi	-0,12433	14,24271	-0,00873	1	0
Fe12:ZnO	0	IST-EBV	Fe12:ZnO	0	Daudi	-2,84E-14	11,03235	-2,58E-15	1	0
Fe12:ZnO	0	IST-EBV	Fe12:ZnO	10	IST-EBV	25,82035	11,9163	2,16681	1	0
Fe12:ZnO	0	IST-EBV	Fe12:ZnO	10	Daudi	52,46405	11,9163	4,40271	0,0154	1
Fe12:ZnO	0	IST-EBV	Fe12:ZnO	20	IST-EBV	33,79774	11,9163	2,83626	1	0
Fe12:ZnO	0	IST-EBV	Fe12:ZnO	20	Daudi	100	11,9163	8,39186	1,02E-09	1
Fe12:ZnO	0	IST-EBV	Fe12:ZnO	30	IST-EBV	48,8587	11,9163	4,10016	0,04557	1
Fe12:ZnO	0	IST-EBV	Fe12:ZnO	30	Daudi	99,43811	11,9163	8,34471	1,26E-09	1
Fe12:ZnO	0	IST-EBV	Fe12:ZnO	40	IST-EBV	66,77543	11,9163	5,6037	1,48E-04	1
Fe12:ZnO	0	IST-EBV	Fe12:ZnO	40	Daudi	99,87567	11,9163	8,38143	1,07E-09	1
Fe12:ZnO	0	Daudi	Fe12:ZnO	10	IST-EBV	25,82035	11,9163	2,16681	1	0
Fe12:ZnO	0	Daudi	Fe12:ZnO	10	Daudi	52,46405	11,9163	4,40271	0,0154	1
Fe12:ZnO	0	Daudi	Fe12:ZnO	20	IST-EBV	33,79774	11,9163	2,83626	1	0
Fe12:ZnO	0	Daudi	Fe12:ZnO	20	Daudi	100	11,9163	8,39186	1,02E-09	1

Fe12:ZnO	0	Daudi	Fe12:ZnO	30	IST-EBV	48,8587	11,9163	4,10016	0,04557	1
Fe12:ZnO	0	Daudi	Fe12:ZnO	30	Daudi	99,43811	11,9163	8,34471	1,26E-09	1
Fe12:ZnO	0	Daudi	Fe12:ZnO	40	IST-EBV	66,77543	11,9163	5,6037	1,48E-04	1
Fe12:ZnO	0	Daudi	Fe12:ZnO	40	Daudi	99,87567	11,9163	8,38143	1,07E-09	1
Fe12:ZnO	10	IST-EBV	Fe12:ZnO	10	Daudi	26,64371	12,73906	2,0915	1	0
Fe12:ZnO	10	IST-EBV	Fe12:ZnO	20	IST-EBV	7,97739	12,73906	0,62621	1	0
Fe12:ZnO	10	IST-EBV	Fe12:ZnO	20	Daudi	74,17965	12,73906	5,82301	6,06E-05	1
Fe12:ZnO	10	IST-EBV	Fe12:ZnO	30	IST-EBV	23,03836	12,73906	1,80848	1	0
Fe12:ZnO	10	IST-EBV	Fe12:ZnO	30	Daudi	73,61776	12,73906	5,7789	7,26E-05	1
Fe12:ZnO	10	IST-EBV	Fe12:ZnO	40	IST-EBV	40,95509	12,73906	3,21492	0,84165	0
Fe12:ZnO	10	IST-EBV	Fe12:ZnO	40	Daudi	74,05532	12,73906	5,81325	6,31E-05	1
Fe12:ZnO	10	Daudi	Fe12:ZnO	20	IST-EBV	-18,66632	12,73906	-1,46528	1	0
Fe12:ZnO	10	Daudi	Fe12:ZnO	20	Daudi	47,53595	12,73906	3,73151	0,16132	0
Fe12:ZnO	10	Daudi	Fe12:ZnO	30	IST-EBV	-3,60535	12,73906	-0,28302	1	0
Fe12:ZnO	10	Daudi	Fe12:ZnO	30	Daudi	46,97405	12,73906	3,6874	0,18681	0
Fe12:ZnO	10	Daudi	Fe12:ZnO	40	IST-EBV	14,31138	12,73906	1,12342	1	0
Fe12:ZnO	10	Daudi	Fe12:ZnO	40	Daudi	47,41161	12,73906	3,72175	0,16666	0
Fe12:ZnO	20	IST-EBV	Fe12:ZnO	20	Daudi	66,20226	12,73906	5,19679	7,53E-04	1
Fe12:ZnO	20	IST-EBV	Fe12:ZnO	30	IST-EBV	15,06097	12,73906	1,18227	1	0
Fe12:ZnO	20	IST-EBV	Fe12:ZnO	30	Daudi	65,64037	12,73906	5,15268	8,96E-04	1
Fe12:ZnO	20	IST-EBV	Fe12:ZnO	40	IST-EBV	32,9777	12,73906	2,58871	1	0
Fe12:ZnO	20	IST-EBV	Fe12:ZnO	40	Daudi	66,07793	12,73906	5,18703	7,83E-04	1
Fe12:ZnO	20	Daudi	Fe12:ZnO	30	IST-EBV	-51,1413	12,73906	-4,01453	0,06148	0
Fe12:ZnO	20	Daudi	Fe12:ZnO	30	Daudi	-0,56189	12,73906	-0,04411	1	0
Fe12:ZnO	20	Daudi	Fe12:ZnO	40	IST-EBV	-33,22457	12,73906	-2,60809	1	0
Fe12:ZnO	20	Daudi	Fe12:ZnO	40	Daudi	-0,12433	12,73906	-0,00976	1	0
Fe12:ZnO	30	IST-EBV	Fe12:ZnO	30	Daudi	50,57941	12,73906	3,97042	0,07164	0
Fe12:ZnO	30	IST-EBV	Fe12:ZnO	40	IST-EBV	17,91673	12,73906	1,40644	1	0
Fe12:ZnO	30	IST-EBV	Fe12:ZnO	40	Daudi	51,01696	12,73906	4,00477	0,0636	0
Fe12:ZnO	30	Daudi	Fe12:ZnO	40	IST-EBV	-32,66268	12,73906	-2,56398	1	0
Fe12:ZnO	30	Daudi	Fe12:ZnO	40	Daudi	0,43756	12,73906	0,03435	1	0
Fe12:ZnO	40	IST-EBV	Fe12:ZnO	40	Daudi	33,10023	12,73906	2,59833	1	0

Three way ANOVA analysis on ZnO and Fe:ZnO NPs uptake experiments on B lymphocytes cell line(Bonferroni t-test).

**Comparison between factor: Nanoparticle**						
		**Mean Diff.**	**SEM**	**t Value**	**Prob**	**Sig**
ZnO	Fe6:ZnO	-15,94	6,68932	-2,3829	0,07633	0
ZnO	Fe12:ZnO	-24,33333	6,68932	-3,63764	0,00393	1
Fe6:ZnO	Fe12:ZnO	-8,39333	6,68932	-1,25474	0,66497	0

**Comparison between factor: Incubation time**						
		**Mean Diff.**	**SEM**	**t Value**	**Prob**	**Sig**
5 h	24 h	24,48667	5,4618	4,48326	0,000155	1

Comparison between factor: Dose (µg/mL)						
		Mean Diff.	SEM	t Value	Prob	Sig
10	20	-27,96	5,4618	-5,11919	3,07E-05	1

All pairwise multiple comparisons										
NP	Incubation	Dose (µg/mL)	NP	Incubation	Dose (µg/mL)	MeanDiff	SEM	t Value	Prob	Sig
ZnO	5h	--	ZnO	24h	--	20,92667	9,46012	2,21209	0,55091	0
ZnO	5h	--	Fe6:ZnO	5h	--	-22,78333	9,46012	-2,40836	0,36098	0
ZnO	5h	--	Fe6:ZnO	24h	--	11,83	9,46012	1,25051	1	0
ZnO	5h	--	Fe12:ZnO	5h	--	-22,83	9,46012	-2,41329	0,3571	0
ZnO	5h	--	Fe12:ZnO	24h	--	-4,91	9,46012	-0,51902	1	0
ZnO	24h	--	Fe6:ZnO	5h	--	-43,71	9,46012	-4,62045	0,00164	1
ZnO	24h	--	Fe6:ZnO	24h	--	-9,09667	9,46012	-0,96158	1	0
ZnO	24h	--	Fe12:ZnO	5h	--	-43,75667	9,46012	-4,62538	0,00161	1
ZnO	24h	--	Fe12:ZnO	24h	--	-25,83667	9,46012	-2,73111	0,17466	0
Fe6:ZnO	5h	--	Fe6:ZnO	24h	--	34,61333	9,46012	3,65887	0,01862	1
Fe6:ZnO	5h	--	Fe12:ZnO	5h	--	-0,04667	9,46012	-0,00493	1	0
Fe6:ZnO	5h	--	Fe12:ZnO	24h	--	17,87333	9,46012	1,88933	1	0
Fe6:ZnO	24h	--	Fe12:ZnO	5h	--	-34,66	9,46012	-3,6638	0,01839	1
Fe6:ZnO	24h	--	Fe12:ZnO	24h	--	-16,74	9,46012	-1,76953	1	0
Fe12:ZnO	5h	--	Fe12:ZnO	24h	--	17,92	9,46012	1,89427	1	0
ZnO	--	10	ZnO	--	20	-23,63	9,46012	-2,49785	0,29622	0
ZnO	--	10	Fe6:ZnO	--	10	-14,44167	9,46012	-1,52658	1	0
ZnO	--	10	Fe6:ZnO	--	20	-41,06833	9,46012	-4,34121	0,00333	1
ZnO	--	10	Fe12:ZnO	--	10	-19,33667	9,46012	-2,04402	0,78111	0
ZnO	--	10	Fe12:ZnO	--	20	-52,96	9,46012	-5,59824	1,38E-04	1
ZnO	--	20	Fe6:ZnO	--	10	9,18833	9,46012	0,97127	1	0
ZnO	--	20	Fe6:ZnO	--	20	-17,43833	9,46012	-1,84335	1	0
ZnO	--	20	Fe12:ZnO	--	10	4,29333	9,46012	0,45383	1	0
ZnO	--	20	Fe12:ZnO	--	20	-29,33	9,46012	-3,10038	0,07325	0
Fe6:ZnO	--	10	Fe6:ZnO	--	20	-26,62667	9,46012	-2,81462	0,14396	0
Fe6:ZnO	--	10	Fe12:ZnO	--	10	-4,895	9,46012	-0,51744	1	0
Fe6:ZnO	--	10	Fe12:ZnO	--	20	-38,51833	9,46012	-4,07165	0,00659	1
Fe6:ZnO	--	20	Fe12:ZnO	--	10	21,73167	9,46012	2,29719	0,45951	0
Fe6:ZnO	--	20	Fe12:ZnO	--	20	-11,89167	9,46012	-1,25703	1	0
Fe12:ZnO	--	10	Fe12:ZnO	--	20	-33,62333	9,46012	-3,55422	0,02416	1
--	5h	10	--	5h	20	-22,89	7,72416	-2,96343	0,04061	1
--	5h	10	--	24h	10	29,55667	7,72416	3,82652	0,0049	1
--	5h	10	--	24h	20	-3,47333	7,72416	-0,44967	1	0
--	5h	20	--	24h	10	52,44667	7,72416	6,78995	3,03E-06	1
--	5h	20	--	24h	20	19,41667	7,72416	2,51376	0,11436	0
--	24h	10	--	24h	20	-33,03	7,72416	-4,27619	0,00157	1
ZnO	5h	10	ZnO	5h	20	-14,77667	13,37863	-1,1045	1	0

ZnO	5h	10	ZnO	24h	10	29,78	13,37863	2,22594	1	0
ZnO	5h	10	ZnO	24h	20	-2,70333	13,37863	-0,20206	1	0
ZnO	5h	10	Fe6:ZnO	5h	10	-19,12	13,37863	-1,42914	1	0
ZnO	5h	10	Fe6:ZnO	5h	20	-41,22333	13,37863	-3,08128	0,33738	0
ZnO	5h	10	Fe6:ZnO	24h	10	20,01667	13,37863	1,49617	1	0
ZnO	5h	10	Fe6:ZnO	24h	20	-11,13333	13,37863	-0,83217	1	0
ZnO	5h	10	Fe12:ZnO	5h	10	-14,32333	13,37863	-1,07061	1	0
ZnO	5h	10	Fe12:ZnO	5h	20	-46,11333	13,37863	-3,44679	0,13867	0
ZnO	5h	10	Fe12:ZnO	24h	10	5,43	13,37863	0,40587	1	0
ZnO	5h	10	Fe12:ZnO	24h	20	-30,02667	13,37863	-2,24437	1	0
ZnO	5h	20	ZnO	24h	10	44,55667	13,37863	3,33043	0,18454	0
ZnO	5h	20	ZnO	24h	20	12,07333	13,37863	0,90243	1	0
ZnO	5h	20	Fe6:ZnO	5h	10	-4,34333	13,37863	-0,32465	1	0
ZnO	5h	20	Fe6:ZnO	5h	20	-26,44667	13,37863	-1,97678	1	0
ZnO	5h	20	Fe6:ZnO	24h	10	34,79333	13,37863	2,60066	1	0
ZnO	5h	20	Fe6:ZnO	24h	20	3,64333	13,37863	0,27232	1	0
ZnO	5h	20	Fe12:ZnO	5h	10	0,45333	13,37863	0,03388	1	0
ZnO	5h	20	Fe12:ZnO	5h	20	-31,33667	13,37863	-2,34229	1	0
ZnO	5h	20	Fe12:ZnO	24h	10	20,20667	13,37863	1,51037	1	0
ZnO	5h	20	Fe12:ZnO	24h	20	-15,25	13,37863	-1,13988	1	0
ZnO	24h	10	ZnO	24h	20	-32,48333	13,37863	-2,428	1	0
ZnO	24h	10	Fe6:ZnO	5h	10	-48,9	13,37863	-3,65508	0,08272	0
ZnO	24h	10	Fe6:ZnO	5h	20	-71,00333	13,37863	-5,30722	0,00126	1
ZnO	24h	10	Fe6:ZnO	24h	10	-9,76333	13,37863	-0,72977	1	0
ZnO	24h	10	Fe6:ZnO	24h	20	-40,91333	13,37863	-3,05811	0,35661	0
ZnO	24h	10	Fe12:ZnO	5h	10	-44,10333	13,37863	-3,29655	0,20047	0
ZnO	24h	10	Fe12:ZnO	5h	20	-75,89333	13,37863	-5,67273	5,05E-04	1
ZnO	24h	10	Fe12:ZnO	24h	10	-24,35	13,37863	-1,82007	1	0
ZnO	24h	10	Fe12:ZnO	24h	20	-59,80667	13,37863	-4,47031	0,01054	1
ZnO	24h	20	Fe6:ZnO	5h	10	-16,41667	13,37863	-1,22708	1	0
ZnO	24h	20	Fe6:ZnO	5h	20	-38,52	13,37863	-2,87922	0,54471	0
ZnO	24h	20	Fe6:ZnO	24h	10	22,72	13,37863	1,69823	1	0
ZnO	24h	20	Fe6:ZnO	24h	20	-8,43	13,37863	-0,63011	1	0
ZnO	24h	20	Fe12:ZnO	5h	10	-11,62	13,37863	-0,86855	1	0
ZnO	24h	20	Fe12:ZnO	5h	20	-43,41	13,37863	-3,24473	0,22743	0
ZnO	24h	20	Fe12:ZnO	24h	10	8,13333	13,37863	0,60793	1	0
ZnO	24h	20	Fe12:ZnO	24h	20	-27,32333	13,37863	-2,04231	1	0
Fe6:ZnO	5h	10	Fe6:ZnO	5h	20	-22,10333	13,37863	-1,65214	1	0
Fe6:ZnO	5h	10	Fe6:ZnO	24h	10	39,13667	13,37863	2,92531	0,48877	0
Fe6:ZnO	5h	10	Fe6:ZnO	24h	20	7,98667	13,37863	0,59697	1	0
Fe6:ZnO	5h	10	Fe12:ZnO	5h	10	4,79667	13,37863	0,35853	1	0
Fe6:ZnO	5h	10	Fe12:ZnO	5h	20	-26,99333	13,37863	-2,01765	1	0
Fe6:ZnO	5h	10	Fe12:ZnO	24h	10	24,55	13,37863	1,83502	1	0
Fe6:ZnO	5h	10	Fe12:ZnO	24h	20	-10,90667	13,37863	-0,81523	1	0
Fe6:ZnO	5h	20	Fe6:ZnO	24h	10	61,24	13,37863	4,57745	0,00803	1
Fe6:ZnO	5h	20	Fe6:ZnO	24h	20	30,09	13,37863	2,24911	1	0

Fe6:ZnO	5h	20	Fe12:ZnO	5h	10	26,9	13,37863	2,01067	1	0
Fe6:ZnO	5h	20	Fe12:ZnO	5h	20	-4,89	13,37863	-0,36551	1	0
Fe6:ZnO	5h	20	Fe12:ZnO	24h	10	46,65333	13,37863	3,48715	0,12552	0
Fe6:ZnO	5h	20	Fe12:ZnO	24h	20	11,19667	13,37863	0,83691	1	0
Fe6:ZnO	24h	10	Fe6:ZnO	24h	20	-31,15	13,37863	-2,32834	1	0
Fe6:ZnO	24h	10	Fe12:ZnO	5h	10	-34,34	13,37863	-2,56678	1	0
Fe6:ZnO	24h	10	Fe12:ZnO	5h	20	-66,13	13,37863	-4,94296	0,00317	1
Fe6:ZnO	24h	10	Fe12:ZnO	24h	10	-14,58667	13,37863	-1,0903	1	0
Fe6:ZnO	24h	10	Fe12:ZnO	24h	20	-50,04333	13,37863	-3,74054	0,06681	0
Fe6:ZnO	24h	20	Fe12:ZnO	5h	10	-3,19	13,37863	-0,23844	1	0
Fe6:ZnO	24h	20	Fe12:ZnO	5h	20	-34,98	13,37863	-2,61462	1	0
Fe6:ZnO	24h	20	Fe12:ZnO	24h	10	16,56333	13,37863	1,23804	1	0
Fe6:ZnO	24h	20	Fe12:ZnO	24h	20	-18,89333	13,37863	-1,4122	1	0
Fe12:ZnO	5h	10	Fe12:ZnO	5h	20	-31,79	13,37863	-2,37618	1	0
Fe12:ZnO	5h	10	Fe12:ZnO	24h	10	19,75333	13,37863	1,47648	1	0
Fe12:ZnO	5h	10	Fe12:ZnO	24h	20	-15,70333	13,37863	-1,17376	1	0
Fe12:ZnO	5h	20	Fe12:ZnO	24h	10	51,54333	13,37863	3,85266	0,05042	0
Fe12:ZnO	5h	20	Fe12:ZnO	24h	20	16,08667	13,37863	1,20241	1	0
Fe12:ZnO	24h	10	Fe12:ZnO	24h	20	-35,45667	13,37863	-2,65025	0,92482	0

Three way ANOVA analysis on ZnO and Fe:ZnO NPs uptake experiments on Daudi cell line(Bonferroni t-test).

**Comparison between factor: Nanoparticle**						
		**Mean Diff.**	**SEM**	**t Value**	**Prob**	**Sig**
ZnO	Fe6:ZnO	-11,90667	4,73687	-2,51361	0,05931	0
ZnO	Fe12:ZnO	-7,07958	5,02421	-1,40909	0,51836	0
Fe6:ZnO	Fe12:ZnO	4,82708	5,02421	0,96076	1	0

**Comparison between factor: Incubation time**						
		**Mean Diff.**	**SEM**	**t Value**	**Prob**	**Sig**
5 h	24 h	52,14972	4,02557	12,95462	9,02E-12	1

**Comparison between factor: Dose (µg/mL)**						
		**Mean Diff.**	**SEM**	**t Value**	**Prob**	**Sig**
10	20	-6,63083	4,02557	-1,64718	0,11373	0

**All pairwise multiple comparisons**										
**NP**	**Incubation**	**Dose (µg/mL)**	**NP**	**Incubation**	**Dose (µg/mL)**	**MeanDiff**	**SEM**	**t Value**	**Prob**	**Sig**
ZnO	5h	--	ZnO	24h	--	41,205	6,69895	6,15096	5,15E-05	1
ZnO	5h	--	Fe6:ZnO	5h	--	-23,68667	6,69895	-3,53588	0,02786	1
ZnO	5h	--	Fe6:ZnO	24h	--	41,07833	6,69895	6,13205	5,38E-05	1
ZnO	5h	--	Fe12:ZnO	5h	--	-11,71667	6,69895	-1,74903	1	0
ZnO	5h	--	Fe12:ZnO	24h	--	38,7625	7,48966	5,17547	5,17E-04	1

ZnO	24h	--	Fe6:ZnO	5h	--	-64,89167	6,69895	-9,68684	3,22E-08	1
ZnO	24h	--	Fe6:ZnO	24h	--	-0,12667	6,69895	-0,01891	1	0
ZnO	24h	--	Fe12:ZnO	5h	--	-52,92167	6,69895	-7,89999	1,09E-06	1
ZnO	24h	--	Fe12:ZnO	24h	--	-2,4425	7,48966	-0,32612	1	0
Fe6:ZnO	5h	--	Fe6:ZnO	24h	--	64,765	6,69895	9,66793	3,34E-08	1
Fe6:ZnO	5h	--	Fe12:ZnO	5h	--	11,97	6,69895	1,78685	1	0
Fe6:ZnO	5h	--	Fe12:ZnO	24h	--	62,44917	7,48966	8,33805	4,42E-07	1
Fe6:ZnO	24h	--	Fe12:ZnO	5h	--	-52,795	6,69895	-7,88108	1,13E-06	1
Fe6:ZnO	24h	--	Fe12:ZnO	24h	--	-2,31583	7,48966	-0,3092	1	0
Fe12:ZnO	5h	--	Fe12:ZnO	24h	--	50,47917	7,48966	6,73985	1,35E-05	1
ZnO	--	10	ZnO	--	20	-8,715	6,69895	-1,30095	1	0
ZnO	--	10	Fe6:ZnO	--	10	-12,42833	6,69895	-1,85527	1	0
ZnO	--	10	Fe6:ZnO	--	20	-20,1	6,69895	-3,00047	0,09881	0
ZnO	--	10	Fe12:ZnO	--	10	-9,68417	7,10531	-1,36295	1	0
ZnO	--	10	Fe12:ZnO	--	20	-13,19	7,10531	-1,85636	1,00E+00	0
ZnO	--	20	Fe6:ZnO	--	10	-3,71333	6,69895	-0,55432	1	0
ZnO	--	20	Fe6:ZnO	--	20	-11,385	6,69895	-1,69952	1	0
ZnO	--	20	Fe12:ZnO	--	10	-0,96917	7,10531	-0,1364	1	0
ZnO	--	20	Fe12:ZnO	--	20	-4,475	7,10531	-0,62981	1	0
Fe6:ZnO	--	10	Fe6:ZnO	--	20	-7,67167	6,69895	-1,1452	1	0
Fe6:ZnO	--	10	Fe12:ZnO	--	10	2,74417	7,10531	0,38621	1	0
Fe6:ZnO	--	10	Fe12:ZnO	--	20	-0,76167	7,10531	-0,1072	1	0
Fe6:ZnO	--	20	Fe12:ZnO	--	10	10,41583	7,10531	1,46592	1	0
Fe6:ZnO	--	20	Fe12:ZnO	--	20	6,91	7,10531	0,97251	1	0
Fe12:ZnO	--	10	Fe12:ZnO	--	20	-3,50583	7,48966	-0,46809	1	0
--	5h	10	--	5h	20	-28,47444	5,46967	-5,20588	1,92E-04	1
--	5h	10	--	24h	10	30,30611	5,69301	5,32339	1,45E-04	1
--	5h	10	--	24h	20	45,51889	5,69301	7,99557	3,57E-07	1
--	5h	20	--	24h	10	58,78056	5,69301	10,32503	4,03E-09	1
--	5h	20	--	24h	20	73,99333	5,69301	12,99721	5,07E-11	1
--	24h	10	--	24h	20	15,21278	5,90792	2,57498	0,10365	0
ZnO	5h	10	ZnO	5h	20	-30,93	9,47375	-3,26481	0,23402	0
ZnO	5h	10	ZnO	24h	10	18,99	9,47375	2,00449	1	0
ZnO	5h	10	ZnO	24h	20	32,49	9,47375	3,42948	0,15817	0
ZnO	5h	10	Fe6:ZnO	5h	10	-24,60333	9,47375	-2,597	1	0
ZnO	5h	10	Fe6:ZnO	5h	20	-53,7	9,47375	-5,66829	7,01E-04	1
ZnO	5h	10	Fe6:ZnO	24h	10	18,73667	9,47375	1,97775	1	0
ZnO	5h	10	Fe6:ZnO	24h	20	32,49	9,47375	3,42948	0,15817	0
ZnO	5h	10	Fe12:ZnO	5h	10	-14,48333	9,47375	-1,52879	1	0
ZnO	5h	10	Fe12:ZnO	5h	20	-39,88	9,47375	-4,20953	0,02387	1
ZnO	5h	10	Fe12:ZnO	24h	10	14,105	10,59197	1,33167	1	0
ZnO	5h	10	Fe12:ZnO	24h	20	32,49	10,59197	3,06742	0,37211	0
ZnO	5h	20	ZnO	24h	10	49,92	9,47375	5,2693	0,00182	1
ZnO	5h	20	ZnO	24h	20	63,42	9,47375	6,69429	6,56E-05	1
ZnO	5h	20	Fe6:ZnO	5h	10	6,32667	9,47375	0,66781	1	0
ZnO	5h	20	Fe6:ZnO	5h	20	-22,77	9,47375	-2,40348	1	0

ZnO	5h	20	Fe6:ZnO	24h	10	49,66667	9,47375	5,24256	0,00194	1
ZnO	5h	20	Fe6:ZnO	24h	20	63,42	9,47375	6,69429	6,56E-05	1
ZnO	5h	20	Fe12:ZnO	5h	10	16,44667	9,47375	1,73603	1	0
ZnO	5h	20	Fe12:ZnO	5h	20	-8,95	9,47375	-0,94472	1	0
ZnO	5h	20	Fe12:ZnO	24h	10	45,035	10,59197	4,2518	0,02153	1
ZnO	5h	20	Fe12:ZnO	24h	20	63,42	10,59197	5,98755	3,31E-04	1
ZnO	24h	10	ZnO	24h	20	13,5	9,47375	1,42499	1	0
ZnO	24h	10	Fe6:ZnO	5h	10	-43,59333	9,47375	-4,60149	0,00916	1
ZnO	24h	10	Fe6:ZnO	5h	20	-72,69	9,47375	-7,67278	7,74E-06	1
ZnO	24h	10	Fe6:ZnO	24h	10	-0,25333	9,47375	-0,02674	1	0
ZnO	24h	10	Fe6:ZnO	24h	20	13,5	9,47375	1,42499	1	0
ZnO	24h	10	Fe12:ZnO	5h	10	-33,47333	9,47375	-3,53327	0,12333	0
ZnO	24h	10	Fe12:ZnO	5h	20	-58,87	9,47375	-6,21401	1,96E-04	1
ZnO	24h	10	Fe12:ZnO	24h	10	-4,885	10,59197	-0,4612	1	0
ZnO	24h	10	Fe12:ZnO	24h	20	13,5	10,59197	1,27455	1	0
ZnO	24h	20	Fe6:ZnO	5h	10	-57,09333	9,47375	-6,02648	3,02E-04	1
ZnO	24h	20	Fe6:ZnO	5h	20	-86,19	9,47375	-9,09777	4,32E-07	1
ZnO	24h	20	Fe6:ZnO	24h	10	-13,75333	9,47375	-1,45173	1	0
ZnO	24h	20	Fe6:ZnO	24h	20	1,82E-14	9,47375	1,92E-15	1	0
ZnO	24h	20	Fe12:ZnO	5h	10	-46,97333	9,47375	-4,95826	0,00385	1
ZnO	24h	20	Fe12:ZnO	5h	20	-72,37	9,47375	-7,639	8,31E-06	1
ZnO	24h	20	Fe12:ZnO	24h	10	-18,385	10,59197	-1,73575	1	0
ZnO	24h	20	Fe12:ZnO	24h	20	4,00E-15	10,59197	3,77E-16	1	0
Fe6:ZnO	5h	10	Fe6:ZnO	5h	20	-29,09667	9,47375	-3,07129	0,36876	0
Fe6:ZnO	5h	10	Fe6:ZnO	24h	10	43,34	9,47375	4,57475	0,00978	1
Fe6:ZnO	5h	10	Fe6:ZnO	24h	20	57,09333	9,47375	6,02648	3,02E-04	1
Fe6:ZnO	5h	10	Fe12:ZnO	5h	10	10,12	9,47375	1,06821	1	0
Fe6:ZnO	5h	10	Fe12:ZnO	5h	20	-15,27667	9,47375	-1,61253	1	0
Fe6:ZnO	5h	10	Fe12:ZnO	24h	10	38,70833	10,59197	3,6545	0,0921	0
Fe6:ZnO	5h	10	Fe12:ZnO	24h	20	57,09333	10,59197	5,39024	0,00136	1
Fe6:ZnO	5h	20	Fe6:ZnO	24h	10	72,43667	9,47375	7,64604	8,19E-06	1
Fe6:ZnO	5h	20	Fe6:ZnO	24h	20	86,19	9,47375	9,09777	4,32E-07	1
Fe6:ZnO	5h	20	Fe12:ZnO	5h	10	39,21667	9,47375	4,13951	0,02832	1
Fe6:ZnO	5h	20	Fe12:ZnO	5h	20	13,82	9,47375	1,45877	1	0
Fe6:ZnO	5h	20	Fe12:ZnO	24h	10	67,805	10,59197	6,40155	1,27E-04	1
Fe6:ZnO	5h	20	Fe12:ZnO	24h	20	86,19	10,59197	8,13729	2,93E-06	1
Fe6:ZnO	24h	10	Fe6:ZnO	24h	20	13,75333	9,47375	1,45173	1	0
Fe6:ZnO	24h	10	Fe12:ZnO	5h	10	-33,22	9,47375	-3,50653	0,13151	0
Fe6:ZnO	24h	10	Fe12:ZnO	5h	20	-58,61667	9,47375	-6,18727	2,08E-04	1
Fe6:ZnO	24h	10	Fe12:ZnO	24h	10	-4,63167	10,59197	-0,43728	1	0
Fe6:ZnO	24h	10	Fe12:ZnO	24h	20	13,75333	10,59197	1,29847	1	0
Fe6:ZnO	24h	20	Fe12:ZnO	5h	10	-46,97333	9,47375	-4,95826	0,00385	1
Fe6:ZnO	24h	20	Fe12:ZnO	5h	20	-72,37	9,47375	-7,639	8,31E-06	1
Fe6:ZnO	24h	20	Fe12:ZnO	24h	10	-18,385	10,59197	-1,73575	1	0
Fe6:ZnO	24h	20	Fe12:ZnO	24h	20	-1,42E-14	10,59197	-1,34E-15	1	0
Fe12:ZnO	5h	10	Fe12:ZnO	5h	20	-25,39667	9,47375	-2,68074	0,90126	0

Fe12:ZnO	5h	10	Fe12:ZnO	24h	10	28,58833	10,59197	2,69906	0,86502	0
Fe12:ZnO	5h	10	Fe12:ZnO	24h	20	46,97333	10,59197	4,4348	0,01377	1
Fe12:ZnO	5h	20	Fe12:ZnO	24h	10	53,985	10,59197	5,09678	0,00275	1
Fe12:ZnO	5h	20	Fe12:ZnO	24h	20	72,37	10,59197	6,83253	4,81E-05	1
Fe12:ZnO	24h	10	Fe12:ZnO	24h	20	18,385	11,60293	1,58451	1	0

Two Ways ANOVA on ZnO and Fe-ZnO cytotoxicity on BxPC-3 cell line (Bonferroni t-test).

Comparison between factor: Nanoparticle						
NP 1 vs. NP 2		Mean Diff.	SEM	t Value	P	Sig (P<0.05)
Fe6:ZnO	ZnO	-9,66832	8,16696	-1,18383	0,72499	0
Fe12:ZnO	ZnO	-17,05455	8,16696	-2,08824	0,12452	0
Fe12:ZnO	Fe6:ZnO	-7,38623	8,16696	-0,9044	1	0

Comparison between parameter: Dose (µg/mL)						
Dose 1 vs. Dose 2		MeanDiff	SEM	t Value	P	Sig (P<0.05)
10 µg/ml	0 µg/ml	-2,6289	10,11297	-0,25995	1	0
20 µg/ml	0 µg/ml	-4,95878	10,11297	-0,49034	1	0
20 µg/ml	10 µg/ml	-2,32988	10,11297	-0,23039	1	0
30 µg/ml	0 µg/ml	-61,65259	10,11297	-6,09639	1,19424E-6	1
30 µg/ml	10 µg/ml	-59,02369	10,11297	-5,83643	3,11541E-6	1
30 µg/ml	20 µg/ml	-56,69381	10,11297	-5,60605	7,24222E-6	1
40 µg/ml	0 µg/ml	-87,03963	11,67746	-7,45365	7,55617E-9	1
40 µg/ml	10 µg/ml	-84,41072	11,67746	-7,22852	1,75286E-8	1
40 µg/ml	20 µg/ml	-82,08085	11,67746	-7,029	3,6966E-8	1
40 µg/ml	30 µg/ml	-25,38704	11,67746	-2,17402	0,34106	0

All pairwise multiple comparisons								
NP 1	Dose (µg/mL)	NP 2	Dose (µg/mL)	MeanDiff	SEM	t Value	P	Sig (P<0.05)
ZnO	10 µg/ml	ZnO	0 µg/ml	-0,60141	17,51618	-0,03433	1	0
ZnO	20 µg/ml	ZnO	0 µg/ml	4,48506	17,51618	0,25605	1	0
ZnO	20 µg/ml	ZnO	10 µg/ml	5,08647	17,51618	0,29039	1	0
ZnO	30 µg/ml	ZnO	0 µg/ml	-41,4049	17,51618	-2,36381	1	0
ZnO	30 µg/ml	ZnO	10 µg/ml	-40,8035	17,51618	-2,32947	1	0
ZnO	30 µg/ml	ZnO	20 µg/ml	-45,89	17,51618	-2,61986	1	0
ZnO	40 µg/ml	ZnO	0 µg/ml	-71,6129	20,22595	-3,54064	0,08724	0
ZnO	40 µg/ml	ZnO	10 µg/ml	-71,0115	20,22595	-3,51091	0,09556	0

ZnO	40 µg/ml	ZnO	20 µg/ml	-76,0979	20,22595	-3,76239	0,0437	1
ZnO	40 µg/ml	ZnO	30 µg/ml	-30,208	20,22595	-1,49353	1	0
Fe6:ZnO	0 µg/ml	ZnO	0 µg/ml	0	17,51618	0	1	0
Fe6:ZnO	0 µg/ml	ZnO	10 µg/ml	0,60141	17,51618	0,03433	1	0
Fe6:ZnO	0 µg/ml	ZnO	20 µg/ml	-4,48506	17,51618	-0,25605	1	0
Fe6:ZnO	0 µg/ml	ZnO	30 µg/ml	41,40491	17,51618	2,36381	1	0
Fe6:ZnO	0 µg/ml	ZnO	40 µg/ml	71,61287	20,22595	3,54064	0,08724	0
Fe6:ZnO	10 µg/ml	ZnO	0 µg/ml	-12,9214	17,51618	-0,73768	1	0
Fe6:ZnO	10 µg/ml	ZnO	10 µg/ml	-12,32	17,51618	-0,70335	1	0
Fe6:ZnO	10 µg/ml	ZnO	20 µg/ml	-17,4065	17,51618	-0,99374	1	0
Fe6:ZnO	10 µg/ml	ZnO	30 µg/ml	28,4835	17,51618	1,62612	1	0
Fe6:ZnO	10 µg/ml	ZnO	40 µg/ml	58,69146	20,22595	2,90179	0,5628	0
Fe6:ZnO	10 µg/ml	Fe6:ZnO	0 µg/ml	-12,9214	17,51618	-0,73768	1	0
Fe6:ZnO	20 µg/ml	ZnO	0 µg/ml	-2,0716	17,51618	-0,11827	1	0
Fe6:ZnO	20 µg/ml	ZnO	10 µg/ml	-1,47019	17,51618	-0,08393	1	0
Fe6:ZnO	20 µg/ml	ZnO	20 µg/ml	-6,55666	17,51618	-0,37432	1	0
Fe6:ZnO	20 µg/ml	ZnO	30 µg/ml	39,33332	17,51618	2,24554	1	0
Fe6:ZnO	20 µg/ml	ZnO	40 µg/ml	69,54128	20,22595	3,43822	0,11919	0
Fe6:ZnO	20 µg/ml	Fe6:ZnO	0 µg/ml	-2,0716	17,51618	-0,11827	1	0
Fe6:ZnO	20 µg/ml	Fe6:ZnO	10 µg/ml	10,84981	17,51618	0,61942	1	0
Fe6:ZnO	30 µg/ml	ZnO	0 µg/ml	-54,3589	17,51618	-3,10335	0,3195	0
Fe6:ZnO	30 µg/ml	ZnO	10 µg/ml	-53,7575	17,51618	-3,06902	0,35238	0
Fe6:ZnO	30 µg/ml	ZnO	20 µg/ml	-58,8439	17,51618	-3,3594	0,15106	0
Fe6:ZnO	30 µg/ml	ZnO	30 µg/ml	-12,954	17,51618	-0,73954	1	0
Fe6:ZnO	30 µg/ml	ZnO	40 µg/ml	17,25401	20,22595	0,85306	1	0
Fe6:ZnO	30 µg/ml	Fe6:ZnO	0 µg/ml	-54,3589	17,51618	-3,10335	0,3195	0
Fe6:ZnO	30 µg/ml	Fe6:ZnO	10 µg/ml	-41,4375	17,51618	-2,36567	1	0
Fe6:ZnO	30 µg/ml	Fe6:ZnO	20 µg/ml	-52,2873	17,51618	-2,98508	0,44656	0
Fe6:ZnO	40 µg/ml	ZnO	0 µg/ml	-92,6856	20,22595	-4,58251	0,00289	1
Fe6:ZnO	40 µg/ml	ZnO	10 µg/ml	-92,0842	20,22595	-4,55278	0,0032	1
Fe6:ZnO	40 µg/ml	ZnO	20 µg/ml	-97,1707	20,22595	-4,80426	0,00134	1
Fe6:ZnO	40 µg/ml	ZnO	30 µg/ml	-51,2807	20,22595	-2,53539	1	0
Fe6:ZnO	40 µg/ml	ZnO	40 µg/ml	-21,0728	22,6133	-0,93188	1	0
Fe6:ZnO	40 µg/ml	Fe6:ZnO	0 µg/ml	-92,6856	20,22595	-4,58251	0,00289	1
Fe6:ZnO	40 µg/ml	Fe6:ZnO	10 µg/ml	-79,7642	20,22595	-3,94366	0,02447	1
Fe6:ZnO	40 µg/ml	Fe6:ZnO	20 µg/ml	-90,614	20,22595	-4,48009	0,00411	1
Fe6:ZnO	40 µg/ml	Fe6:ZnO	30 µg/ml	-38,3268	20,22595	-1,89493	1	0
Fe12:ZnO	0 µg/ml	ZnO	0 µg/ml	0	17,51618	0	1	0

Fe12:ZnO	0 µg/ml	ZnO	10 µg/ml	0,60141	17,51618	0,03433	1	0
Fe12:ZnO	0 µg/ml	ZnO	20 µg/ml	-4,48506	17,51618	-0,25605	1	0
Fe12:ZnO	0 µg/ml	ZnO	30 µg/ml	41,40491	17,51618	2,36381	1	0
Fe12:ZnO	0 µg/ml	ZnO	40 µg/ml	71,61287	20,22595	3,54064	0,08724	0
Fe12:ZnO	0 µg/ml	Fe6:ZnO	0 µg/ml	0	17,51618	0	1	0
Fe12:ZnO	0 µg/ml	Fe6:ZnO	10 µg/ml	12,92141	17,51618	0,73768	1	0
Fe12:ZnO	0 µg/ml	Fe6:ZnO	20 µg/ml	2,0716	17,51618	0,11827	1	0
Fe12:ZnO	0 µg/ml	Fe6:ZnO	30 µg/ml	54,35886	17,51618	3,10335	0,3195	0
Fe12:ZnO	0 µg/ml	Fe6:ZnO	40 µg/ml	92,68564	20,22595	4,58251	0,00289	1
Fe12:ZnO	10 µg/ml	ZnO	0 µg/ml	5,63611	17,51618	0,32177	1	0
Fe12:ZnO	10 µg/ml	ZnO	10 µg/ml	6,23752	17,51618	0,3561	1	0
Fe12:ZnO	10 µg/ml	ZnO	20 µg/ml	1,15105	17,51618	0,06571	1	0
Fe12:ZnO	10 µg/ml	ZnO	30 µg/ml	47,04103	17,51618	2,68557	1	0
Fe12:ZnO	10 µg/ml	ZnO	40 µg/ml	77,24899	20,22595	3,8193	0,03648	1
Fe12:ZnO	10 µg/ml	Fe6:ZnO	0 µg/ml	5,63611	17,51618	0,32177	1	0
Fe12:ZnO	10 µg/ml	Fe6:ZnO	10 µg/ml	18,55752	17,51618	1,05945	1	0
Fe12:ZnO	10 µg/ml	Fe6:ZnO	20 µg/ml	7,70771	17,51618	0,44003	1	0
Fe12:ZnO	10 µg/ml	Fe6:ZnO	30 µg/ml	59,99498	17,51618	3,42512	0,12401	0
Fe12:ZnO	10 µg/ml	Fe6:ZnO	40 µg/ml	98,32175	20,22595	4,86117	0,0011	1
Fe12:ZnO	10 µg/ml	Fe12:ZnO	0 µg/ml	5,63611	17,51618	0,32177	1	0
Fe12:ZnO	20 µg/ml	ZnO	0 µg/ml	-17,2898	17,51618	-0,98708	1	0
Fe12:ZnO	20 µg/ml	ZnO	10 µg/ml	-16,6884	17,51618	-0,95274	1	0
Fe12:ZnO	20 µg/ml	ZnO	20 µg/ml	-21,7749	17,51618	-1,24313	1	0
Fe12:ZnO	20 µg/ml	ZnO	30 µg/ml	24,1151	17,51618	1,37673	1	0
Fe12:ZnO	20 µg/ml	ZnO	40 µg/ml	54,32306	20,22595	2,68581	1	0
Fe12:ZnO	20 µg/ml	Fe6:ZnO	0 µg/ml	-17,2898	17,51618	-0,98708	1	0
Fe12:ZnO	20 µg/ml	Fe6:ZnO	10 µg/ml	-4,3684	17,51618	-0,24939	1	0
Fe12:ZnO	20 µg/ml	Fe6:ZnO	20 µg/ml	-15,2182	17,51618	-0,86881	1	0
Fe12:ZnO	20 µg/ml	Fe6:ZnO	30 µg/ml	37,06906	17,51618	2,11627	1	0
Fe12:ZnO	20 µg/ml	Fe6:ZnO	40 µg/ml	75,39583	20,22595	3,72768	0,04876	1
Fe12:ZnO	20 µg/ml	Fe12:ZnO	0 µg/ml	-17,2898	17,51618	-0,98708	1	0
Fe12:ZnO	20 µg/ml	Fe12:ZnO	10 µg/ml	-22,9259	17,51618	-1,30884	1	0
Fe12:ZnO	30 µg/ml	ZnO	0 µg/ml	-89,194	17,51618	-5,09209	4,85E-04	1
Fe12:ZnO	30 µg/ml	ZnO	10 µg/ml	-88,5926	17,51618	-5,05776	5,48E-04	1
Fe12:ZnO	30 µg/ml	ZnO	20 µg/ml	-93,6791	17,51618	-5,34814	1,94E-04	1
Fe12:ZnO	30 µg/ml	ZnO	30 µg/ml	-47,7891	17,51618	-2,72828	0,90017	0
Fe12:ZnO	30 µg/ml	ZnO	40 µg/ml	-17,5811	20,22595	-0,86924	1	0
Fe12:ZnO	30 µg/ml	Fe6:ZnO	0 µg/ml	-89,194	17,51618	-5,09209	4,85E-04	1

Fe12:ZnO	30 µg/ml	Fe6:ZnO	10 µg/ml	-76,2726	17,51618	-4,35441	0,00629	1
Fe12:ZnO	30 µg/ml	Fe6:ZnO	20 µg/ml	-87,1224	17,51618	-4,97382	7,38E-04	1
Fe12:ZnO	30 µg/ml	Fe6:ZnO	30 µg/ml	-34,8351	17,51618	-1,98874	1	0
Fe12:ZnO	30 µg/ml	Fe6:ZnO	40 µg/ml	3,49165	20,22595	0,17263	1	0
Fe12:ZnO	30 µg/ml	Fe12:ZnO	0 µg/ml	-89,194	17,51618	-5,09209	4,85E-04	1
Fe12:ZnO	30 µg/ml	Fe12:ZnO	10 µg/ml	-94,8301	17,51618	-5,41386	1,53E-04	1
Fe12:ZnO	30 µg/ml	Fe12:ZnO	20 µg/ml	-71,9042	17,51618	-4,10501	0,01445	1
Fe12:ZnO	40 µg/ml	ZnO	0 µg/ml	-96,8204	20,22595	-4,78694	0,00142	1
Fe12:ZnO	40 µg/ml	ZnO	10 µg/ml	-96,219	20,22595	-4,7572	0,00158	1
Fe12:ZnO	40 µg/ml	ZnO	20 µg/ml	-101,305	20,22595	-5,00869	6,53E-04	1
Fe12:ZnO	40 µg/ml	ZnO	30 µg/ml	-55,4155	20,22595	-2,73982	0,87295	0
Fe12:ZnO	40 µg/ml	ZnO	40 µg/ml	-25,2075	22,6133	-1,11472	1	0
Fe12:ZnO	40 µg/ml	Fe6:ZnO	0 µg/ml	-96,8204	20,22595	-4,78694	0,00142	1
Fe12:ZnO	40 µg/ml	Fe6:ZnO	10 µg/ml	-83,899	20,22595	-4,14809	0,01254	1
Fe12:ZnO	40 µg/ml	Fe6:ZnO	20 µg/ml	-94,7488	20,22595	-4,68452	0,00203	1
Fe12:ZnO	40 µg/ml	Fe6:ZnO	30 µg/ml	-42,4615	20,22595	-2,09936	1	0
Fe12:ZnO	40 µg/ml	Fe6:ZnO	40 µg/ml	-4,13473	22,6133	-0,18285	1	0
Fe12:ZnO	40 µg/ml	Fe12:ZnO	0 µg/ml	-96,8204	20,22595	-4,78694	0,00142	1
Fe12:ZnO	40 µg/ml	Fe12:ZnO	10 µg/ml	-102,456	20,22595	-5,0656	5,33E-04	1
Fe12:ZnO	40 µg/ml	Fe12:ZnO	20 µg/ml	-79,5306	20,22595	-3,93211	0,0254	1
Fe12:ZnO	40 µg/ml	Fe12:ZnO	30 µg/ml	-7,62638	20,22595	-0,37706	1	0

Three way ANOVA analysis on ZnO and Fe:ZnO NPs uptake experiments on BxPC-3 cell line (Bonferroni t-test).

NP						
**NP 1 vs NP 2**		**MeanDiff**	**SEM**	**t Value**	**P**	**Sig (P<0,05)**
ZnO	Fe6:ZnO	-46,3283	2,47491	-18,7192	2,4E-15	1
ZnO	Fe12:ZnO	-27,8242	2,47491	-11,2425	1,43E-10	1
Fe6:ZnO	Fe12:ZnO	18,50417	2,47491	7,47669	3,07E-07	1

Incubation time						
		**MeanDiff**	**SEM**	**t Value**	**P**	**Sig (P<0,05)**
5 h	24 h	-10,7078	2,02076	-5,29889	1,95E-05	1

Dose (µg/mL)						
		**MeanDiff**	**SEM**	**t Value**	**P**	**Sig (P<0,05)**
10 µg/ml	20 µg/ml	-29,12	2,02076	-14,4104	2,59E-13	1

All pairwise multiple comparisons										
**NP**	**Incubation**	**Dose (µg/mL)**	**NP**	**Incubation**	**Dose (µg/mL)**	**MeanDiff**	**SEM**	**t Value**	**P**	**Sig (P<0,05)**
ZnO	5 h	--	ZnO	24 h	--	-10,725	3,50006	-3,06424	0,07987	0
ZnO	5 h	--	Fe6:ZnO	5 h	--	-42,4667	3,50006	-12,1331	1,49E-10	1
ZnO	5 h	--	Fe6:ZnO	24 h	--	-60,915	3,50006	-17,404	6,14E-14	1
ZnO	5 h	--	Fe12:ZnO	5 h	--	-31,7117	3,50006	-9,06033	4,88E-08	1
ZnO	5 h	--	Fe12:ZnO	24 h	--	-34,6617	3,50006	-9,90317	8,91E-09	1
ZnO	24 h	--	Fe6:ZnO	5 h	--	-31,7417	3,50006	-9,0689	4,80E-08	1
ZnO	24 h	--	Fe6:ZnO	24 h	--	-50,19	3,50006	-14,3398	4,32E-12	1
ZnO	24 h	--	Fe12:ZnO	5 h	--	-20,9867	3,50006	-5,99609	5,16E-05	1
ZnO	24 h	--	Fe12:ZnO	24 h	--	-23,9367	3,50006	-6,83893	6,75E-06	1
Fe6:ZnO	5 h	--	Fe6:ZnO	24 h	--	-18,4483	3,50006	-5,27087	3,14E-04	1
Fe6:ZnO	5 h	--	Fe12:ZnO	5 h	--	10,755	3,50006	3,07281	0,07825	0
Fe6:ZnO	5 h	--	Fe12:ZnO	24 h	--	7,805	3,50006	2,22996	0,53045	0
Fe6:ZnO	24 h	--	Fe12:ZnO	5 h	--	29,20333	3,50006	8,34367	2,23E-07	1
Fe6:ZnO	24 h	--	Fe12:ZnO	24 h	--	26,25333	3,50006	7,50083	1,45E-06	1
Fe12:ZnO	5 h	--	Fe12:ZnO	24 h	--	-2,95	3,50006	-0,84284	1	0
ZnO	--	10 µg/ml	ZnO	--	20 µg/ml	-28,5883	3,50006	-8,16796	3,27E-07	1
ZnO	--	10 µg/ml	Fe6:ZnO	--	10 µg/ml	-47,02	3,50006	-13,4341	1,75E-11	1
ZnO	--	10 µg/ml	Fe6:ZnO	--	20 µg/ml	-74,225	3,50006	-21,2068	7,08E-16	1
ZnO	--	10 µg/ml	Fe12:ZnO	--	10 µg/ml	-26,335	3,50006	-7,52416	1,38E-06	1
ZnO	--	10 µg/ml	Fe12:ZnO	--	20 µg/ml	-57,9017	3,50006	-16,5431	1,90E-13	1
ZnO	--	20 µg/ml	Fe6:ZnO	--	10 µg/ml	-18,4317	3,50006	-5,2661	3,18E-04	1
ZnO	--	20 µg/ml	Fe6:ZnO	--	20 µg/ml	-45,6367	3,50006	-13,0388	3,30E-11	1
ZnO	--	20 µg/ml	Fe12:ZnO	--	10 µg/ml	2,25333	3,50006	0,6438	1	0
ZnO	--	20 µg/ml	Fe12:ZnO	--	20 µg/ml	-29,3133	3,50006	-8,3751	2,08E-07	1
Fe6:ZnO	--	10 µg/ml	Fe6:ZnO	--	20 µg/ml	-27,205	3,50006	-7,77273	7,85E-07	1
Fe6:ZnO	--	10 µg/ml	Fe12:ZnO	--	10 µg/ml	20,685	3,50006	5,9099	6,38E-05	1

Fe6:ZnO	--	10 µg/ml	Fe12:ZnO	--	20 µg/ml	-10,8817	3,50006	-3,109	0,07175	0
Fe6:ZnO	--	20 µg/ml	Fe12:ZnO	--	10 µg/ml	47,89	3,50006	13,68263	1,18E-11	1
Fe6:ZnO	--	20 µg/ml	Fe12:ZnO	--	20 µg/ml	16,32333	3,50006	4,66373	0,00146	1
Fe12:ZnO	--	10 µg/ml	Fe12:ZnO	--	20 µg/ml	-31,5667	3,50006	-9,0189	5,32E-08	1
--	5 h	10 µg/ml	--	5 h	20 µg/ml	-28,2189	2,85779	-9,87439	3,77E-09	1
--	5 h	10 µg/ml	--	24 h	10 µg/ml	-9,80667	2,85779	-3,43156	0,01309	1
--	5 h	10 µg/ml	--	24 h	20 µg/ml	-39,8278	2,85779	-13,9366	3,19E-12	1
--	5 h	20 µg/ml	--	24 h	10 µg/ml	18,41222	2,85779	6,44283	6,95E-06	1
--	5 h	20 µg/ml	--	24 h	20 µg/ml	-11,6089	2,85779	-4,0622	0,0027	1
--	24 h	10 µg/ml	--	24 h	20 µg/ml	-30,0211	2,85779	-10,505	1,12E-09	1
ZnO	5 h	10 µg/ml	ZnO	5 h	20 µg/ml	-25,2667	4,94983	-5,10455	0,00211	1
ZnO	5 h	10 µg/ml	ZnO	24 h	10 µg/ml	-7,40333	4,94983	-1,49567	1	0
ZnO	5 h	10 µg/ml	ZnO	24 h	20 µg/ml	-39,3133	4,94983	-7,94236	2,37E-06	1
ZnO	5 h	10 µg/ml	Fe6:ZnO	5 h	10 µg/ml	-40,1367	4,94983	-8,1087	1,64E-06	1
ZnO	5 h	10 µg/ml	Fe6:ZnO	5 h	20 µg/ml	-70,0633	4,94983	-14,1547	2,52E-11	1
ZnO	5 h	10 µg/ml	Fe6:ZnO	24 h	10 µg/ml	-61,3067	4,94983	-12,3856	4,27E-10	1
ZnO	5 h	10 µg/ml	Fe6:ZnO	24 h	20 µg/ml	-85,79	4,94983	-17,3319	2,96E-13	1
ZnO	5 h	10 µg/ml	Fe12:ZnO	5 h	10 µg/ml	-29,6133	4,94983	-5,9827	2,35E-04	1
ZnO	5 h	10 µg/ml	Fe12:ZnO	5 h	20 µg/ml	-59,0767	4,94983	-11,9351	9,23E-10	1
ZnO	5 h	10 µg/ml	Fe12:ZnO	24 h	10 µg/ml	-30,46	4,94983	-6,15375	1,54E-04	1
ZnO	5 h	10 µg/ml	Fe12:ZnO	24 h	20 µg/ml	-64,13	4,94983	-12,956	1,66E-10	1
ZnO	5 h	20 µg/ml	ZnO	24 h	10 µg/ml	17,86333	4,94983	3,60888	0,09281	0
ZnO	5 h	20 µg/ml	ZnO	24 h	20 µg/ml	-14,0467	4,94983	-2,83781	0,60011	0
ZnO	5 h	20 µg/ml	Fe6:ZnO	5 h	10 µg/ml	-14,87	4,94983	-3,00414	0,40556	0
ZnO	5 h	20 µg/ml	Fe6:ZnO	5 h	20 µg/ml	-44,7967	4,94983	-9,05014	2,19E-07	1
ZnO	5 h	20 µg/ml	Fe6:ZnO	24 h	10 µg/ml	-36,04	4,94983	-7,28106	1,06E-05	1
ZnO	5 h	20 µg/ml	Fe6:ZnO	24 h	20 µg/ml	-60,5233	4,94983	-12,2274	5,59E-10	1
ZnO	5 h	20 µg/ml	Fe12:ZnO	5 h	10 µg/ml	-4,34667	4,94983	-0,87814	1	0
ZnO	5 h	20 µg/ml	Fe12:ZnO	5 h	20 µg/ml	-33,81	4,94983	-6,83054	3,03E-05	1
ZnO	5 h	20 µg/ml	Fe12:ZnO	24 h	10 µg/ml	-5,19333	4,94983	-1,04919	1	0
ZnO	5 h	20 µg/ml	Fe12:ZnO	24 h	20 µg/ml	-38,8633	4,94983	-7,85145	2,90E-06	1
ZnO	24 h	10 µg/ml	ZnO	24 h	20 µg/ml	-31,91	4,94983	-6,44669	7,58E-05	1
ZnO	24 h	10 µg/ml	Fe6:ZnO	5 h	10 µg/ml	-32,7333	4,94983	-6,61302	5,08E-05	1
ZnO	24 h	10 µg/ml	Fe6:ZnO	5 h	20 µg/ml	-62,66	4,94983	-12,659	2,71E-10	1
ZnO	24 h	10 µg/ml	Fe6:ZnO	24 h	10 µg/ml	-53,9033	4,94983	-10,8899	5,98E-09	1
ZnO	24 h	10 µg/ml	Fe6:ZnO	24 h	20 µg/ml	-78,3867	4,94983	-15,8362	2,19E-12	1
ZnO	24 h	10 µg/ml	Fe12:ZnO	5 h	10 µg/ml	-22,21	4,94983	-4,48702	0,0101	1
ZnO	24 h	10 µg/ml	Fe12:ZnO	5 h	20 µg/ml	-51,6733	4,94983	-10,4394	1,39E-08	1
ZnO	24 h	10 µg/ml	Fe12:ZnO	24 h	10 µg/ml	-23,0567	4,94983	-4,65807	0,00654	1
ZnO	24 h	10 µg/ml	Fe12:ZnO	24 h	20 µg/ml	-56,7267	4,94983	-11,4603	2,13E-09	1
ZnO	24 h	20 µg/ml	Fe6:ZnO	5 h	10 µg/ml	-0,82333	4,94983	-0,16634	1	0
ZnO	24 h	20 µg/ml	Fe6:ZnO	5 h	20 µg/ml	-30,75	4,94983	-6,21234	1,34E-04	1
ZnO	24 h	20 µg/ml	Fe6:ZnO	24 h	10 µg/ml	-21,9933	4,94983	-4,44325	0,01129	1
ZnO	24 h	20 µg/ml	Fe6:ZnO	24 h	20 µg/ml	-46,4767	4,94983	-9,38955	1,09E-07	1
ZnO	24 h	20 µg/ml	Fe12:ZnO	5 h	10 µg/ml	9,7	4,94983	1,95966	1	0
ZnO	24 h	20 µg/ml	Fe12:ZnO	5 h	20 µg/ml	-19,7633	4,94983	-3,99273	0,03542	1

ZnO	24 h	20 µg/ml	Fe12:ZnO	24 h	10 µg/ml	8,85333	4,94983	1,78861	1	0
ZnO	24 h	20 µg/ml	Fe12:ZnO	24 h	20 µg/ml	-24,8167	4,94983	-5,01364	0,00265	1
Fe6:ZnO	5 h	10 µg/ml	Fe6:ZnO	5 h	20 µg/ml	-29,9267	4,94983	-6,046	2,01E-04	1
Fe6:ZnO	5 h	10 µg/ml	Fe6:ZnO	24 h	10 µg/ml	-21,17	4,94983	-4,27692	0,01724	1
Fe6:ZnO	5 h	10 µg/ml	Fe6:ZnO	24 h	20 µg/ml	-45,6533	4,94983	-9,22321	1,54E-07	1
Fe6:ZnO	5 h	10 µg/ml	Fe12:ZnO	5 h	10 µg/ml	10,52333	4,94983	2,126	1	0
Fe6:ZnO	5 h	10 µg/ml	Fe12:ZnO	5 h	20 µg/ml	-18,94	4,94983	-3,82639	0,05386	0
Fe6:ZnO	5 h	10 µg/ml	Fe12:ZnO	24 h	10 µg/ml	9,67667	4,94983	1,95495	1	0
Fe6:ZnO	5 h	10 µg/ml	Fe12:ZnO	24 h	20 µg/ml	-23,9933	4,94983	-4,84731	0,00404	1
Fe6:ZnO	5 h	20 µg/ml	Fe6:ZnO	24 h	10 µg/ml	8,75667	4,94983	1,76908	1	0
Fe6:ZnO	5 h	20 µg/ml	Fe6:ZnO	24 h	20 µg/ml	-15,7267	4,94983	-3,17721	0,26785	0
Fe6:ZnO	5 h	20 µg/ml	Fe12:ZnO	5 h	10 µg/ml	40,45	4,94983	8,172	1,43E-06	1
Fe6:ZnO	5 h	20 µg/ml	Fe12:ZnO	5 h	20 µg/ml	10,98667	4,94983	2,21961	1	0
Fe6:ZnO	5 h	20 µg/ml	Fe12:ZnO	24 h	10 µg/ml	39,60333	4,94983	8,00095	2,08E-06	1
Fe6:ZnO	5 h	20 µg/ml	Fe12:ZnO	24 h	20 µg/ml	5,93333	4,94983	1,19869	1	0
Fe6:ZnO	24 h	10 µg/ml	Fe6:ZnO	24 h	20 µg/ml	-24,4833	4,94983	-4,9463	0,00314	1
Fe6:ZnO	24 h	10 µg/ml	Fe12:ZnO	5 h	10 µg/ml	31,69333	4,94983	6,40291	8,42E-05	1
Fe6:ZnO	24 h	10 µg/ml	Fe12:ZnO	5 h	20 µg/ml	2,23	4,94983	0,45052	1	0
Fe6:ZnO	24 h	10 µg/ml	Fe12:ZnO	24 h	10 µg/ml	30,84667	4,94983	6,23186	1,28E-04	1
Fe6:ZnO	24 h	10 µg/ml	Fe12:ZnO	24 h	20 µg/ml	-2,82333	4,94983	-0,57039	1	0
Fe6:ZnO	24 h	20 µg/ml	Fe12:ZnO	5 h	10 µg/ml	56,17667	4,94983	11,34921	2,59E-09	1
Fe6:ZnO	24 h	20 µg/ml	Fe12:ZnO	5 h	20 µg/ml	26,71333	4,94983	5,39682	0,00101	1
Fe6:ZnO	24 h	20 µg/ml	Fe12:ZnO	24 h	10 µg/ml	55,33	4,94983	11,17816	3,53E-09	1
Fe6:ZnO	24 h	20 µg/ml	Fe12:ZnO	24 h	20 µg/ml	21,66	4,94983	4,37591	0,0134	1
Fe12:ZnO	5 h	10 µg/ml	Fe12:ZnO	5 h	20 µg/ml	-29,4633	4,94983	-5,95239	2,53E-04	1
Fe12:ZnO	5 h	10 µg/ml	Fe12:ZnO	24 h	10 µg/ml	-0,84667	4,94983	-0,17105	1	0
Fe12:ZnO	5 h	10 µg/ml	Fe12:ZnO	24 h	20 µg/ml	-34,5167	4,94983	-6,9733	2,16E-05	1
Fe12:ZnO	5 h	20 µg/ml	Fe12:ZnO	24 h	10 µg/ml	28,61667	4,94983	5,78134	3,86E-04	1
Fe12:ZnO	5 h	20 µg/ml	Fe12:ZnO	24 h	20 µg/ml	-5,05333	4,94983	-1,02091	1	0
Fe12:ZnO	24 h	10 µg/ml	Fe12:ZnO	24 h	20 µg/ml	-33,67	4,94983	-6,80225	3,24E-05	1
